# Transcriptome Profiling of Caco-2 Cancer Cell Line following Treatment with Extracts from Iodine-Biofortified Lettuce (*Lactuca sativa* L.)

**DOI:** 10.1371/journal.pone.0147336

**Published:** 2016-01-22

**Authors:** Aneta A. Koronowicz, Aneta Kopeć, Adam Master, Sylwester Smoleń, Ewa Piątkowska, Renata Bieżanowska-Kopeć, Iwona Ledwożyw-Smoleń, Łukasz Skoczylas, Roksana Rakoczy, Teresa Leszczyńska, Joanna Kapusta-Duch, Mirosław Pysz

**Affiliations:** 1 Department of Human Nutrition, Faculty of Food Technology, University of Agriculture, Krakow, Poland; 2 Department of Biochemistry and Molecular Biology, Medical Centre for Postgraduate Education, Warszawa, Poland; 3 Unit of Plant Nutrition, Institute of Plant Biology and Biotechnology, Faculty of Horticulture, University of Agriculture, Krakow, Poland; 4 Unit of Biochemistry, Institute of Plant Biology and Biotechnology, Faculty of Biotechnology and Horticulture, University of Agriculture, Krakow, Poland; 5 Department of Fruit, Vegetable and Mushroom Processing, Faculty of Food Technology, University of Agriculture, Krakow, Poland; Centro Cardiologico Monzino, ITALY

## Abstract

Although iodization of salt is the most common method used to obtain iodine-enriched food, iodine deficiency disorders are still a global health problem and profoundly affect the quality of human life. Iodine is required for the synthesis of thyroid hormones, which are crucial regulators of human metabolism, cell growth, proliferation, apoptosis and have been reported to be involved in carcinogenesis. In this study, for the first time, we evaluated the effect of iodine-biofortified lettuce on transcriptomic profile of Caco-2 cancer cell line by applying the Whole Human Genome Microarray assay. We showed 1326 differentially expressed Caco-2 transcripts after treatment with iodine-biofortified (BFL) and non-fortified (NFL) lettuce extracts. We analysed pathways, molecular functions, biological processes and protein classes based on comparison between BFL and NFL specific genes. Iodine, which was expected to act as a free ion (KI-NFL) or at least in part to be incorporated into lettuce macromolecules (BFL), differently regulated pathways of numerous transcription factors leading to different cellular effects. In this study we showed the inhibition of Caco-2 cells proliferation after treatment with BFL, but not potassium iodide (KI), and BFL-mediated induction of mitochondrial apoptosis and/or cell differentiation. Our results showed that iodine-biofortified plants can be effectively used by cells as an alternative source of this trace element. Moreover, the observed differences in action of both iodine sources may suggest a potential of BFL in cancer treatment.

## Introduction

Insufficient intake of dietary iodine may result in iodine deficiency, which can cause many adverse health effects [1, 2, 3, 4]. At present, the most effective way of controlling iodine deficiencies is a widespread iodization of table salt. However, in most industrialized countries excessive salt consumption is becoming a risk factor of cardiovascular diseases, osteoporosis or even stomach cancer [5, 6]. Furthermore, it should be considered that certain amounts of iodine may be lost during the preparation and processing of food, for example due to the use of high temperatures [7]. Inorganic iodine is volatile and it is difficult to control its loss during the storage and transport, as well as cooking, especially with the use of high-temperature oils. In this context, biofortification of vegetables with iodine during their cultivation is a considerable way to increase the iodine consumption, especially because iodine present in food can be easily assimilated [8] and almost entirely absorbed [7]. Biofortification of plants is well-known and realized through some biotechnological or agronomic methods [9, 8, 10, 11, 12]. Carrots, tomatoes, potatoes, and lettuce are consumed daily in most families. Therefore, fortifying these vegetables with iodine is an advantageous way to improve the iodine nutritional status of consumers without the risk of its excessive intake. Lettuce is a leafy vegetable, which is usually consumed raw with no risk of iodine loss, therefore it is a good crop for iodine-biofortification study [13].

In our previous studies we showed high efficiency of iodine biofortification of lettuce by soil fertilization with potassium iodide (KI). Moreover, we also observed increased iodine concentration in urine as well as in selected tissues of experimental rats, as a result of supplementing their diets with such iodine-biofortified lettuce [14].

Available literature indicates that iodine deficiency increases the risk of thyroid [15, 7], stomach [16, 17], breast [18, 19] and prostate [20] cancer. Antitumor effects of iodine may result from its antioxidant, anti-proliferative, anti-inflamatory, as well as pro-apoptotic and pro-differentiating [21, 22, 23] effects. In current study, we determined that the extracts from iodine-biofortified lettuce (BFL) reduced the proliferation of colon cancer cell line. We suspect, that it may be related to the change in expression of genes involved in proliferation and cell cycle.

To better understand underlying molecular mechanism of BFL action we applied, for the first time, a whole genome microarray analysis of the transcriptional profile of human Caco-2 cells. We compared differently regulated genes in cells treated with extracts from either iodine-biofortified or non-fortified lettuce. Finally, we determined and analyzed potentially affected cellular pathways, biological processes, molecular functions and protein classes.

## Materials and Methods

### Preparation of extracts from biofortified lettuce

Lettuce ‘Melodion’ cv. was cultivated and fertilized with KI as described by Kopeć et al. [14]. The iodine concentration was 0.50 mg/100 g dry mass (d.m.) for biofortified lettuce and 0.12 mg/100 g d.m. for control lettuce [14]. Fresh lettuce (10 g) was crushed using a homogenizer (CAT type X 120, USA) and next transferred to the Erlenmaier flask with water in temperature 90–100°C. Lettuce materials were extracted by shaking (Elpan, water bath shaker type 357, Poland) at 100°C temp. for 2 h, and next solution was centrifuged (Centrifuge type MPW-340, Poland). Then the part of the extracts was used for iodine measurement and other parts were stored at -80°C for cell culture studies.

### Determination of the extract iodine concentration

Digestion of 10 cm^3^ samples of lettuce extract in the mixture of 10 cm^3^ 65% HNO_3_ (superpure, Merck, Whitehouse Station, NJ, USA) and 0.8 cm^3^ 70% HClO_4_ (superpure, POCH, Gliwice, Poland) was conducted in the microwave system CEM MARS-5 Xpress. The content of iodine (I^-^) was analyzed by cold vapor generation technique with the use of high-dispersion ICP-OES (Inductively Coupled Plasma Optical Emission Spectrometry) Prodigy spectrometer—Leeman Labs, New Hampshire, Massachusetts, USA [24,25].

### Conditions of cell culture

Human colon cell line Caco-2 (colorectal adenocarcinoma; HTB-37) was purchased from the American Type Culture Collections (ATCC, Manassas, VA, USA). Cells were cultured in an incubator, under controlled conditions (temp., 37°C; air, 95%; CO_2,_ 5%), in Eagle's Minimum Essential Medium (Sigma, Saint Louis, MO, USA), with fetal bovine serum (FBS) (Life Technologies, Carlsbad, CA, USA) to a final concentration of 20%, according to ATCC procedure.

### Cell treatments

Cells were seeded in 96-well culture plates (Becton, Dickinson and Company, Warszawa, Poland) for cell viability and proliferation or 6-well culture plates (Becton, Dickinson and Company, Warszawa, Poland) for RNA isolation for 24 h, according to the protocol of Roche (BASEL, Switzerland) and A&A Biotechnology (Gdynia, Poland), respectively. After that time, growing medium was replaced by medium containing a) extract from non-fortified lettuce (control lettuce, NFL) with iodine content 27.6 μg/dm^3^, b) extract from iodine-biofortifed lettuce (BFL) with iodine content 186.7 μg/dm^3^, c) potassium iodide added to NFL (KI-NFL) in the concentration of 186.7 μg/dm^3^. A final iodine concentration in culture media was 107.33 nmol/dm^3^ for NFL and 441.37 nmol/dm^3^ for BFL. A final iodine concentration in KI-NFL group was the same as in BFL group. In all studies at least 4 wells were examined per treatment. Experiments were repeated 3 times.

### Cell viability and proliferation

Cell viability was measured using Cytotoxicity Detection Kit (LDH) (Roche, BASEL, Switzerland), according to the manufacturer's protocol. Cytotoxicity was assessed for extracts from BFL with final concentrations of iodine amounting to 147.12; 294.25 and 441.37 nmol, at time intervals of 24, 48 and 72 h and calculated according to the formula:

$$\text{Cytotoxicity }[\text{\%}]=\;\frac{\text{exp}.\text{ volue}-\text{low control}}{\text{high control}-\text{low control}}\times 100$$

Cell proliferation was determined using 5’-Bromo-2’-deoxy-uridine Labeling and Detection Kit III (Roche, BASEL, Switzerland), according to manufacturer's instruction. Proliferation was standardized to 100% of control. The analysis of samples was conducted in triplicates and in three independent experiments. Statistical analysis was performed using a two-tailed Student’s t-test.

### RNA isolation, validation, labeling and hybridization

Total RNA was isolated from the cells by using RNA isolation kit from cell cultures (A&A Biotechnology, Gdynia, Poland). RNA quantity was measured with NanoDrop (NanoDrop Technologies, USA). The analysis of final RNA quality and integrity was performed with a BioAnalyzer (Agilent Technologies, Santa Clara, CA, USA). To ensure optimal data quality, only samples with RNA integrity number (RIN) ≥8.0 were included in the analysis. The analysis of gene-expression profile was performed using SurePrint G3 Human Gene Expression 8x60K v2 Microarray (Agilent Technologies, Santa Clara, CA, USA). Each slide contained 8 microarrays representing about 50000 probe sets. The Low Input Quick Amp Labeling Kit, two-color (Agilent Technologies, Santa Clara, CA, USA) was used to amplify and label target RNA to generate complementary RNA (cRNA) for oligo microarrays used in gene expression profiling. Experiment was performed using a common reference design, where the common reference was a pool of equal amounts of RNA from control cells.

On each of two-color microarrays, we hybridized 300 ng of cRNA from the pool (labelled Cy3) and 300 ng of cRNA (labelled Cy5). In total, we ran 12 microarrays—three for each experimental group. Microarray hybridization was performed with the Gene Expression Hybridization Kit (Agilent Technologies, Santa Clara, CA, USA), according to the manufacturer's protocols. RNA Spike In Kit (Agilent Technologies, Santa Clara, CA, USA) was used as an internal control. Acquisition and analysis of hybridization intensities were performed using the Agilent DNA microarray scanner (G2565CA, Agilent Technologies, Santa Clara, CA, USA).

### Signal detection and statistical analysis

Data were extracted and background subtracted using the standard procedures contained in the Agilent Feature Extraction (FE) Software version 10.7.3.1. FE performs a Lowess normalization. Statistical analysis was performed using Gene Spring 12.6.1 software (Agilent Technologies, Santa Clara, CA, USA). Samples underwent quality control and the results showed that each sample had a similar QC metric profile. The next step was filtering probe sets by flags to remove poor quality probes (absent flags). Statistical significance of the differences was evaluated using a one way ANOVA and Tukey's HSD Post-hoc test (p < 0.05). A multiple testing correction was performed using Benjamini and Hochberg False Discovery Rate (FDR) < 5%. Microarray data were deposited at the Gene Expression Omnibus data repository under the number GSE71605 and followed MIAME requirements. To identify signaling pathways and gene functions the microarray data was analyzed using Panther Classification System—an online database.

### RT and Real-time PCR analysis

Reverse transcription was performed using 1 μg of total RNA isolated from the cells with Maxima first Strand cDNA Synthesis kit for RT-qPCR (Thermo Scientific, Waltham, MA, USA). Quantitative verification of genes was performed using the CFX96 Touch^™^ Real-Time PCR Detection System instrument (Bio Rad, Hercules, CA, USA), utilizing the SYBR Green Precision Melt Supermix kit (Bio-Rad). Conditions of individual PCR reactions were optimized for given pair of oligonucleotide primers (S1 Table, Supporting Information) on the basis of conditions as follows: 95°C, 10 min; 45 PCR cycles at 95°C, 15 s; 59°C, 15 s; 72°C, 15 s, followed by melting curve analysis (65–97°C with 0.11°C ramp rate and 5 acquisitions per 1°C). Results were normalized using *GAPDH*, *ACTB* and *HPRT* reference genes. Differences in gene expression between BFL and NFL groups were assessed by Student’s t-tests.

## Results

### Cell viability and proliferation

**Fig 1:**
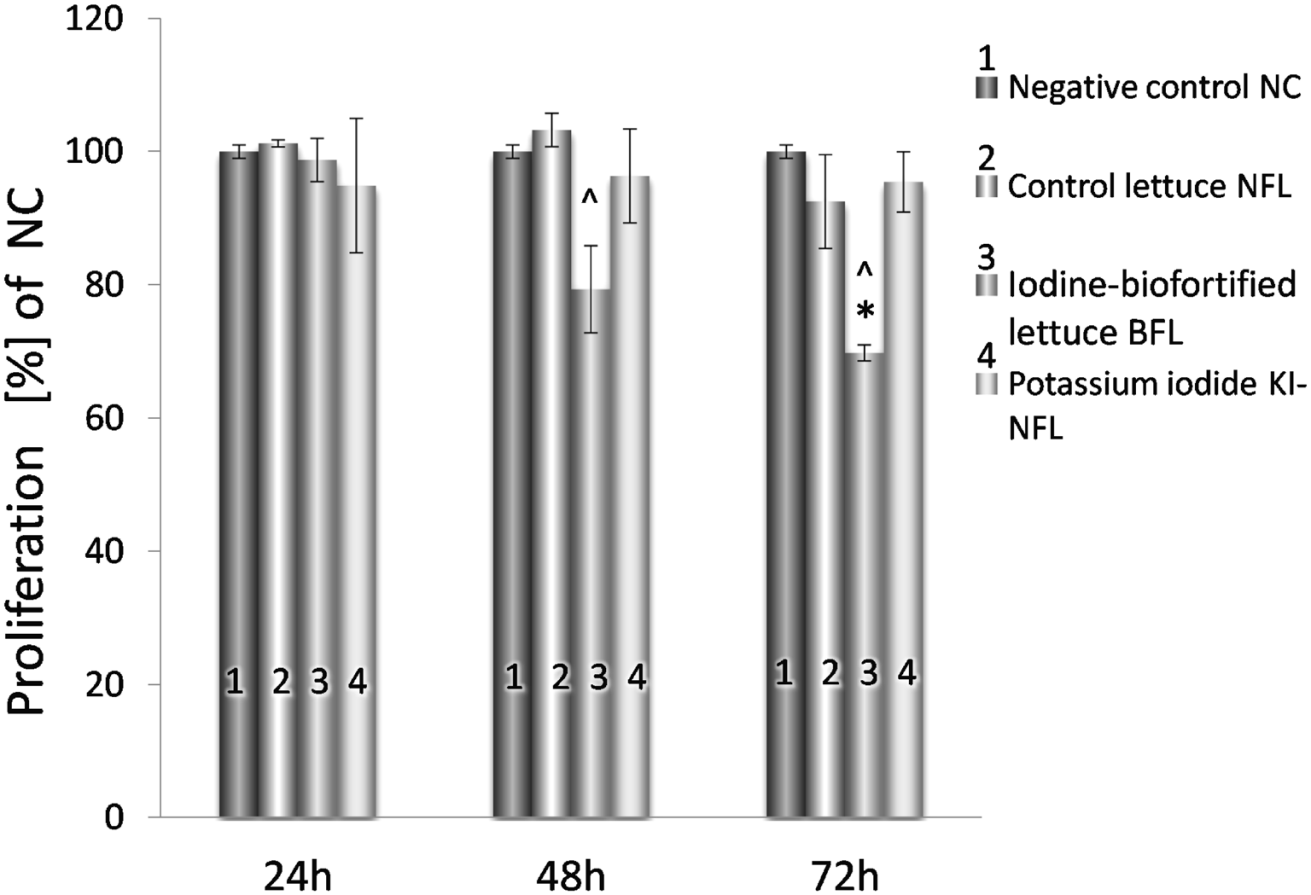
Effect of iodine-biofortified lettuce on Caco-2 cell proliferation. Values are expressed as means ± SEM for the N ≥ 9, standarized to NC as 100%. Statistical significance was based on Student's t-test * p < 0.05 versus (vs). NC and ^ p < 0.05 vs. NFL.

We determined that iodine-biofortified lettuce extract suppressed the proliferation of Caco-2 more effectively than the extract from non-fortified lettuce (Fig 1). LDH cytotoxicity test verified that observed effect was not caused by necrosis. We found no significant LDH cytotoxicity of BFL extract on Caco-2 cell line at any studied iodine concentration (data not shown). Cell proliferation was not affected by KI addition to NFL extract. Additionally, the influence of BFL and NFL on the proliferation of normal FHC cell line was examined and no decrease in cell proliferation was observed (data not shown).

### Iodine-biofortified lettuce specific genes in Caco-2 cell line

**Fig 2:**
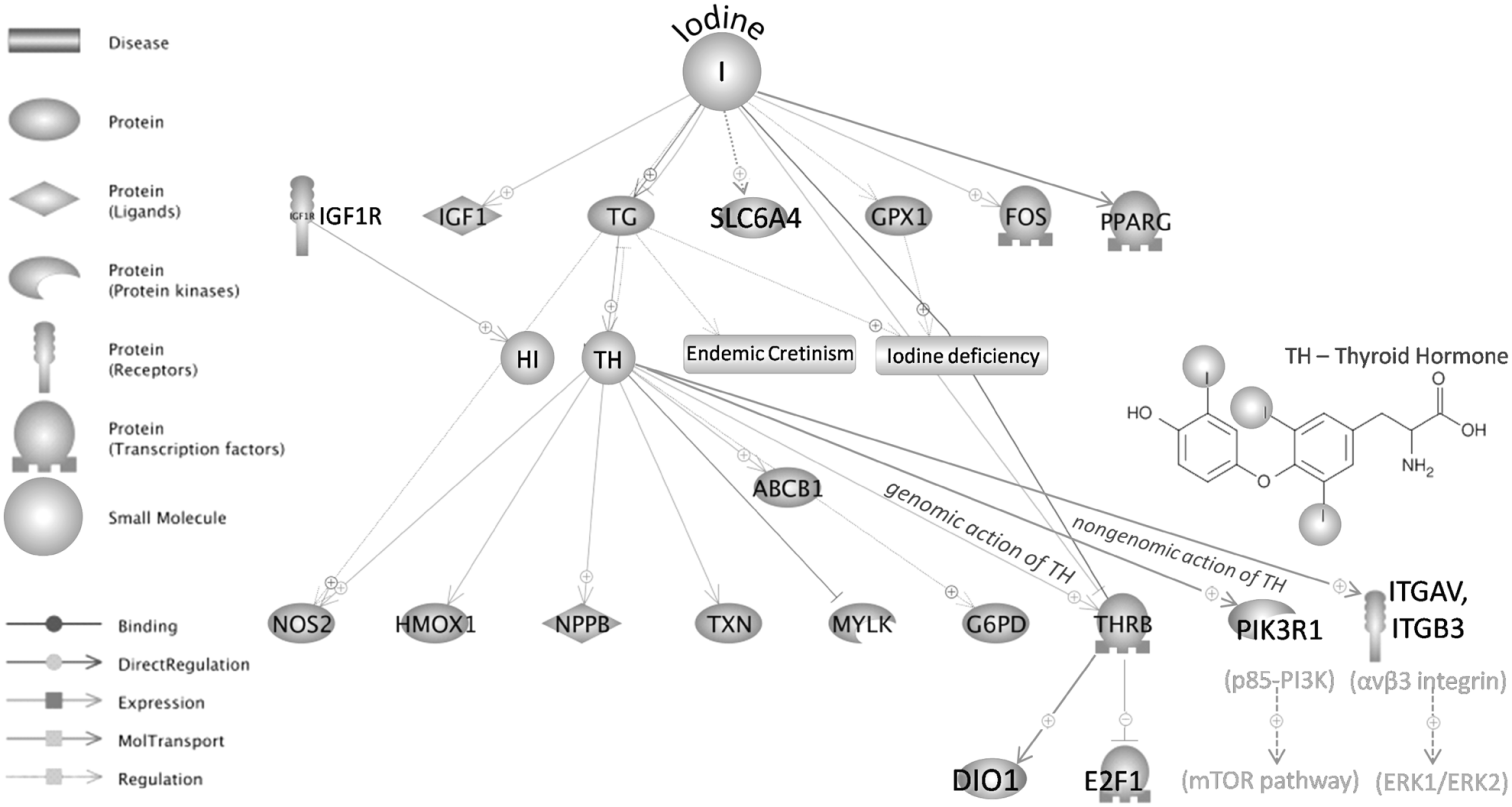
Regulation of BFL specific genes in response to iodine in Caco-2 cell line.

A total of 2603 transcripts were analyzed. We showed that about 50% of transcripts (1326 of 2603) were expressed differentially between cells treated with BFL and NFL (Table 1). The list of BFL specific transcripts is presented in Supporting Information (S2 Table, Supporting Information). Among them, using a Pathway Studio Program, we determined (Table 2) and visualized the interactions of genes and proteins in response to iodine (Figs 1 and 2).

**Table 1:** Analysis of differentially expressed transcripts between experimental groups in Caco-2 cell line.

Group Name	BFL	NFL	NC
**BFL**	**2603**•	1326*	2014*
**NFL**	1277^#^	**2603**•	2448*
**NC**	589^#^	155^#^	**2603**•

Statistical significance of treatment: p < 0.05

* transcripts differentially expressed between compared groups

^#^ common transcripts between compared groups

^•^ all the analyzed transcripts

NC negative control of Caco-2 cells

**Table 2:** Iodine-biofortified lettuce specific genes in response to iodine in Caco-2 cell line.

Gene Symbol	Adjusted p-values	FC value	Gene Name
***HMOX1***	3.31E-04	-2.54	Heme Oxygenase
***THRB***	7.62E-04	-1.64	Thyroid Hormone Receptor, Beta
***G6PD***	2.81E-03	-1.53	Glucose-6-Phosphate Dehydrogenase
***ABCB1***	4.43E-03	-1.39	Atp-Binding Cassette, Sub-Family B
***FOS***	2.03E-04	-1.37	Fbj Murine Osteosarcoma Viral Oncogene Homolog
***NOS2***	3.11E-05	-1.30	Nitric Oxide Synthase 2, Inducible
***TXN***	1.01E-03	-1.25	Thioredoxin
***PPARG***	3.14E-05	-1.08	Peroxisome Proliferator-Activated Receptor Gamma
***IGF1R***	3.25E-03	1.27	Insulin-Like Growth Factor 1 Receptor
***GPX1***	4.13E-04	1.39	Glutathione Peroxidase 1
***TG***	1.15E-03	1.45	Thyroglobulin
***NPPB***	1.53E-04	1.45	Natriuretic Peptide B
***SLC6A4***	1.22E-06	1.53	Solute Carrier Family 6
***MYLK***	4.99E-04	1.56	Myosin Light Chain Kinase

Statistical significance of treatment: p < 0.05

### Genes specifically regulated by iodine-biofortified lettuce extract in Caco-2 cell line

Interactions of BFL specific genes (S2 Table, Supporting Information) in response to iodine (Fig 2) were generated automatically using a Pathway Studio Program. As shown in Fig 2, iodine affects: *IGF1*, *TG*, *PPARG*, *GPX1*, *FOS*, *SLC6A4*, *NOS2*, and *THRB* through up-/down-regulation of their expression and/or other molecular functions. Iodine indirect action is reflected mainly through the thyroglobulin (TG), which tyrosine residues are iodinated in the synthesis pathway of thyroid hormone (TH). Thus, TG is directly involved in iodine metabolism and has been reported to be associated with numerous iodine deficiency diseases [26, 27]. According to the Pathway Studio Program, iodine, that is covalently bound to TH and its synthetic analogue (levothyroxine), can influence the expression or activity of *NOS2*, *HMOX1*, *NPPB*, *TXN*, *ABCB1*, *G6PD*, *MYLK* as well as *THRB* encoding thyroid hormone receptor beta (TRβ1). At the genomic level, TRβ1, which is a TH-liganded transcription factor, can influence the mRNA levels of multiple genes including positively regulated type 1 iodothyronine deiodinase *DIO1* and negatively regulated *E2F1* transcription factor involved in cell cycle progression (Fig 2). This receptor is also thought to be a mediator of nongenomic action of TH that is responsible for activation of plasma membrane integrin αvβ3 followed by activation of downstream pathways leading to phosphorylation of ERK1/ERK2 and TRβ1 proteins. Moreover, T3-mediated formation of cytoplasmic TRβ1 complexes with p85 subunit of PI3K may activate downstream mTOR-dependent pathways that may explain pleiotropic actions of TH [28]. All these direct and indirect relations among the genes influenced by the iodine-containing molecules may correspond, at least in part, with our results showing differences in action of iodide potassium salt (KI) and iodine that could be incorporated into macromolecules of biofortified lettuce.

**Fig 3:**
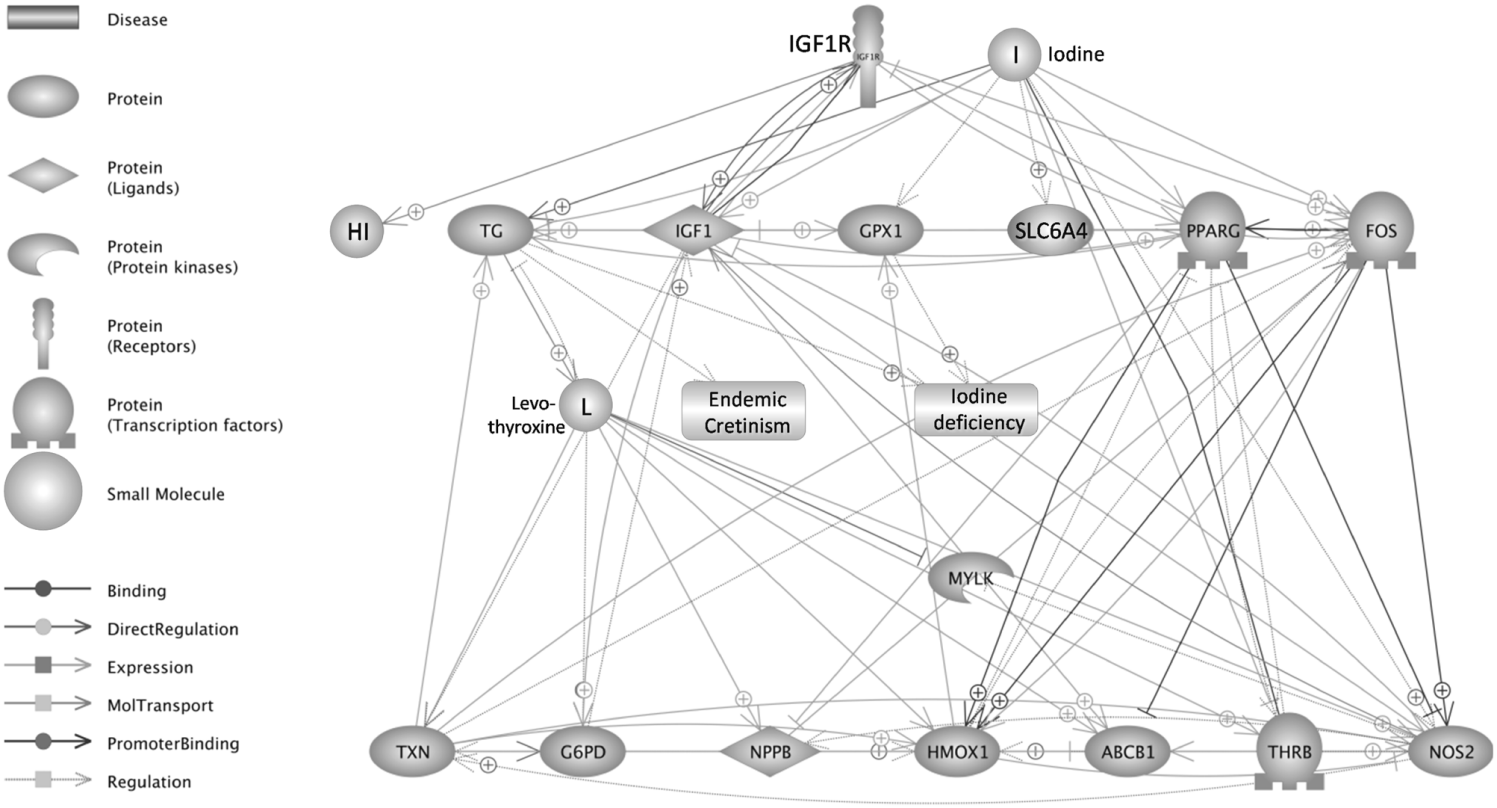
Interrelationships between BFL specific genes in response to iodine.

Additionally, interrelationships between BFL specific genes in response to iodine in Caco-2 cells (Table 2) are presented in Fig 3.

### Real-time PCR

Real-time PCR analysis was performed for nuclear receptors of thyroid hormone (TRs): thyroid hormone receptor, alpha (*THRA*) encoding TRα protein isoforms and thyroid hormone receptor, beta (*THRB*) encoding TRβ receptors, as well as for DIO1, that is positively regulated by TRs and negatively regulated E2F1. To determine whether changes in gene expression in BFL may be a result of iodide ion (I^-^) action, KI in the same concentration as in BFL was added to NFL. As a result, levels of TRβ1 mRNA were decreased in both extracts and there were no significant changes in TRα transcripts. A significant increase of DIO1 mRNA was observed in cell lines treated with BFL and KI-NFL extracts. Expression of E2F1 mRNA was decreased in BFL extract and increased in KI-NFL extract (Table 3). Data obtained with Real-Time PCR showed the same trend, verifying the microarray results.

**Table 3:** RTqPCR analysis of BFL vs. NFL and KI vs. NFL in Caco-2 cell line.

Gene Symbol	Adjusted BFL/NFL p-values	FC value BFL/NFL RTqPCR	FC value BFL/NFL Microarray	Adjusted KI/NFL p-values	FC value KI/NFL RTqPCR	Gene name
***THRA***	9.62E-01	1.01E+00^NS^	-1.01E+00^NS^	6.50E-01	1.13E+00^NS^	Thyroid Hormone Receptor, Alpha
***THRB***	*1*.*14E-03*	-1.33E+00	-1.64E+00	*4*.*44E-03*	-1.07E+00	Thyroid Hormone Receptor, Beta
***DIO1***	*1*.*86E-02*	1.89E+00	1.90E+00	*6*.*52E-03*	2.67E+00	Deiodinase, Iodothyronine, Type I
***E2F1***	*3*.*66E-02*	-1.89E+00	-1.14E+00	*2*,*32E-02*	1.58E+00	E2F Transcription Factor 1

Statistical significance of treatment: p < 0.05

NS, non-significant

### Gene Ontology molecular complete analysis

Next, we examined Gene Ontology (GO) for all BFL vs. NFL differentially regulated transcripts (S2 Table, Supporting Information), using Panther Classification System. Results obtained from analysis of the signaling pathways are presented in Table 4. GO biological processes, molecular functions and protein classes are presented in Supporting Information (S3–S5 Tables).

**Table 4:** Pathways based on BFL vs. NFL specific genes differentially regulated in Caco-2 cell line.

Pathway	The number of genes involved in pathway	The symbol of regulated gen	*p*-value
**Gonadotropin Releasing Hormone Receptor Pathway**	225	*TGFB2*, *PRKCA*, *MAP3K13*, *MAP3K2*, *MAP3K9*, *PPARG*, *IGF1*,*IRS2*, *FOS*, *RELA*, *IGF1R*, *FOSB*, *ATF3*, *NPR2*, *BMP7*, *MMP14*, *PLCB1*, *CREBBP*, *JUND*, *SRC*, *ITGA1*	1.04E-02
**Huntington Disease**	163	*VAT1*, *AP2A1*, *FAM21C*, *DNAH2*, *GRIN2C*, *ACTG2*, *NCOR2*, *TUBB6*, *TUBB2B*, *SIN3A*, *ARL4A*, *FOS*, *ACTA1*, *AKT2*, *CAPN2*, *MRPL11*, *CREBBP*, *PACSIN1*	3.37E-03
**Alzheimer Disease-Presenilin Pathway**	122	*ACTG2*, *WNT6*, *ADAM17*, *MMP24*, *WNT10A*, *ACTA1*, *PVRL1*, *NOTCH3*, *FZD4*, *PSEN2*, *APH1A*, *MMP14*, *WNT3*, *CD44*	6.58E-03
**Parkinson Disease**	107	*CLEC16A*, *UBE2L3*, *RPAP1*, *RFC5*, *UCHL1*, *PSMB10*, *CCNE2*, *NDUFV2*, *ADRBK2*, *SRC*, *PLD2*	2.99E-02
**Apoptosis Signaling Pathway**	115	*BAG3*, *PRKCA*, *TNFRSF1A*, *CRADD*, *AKT2*, *FOS*, *RELA*, *ATF3*, *XIAP*, *TNFRSF10A*, *CFLAR*	4.59E-02
**Notch Signaling Pathway**	41	*NCOR2*, *ADAM17*, *JAG1*, *APH1A*, *NOTCH3*, *PSEN2*	2.34E-02
**Cell Cycle**	22	*PSMD11*, *CCND2*, *CCNB1*, *CCNE2*, *RPA3*	6.83E-03
**Axon Guidance Mediated By Semaphorins**	22	*DPYSL3*, *NRP1*, *PLXNA1*, *DUSP5*	3.07E-02
**Pyrimidine Metabolism**	11	*DPYSL3*, *ABAT*, *ALDH6A1*	2.14E-02

Statistical significance of treatment: p < 0.05

## Discussion

To our best knowledge, our study is the first to evaluate the effect of iodine-biofortified lettuce, on transcriptome profile of Caco-2 cell line. It is also first to show the inhibition of colon cancer cells proliferation in response to iodine-biofortified lettuce extract treatment (Fig 1). We suspect that the reduction in cell viability can be caused by the presence of iodine, which was incorporated in the plant structure. The addition of KI to the NFL extract did not affect reduction of BrdU synthesis. This may suggest that in BFL, iodine is covalently bound to lipids or proteins of chloroplast membranes [29,30,31,32], although further studies are required. This organic form of iodine may interfere with pathways leading to reduced cells viability. It is indicated, that iodine treatments inhibit cell proliferation by generating iodo-lipids including 6-iodo-5-hydroxy-8,11,14-eicosatrienoic acid (an iodinated arachidonic acid) and iodohexadecanal [33, 34]. These compounds have been detected after iodine (I_2_) supplementation, and it is presumed that they may be potent activators of peroxisome proliferator-activated receptor type gamma (PPARγ) [35]. In our study, we observed the decreased of PPARγ mRNA after treatment with BFL, as well as the same tendency of PPARγ target genes (Fatty acid binding protein 4, *FABP4;* Uncoupling protein 1, *UCP-1*; Glycerol kinase, *GK*) (S2 Table, Supporting information). Therefore, observed reduction of cell proliferation may be caused by the induction of apoptosis (PPARγ-independent) and/or differentiation [36, 37, 38]. Additionally, according to other authors, bioactive compounds of lettuce have the ability to inhibit the DNA damage in N2a mouse neuroblastoma cells [39]. In our research, we did not examine the influence of BFL on genetic damage. However, taking into account the observed reduction in Caco-2 cell proliferation after BFL treatment we can assume its positive effect on the mechanisms of genotoxicity.

In this study, as the first ones, we showed the Caco-2 transcripts specifically regulated by extracts of iodine-biofortified lettuce (S2 Table, Supporting information). Based on these transcripts, we point some characteristic pathways, including the apoptosis signaling (Table 3). Analyzing the expression of apoptosis markers, differentially regulated in response to BFL vs. NFL extracts, we identified the mitochondrial apoptosis as the most probable signaling pathway. It was indicated by increased expression of pro-apoptotic Casp2 and Ripk1 Domain Containing Adaptor With Death Domain (CRADD) and decreased anti-apoptotic X-Linked Inhibitor Of Apoptosis (XIAP) and Bcl2-Associated Athanogene 3 (BAG3). Caspase-2 engages a mitochondria-dependent apoptotic pathway, by inducing mitochondrial proteins i.e. Bcl-2 and Bcl-xL (which block caspase-2), and CRADD, which induces cell death [40]. XIAP is a direct inhibitor of caspase activity [41], while increased expression of BAG3 in cancers is linked to the maintenance of cell survival, treatment resistance, and increased metastasis [42]. Our results are consistent with the reports of other authors, (i.e. on prostate and breast cancer cells), which show the induction of mitochondrial apoptotic pathway by a direct antioxidant/oxidant mitochondrial action of iodide (I^−^) and iodine (I_2_) [35, 43] or indirect formation of iodo-lipids [33, 34]. In the MCF-7 breast cancer cell line I_2_ was taken up by a facilitated diffusion system and covalently bound to lipids that, in turn, inhibited proliferation. The same study indicated that only I_2_ and 6-iodo-5-hydroxy-8,11,14-eicosatrienoic acid, but no KI, had the antiproliferative properties [44]. These observations are consistent with our results showing inhibition of Caco-2 proliferation that was noted after treatment with BFL, but no KI-NFL (Fig 1).

In this study we have observed increased levels of NOTCH3 and decreased expression of c-MYC mRNA in the BFL extracts (S2 Table, Supporting information). Pro-differentiating role of NOTCH family, including NOTCH3 has been recently described, relative to murine fibroblasts and human neurons, respectively [45, 46]. Albeit, regulation of c-MYC expression appears to play an important role in cell cycle progression and cellular differentiation. It has been shown, that T3-induced neuronal differentiation and growth arrest of neuroblastoma N2a-b cells is preceded by a decrease of c-MYC gene expression [47]. In such a case, this could explain the inhibition of Caco-2 cells proliferation after BFL extracts observed in our study; however, further research is required.

In this work we present the list of BFL vs. NFL specific genes in response to iodine (Table 2 and Fig 2) and possible links between the genes/proteins (Fig 3). Their functions, based on Panther Classification System database, are linked to iodine metabolism and circulation in organisms. An interesting increase in thyroglobulin (TG) mRNA, observed in our study, may be presumably associated with the activation of synthesis pathway that could lead to the formation of iodinated-proteins, similar to TG, produced in human and animal cells of thyroid gland. Nevertheless, we did not observe an enhanced expression of TPO peroxidase, which is responsible for iodine incorporation into tyrosine residues of the protein. On the other hand, it has been shown that iodine can be bound to amino acids of plant proteins [29, 30, 31, 32], however, there is lack of information about their metabolism, decomposition and biological function that could mimic the iodinated tyrosines released from TG proteins as thyroid hormones (THs). Thyroxine (T4) is one of those pro-hormones that is converted into active hormone—triiodothyronine (T3) in peripheral cells. In the presence of T3, Thyroid hormone Receptors (TRs) including TRβ1 (*THRB*) and TRα (*THRA*) can alter the expression of numerous genes by binding to DNA elements termed Thyroid hormone Response Elements (TRE), thus acting as transcription factors [28]. Although we observed decreased levels of the TRβ1 mRNA in response to iodine-biofortified lettuce (Table 3), our studies showed enhanced expression of positively regulated DIO1 and decreased levels of E2F1 transcript, which is negatively regulated by the TRβ1 proteins (Table 3). Moreover, we observed changes in the expression of other TR-regulated genes e.g. elevated mRNA levels of β-amyloid precursor protein APPBP) and decreased expression of MYC, CCND1, PPARG (S2 Table, Supporting information). DIO1 protein functions as an enzyme deiodinating thyroxine (T4) to active thyroid hormone—T3 and its over-expression may be correlated with high levels of iodine turnover in the cells. E2F1 is known to be a positive regulator of cell proliferation [48, 49] and its expression is shown to be supported by both MYC and CCND1 [50] Indeed, our research showed that the reduction of E2F1 expression as well as MYC and CCND1, positively correlated with reduced proliferation of Caco-2 cells (Fig 1). The apparent contradiction between lower mRNA levels of TRβ1 and the levels of its target genes may be explained by an increased activity of the TRβ1 protein as a T3-dependent transcription factor (Table 3). This explanation could be supported by previously reported lack of correlation between the TRβ1 protein/activity and its mRNA levels [51]. Thus, our results may suggest that iodine-biofortified lettuce, which was able to down-regulate the TRβ1 transcription, could also deliver a molecule enhancing TRβ1 activity; however, this hypothesis needs further studies. Although complementary action of the TRα receptors could be another explanation of observed results, our microarrays did not show any significant change in the TRα mRNA levels. On the other hand, an increase in DIO1 expression after KI-NFL (Table 3) does not exclude a direct influence of iodide ion (I^-^) in mechanisms that regulate the observed DIO1 trans-activation.

In conclusion, our research shows that iodine-biofortified lettuce regulates transcription of genes associated with cell cycle and apoptotic process leading to reduced Caco-2 cells proliferation. Although expression of some genes was found to be altered by both: BFL and NFL iodine forms, we also identified multiple genes differentially regulated, suggesting divergent mechanisms of action of iodine incorporated into lettuce macromolecules during biofortificaton process and iodine added as KI salt to non-fortified lettuce. This may also be an argument for the presence in BFL the covalently bound iodine forms that have been reported by other researchers to exert specific hormone-like action. Here we show that iodine-biofortified lettuce can be an attractive way to prevent iodine deficiency disorders. Although the above results require a confirmation at protein levels, presented microarrays are a valuable and multi-faceted source of information, especially for future studies on the potential of this iodine form in cancer treatment.

## Supporting Information

S1 Table Nucleotide sequences of primers. *THRA*, thyroid hormone receptor, alpha; *THRB*, thyroid hormone receptor, beta; *DIO1*, deiodinase, iodotyronine, type I; *E2F1*, E2F transcription factor 1; *ACTB*, actin, beta; *GAPDH*, glyceraldehyde-3-phosphate dehydrogenase; *HPRT1*, hypoxanthine phosphoribosyltransferase 1.
(DOCX)

S2 Table Iodine-biofortyficated lettuce specific transcripts. Statistical significance of treatment: p < 0.05.
(DOCX)

S3 Table GO biological processes based on BFL vs. NFL specific genes differently regulated in Caco-2 cell line. Statistical significance of treatment: p < 0.05.
(DOCX)

S4 Table GO molecular functions based on BFL vs. NFL specific genes differently regulated in Caco-2 cell line. Statistical significance of treatment: p < 0.05.
(DOCX)

S5 Table Protein classes based on BFL vs. NFL specific genes differently regulated in Caco-2 cell line. Statistical significance of treatment: p < 0.05.
(DOCX)
