# Supplementary material for: Transcriptome Profiling of Caco-2 Cancer Cell Line following Treatment with Extracts from Iodine-Biofortified Lettuce (Lactuca sativa L.)
Source: PLoS One. 2016 Jan 22;11(1):e0147336. doi: 10.1371/journal.pone.0147336 (PMC4723252; doi:10.1371/journal.pone.0147336)
Supplement: S1 Table — THRA, thyroid hormone receptor, alpha; THRB, thyroid hormone receptor, beta; DIO1, deiodinase, iodotyronine, type I; E2F1, E2F transcription factor 1; ACTB, actin, beta; GAPDH, glyceraldehyde-3-phosphate dehydrogenase; HPRT1, hypoxanthine phosphoribosyltransferase 1. (DOCX) [file pone.0147336.s001.docx]

**S1 Table. Nucleotide sequences of primers.**

| Gene | Primers forward 5’→3’ | Primers reverse 5’→3’ | Reference |
| --- | --- | --- | --- |
| *THRA* | GCTAATGTCAACAGACCGCT | CCCCTTGTACAGAATCGAACTC | NM_003250 |
| *THRB* | AACCCTTGCAGCCTTCACAC | CAGTCGCCATCGTATTCTCA | NM_001128177 |
| *DIO1* | GGAGGT GGCTGT GCATGT G | AATTCA GCACCA GTGGCC TA | NM_000792 |
| *E2F1* | CCTGCTCTCCGAGGACACTG | CGAGATCTGAAAGTTCTCCGA | NM_005225 |
| *ACTB* | GCTGGGGTGTTGAAGGTCTC | CGGCATCGTCACCAACTG | NM_001101.3 |
| *GAPDH* | ACCATCTTCCAGGAGCGAGA | GACTCCACGACGTACTCAGC | NM_001289746.1 |
| *HPRT1* | CAGAGGGCTACAATGTGATG | TGGCGTCGTGATTAGTGATG | NM_000194.2 |

### *THRA,* thyroid hormone receptor, alpha; *THRB,* thyroid hormone receptor, beta; *DIO1,* deiodinase, iodotyronine, type I; *E2F1,* E2F transcription factor 1; *ACTB,* actin, beta; *GAPDH,* glyceraldehyde-3-phosphate dehydrogenase; *HPRT1,* hypoxanthine phosphoribosyltransferase 1.
