# Supplementary material for: Transcriptome Profiling of Caco-2 Cancer Cell Line following Treatment with Extracts from Iodine-Biofortified Lettuce (Lactuca sativa L.)
Source: PLoS One. 2016 Jan 22;11(1):e0147336. doi: 10.1371/journal.pone.0147336 (PMC4723252; doi:10.1371/journal.pone.0147336)
Supplement: S2 Table — Statistical significance of treatment: p < 0.05. (DOCX) [file pone.0147336.s002.docx]

**S2 Table. Iodine-biofortyficated lettuce specific transcripts.**

| **Gene Symbol** | **Adjusted** | **FC value** | **Gene Name** |
| --- | --- | --- | --- |
|  | **p-values** |  |  |
| *AKR1C1* | 1.22E-06 | -6.97 | Aldo-Keto Reductase Family 1, Member C1 |
| *AKR1CL1* | 1.53E-07 | -6.79 | Aldo-Keto Reductase Family 1, Member C-Like 1 |
| *AKR1C4* | 2.04E-06 | -6.34 | Aldo-Keto Reductase Family 1, Member C4 |
| *AKR1C1* | 1.38E-08 | -6.21 | Aldo-Keto Reductase Family 1, Member C1 |
| *AKR1C3* | 9.49E-09 | -3.69 | Aldo-Keto Reductase Family 1, Member C3 |
| *OSGIN1* | 3.20E-04 | -2.94 | Oxidative Stress Induced Growth Inhibitor 1 |
| *AKR1B10* | 1.76E-04 | -2.80 | Aldo-Keto Reductase Family 1, Member B10 |
| *EID3* | 1.03E-03 | -2.79 | Ep300 Interacting Inhibitor Of Differentiation 3 |
| *AKR1B10* | 1.76E-04 | -2.76 | Aldo-Keto Reductase Family 1, Member B10 |
| *AKR1B15* | 9.86E-04 | -2.74 | Aldo-Keto Reductase Family 1, Member B15 |
| *XLOC_l2_007456* | 1.56E-07 | -2.73 | Broad Institute Lincrna |
| *AP5B1* | 3.95E-03 | -2.72 | Adaptor-Related Protein Complex 5, Beta 1 Subunit |
| *XLOC_l2_007427* | 2.00E-03 | -2.63 | Broad Institute Lincrna |
| *HMOX1* | 3.31E-04 | -2.54 | Heme Oxygenase |
| *PTGR1* | 7.97E-05 | -2.27 | Prostaglandin Reductase 1 |
| *MXI1* | 1.04E-03 | -2.26 | Max Interactor 1, Dimerization Protein |
| *AK8* | 1.57E-03 | -2.17 | Adenylate Kinase 8 |
| *RPL28* | 4.58E-04 | -2.15 | Ribosomal Protein L28 |
| *PITPNM1* | 2.65E-05 | -2.14 | Phosphatidylinositol Transfer Protein, Membrane-Associated 1 |
| *PTGR1* | 1.99E-05 | -2.13 | Prostaglandin Reductase 1 |
| *Af059569* | 9.62E-04 | -2.11 | Af059569 Actin Binding Protein Mayven |
| *PALM3* | 4.15E-03 | -2.09 | Paralemmin 3 |
| *SNORD1B* | 4.19E-03 | -2.09 | Small Nucleolar Rna, C/D Box 1B |
| *KLHL24* | 2.54E-04 | -2.07 | Kelch-Like Family Member 24 |
| *KIF13B* | 4.47E-03 | -2.07 | Kinesin Family Member 13B |
| *NR1D2* | 8.39E-04 | -2.03 | Nuclear Receptor Subfamily 1, Group D, Member 2 |
| *SRXN1* | 1.87E-06 | -2.02 | Sulfiredoxin 1 |
| *CYP1A1* | 5.04E-05 | -2.00 | Cytochrome P450, Family 1, Subfamily A, Polypeptide 1 |
| *SIM1* | 1.23E-04 | -2.00 | Single-Minded Family Bhlh Transcription Factor 1 |
| *DUSP5* | 2.02E-05 | -1.98 | Dual Specificity Phosphatase 5 |
| *Loc100505727* | 1.06E-03 | -1.97 | Predicted: Uncharacterized Loc100505727 |
| *LAMB2P1* | 1.88E-04 | -1.97 | Laminin, Beta 2 Pseudogene 1 |
| *AF187554* | 2.55E-03 | -1.91 | Sperm Antigen-36 Mrna, Complete Cds. |
| *GABARAPL1* | 1.22E-03 | -1.90 | Gaba |
| *LOC101927171* | 8.29E-04 | -1.90 | Predicted: Uncharacterized Loc101927171 |
| *BRWD1* | 1.54E-04 | -1.89 | Bromodomain And Wd Repeat Domain Containing 1 |
| *SLC9A1* | 1.99E-03 | -1.88 | Solute Carrier Family 9, Subfamily A |
| *LOC100507209* | 3.29E-03 | -1.88 | Cdna Flj27418 Fis, Clone Wmc07037. |
| *ABHD4* | 5.35E-05 | -1.87 | Abhydrolase Domain Containing 4 |
| *XLOC_l2_006036* | 1.33E-05 | -1.84 | Broad Institute Lincrna |
| Uncharacterized | 2.32E-03 | -1.84 | Uncharacterized |
| *UGDH* | 6.30E-04 | -1.82 | Udp-Glucose 6-Dehydrogenase |
| *TNFSF9* | 5.77E-04 | -1.81 | Tumor Necrosis Factor |
| *GDF15* | 6.43E-04 | -1.81 | Growth Differentiation Factor 15 |
| *Q9BVW3* | 1.95E-04 | -1.80 | Q9Bvw3_Human |
| *INTS6* | 1.87E-03 | -1.80 | Integrator Complex Subunit 6 |
| *CAB39L* | 1.27E-03 | -1.79 | Calcium Binding Protein 39-Like |
| *MKL2* | 1.41E-03 | -1.78 | Cdna Flj36258 Fis, Clone Thymu2002450. |
| Uncharacterized | 3.76E-03 | -1.74 | Uncharacterized |
| *ESYT3* | 3.66E-04 | -1.73 | Extended Synaptotagmin-Like Protein 3 |
| *DRD5* | 4.60E-03 | -1.73 | Dopamine Receptor D5 |
| *DNAJB4* | 1.66E-04 | -1.73 | Dnaj |
| *XLOC_011248* | 4.61E-03 | -1.73 | Broad Institute Lincrna |
| *FLCN* | 1.36E-06 | -1.72 | Folliculin |
| *GLTPD2* | 9.01E-04 | -1.70 | Glycolipid Transfer Protein Domain Containing 2 |
| *MYO15B* | 1.36E-03 | -1.70 | Myosin Xvb Pseudogene |
| *SQSTM1* | 2.04E-03 | -1.70 | Sequestosome 1 |
| *GSR* | 1.52E-04 | -1.68 | Glutathione Reductase |
| *TXNRD1* | 1.70E-05 | -1.68 | Thioredoxin Reductase 1 |
| *PWWP2B* | 2.39E-03 | -1.66 | Pwwp Domain Containing 2B |
| *CPT1A* | 3.41E-04 | -1.66 | Carnitine Palmitoyltransferase 1A |
| Uncharacterized | 7.82E-04 | -1.65 | Uncharacterized |
| *TTC39C* | 3.47E-03 | -1.64 | Tetratricopeptide Repeat Domain 39C |
| *AGPAT9* | 6.67E-07 | -1.64 | 1-Acylglycerol-3-Phosphate O-Acyltransferase 9 |
| *XLOC_002331* | 9.39E-04 | -1.64 | Broad Institute Lincrna |
| *THRB* | 7.62E-04 | -1.64 | Thyroid Hormone Receptor, Beta |
| *HS1BP3* | 1.07E-04 | -1.63 | Hcls1 Binding Protein 3, Mrna |
| *XLOC_l2_001683* | 1.32E-03 | -1.63 | Broad Institute Lincrna |
| *CLIP4* | 8.98E-04 | -1.62 | Cap-Gly Domain Containing Linker Protein Family, Member 4 |
| *GPX2* | 5.42E-06 | -1.62 | Glutathione Peroxidase 2 |
| *XLOC_l2_001771* | 1.53E-03 | -1.62 | Broad Institute Lincrna |
| *UBXN6* | 2.52E-03 | -1.60 | Ubx Domain Protein 6 |
| *DGCR14* | 1.98E-04 | -1.60 | Digeorge Syndrome Critical Region Gene 14 |
| *DEDD2* | 3.09E-03 | -1.60 | Death Effector Domain Containing 2 |
| *SLC22A18AS* | 2.50E-03 | -1.59 | Solute Carrier Family 22 |
| *C1orf116* | 8.03E-05 | -1.59 | Chromosome 1 Open Reading Frame 116 |
| *DDIT4* | 6.78E-04 | -1.59 | Dna-Damage-Inducible Transcript 4 |
| *ABCC5* | 1.19E-04 | -1.58 | Atp-Binding Cassette, Sub-Family C |
| Uncharacterized | 8.06E-04 | -1.58 | Uncharacterized |
| *NQO1* | 1.61E-03 | -1.58 | Nad |
| *FAM101A* | 3.24E-03 | -1.58 | Family With Sequence Similarity 101, Member A |
| *C19orf43* | 1.53E-03 | -1.57 | Chromosome 19 Open Reading Frame 43 |
| *ZNF263* | 2.45E-03 | -1.56 | Zinc Finger Protein 263 |
| *MAP3K9* | 4.28E-05 | -1.56 | Mitogen-Activated Protein Kinase Kinase Kinase 9 |
| *LINC00685* | 1.52E-04 | -1.56 | Long Intergenic Non-Protein Coding Rna 685 |
| *CREBRF* | 2.43E-03 | -1.56 | Creb3 Regulatory Factor |
| *C17orf59* | 4.93E-03 | -1.56 | Chromosome 17 Open Reading Frame 59 |
| *MAVS* | 3.42E-03 | -1.56 | Mitochondrial Antiviral Signaling Protein |
| *BRI3* | 3.70E-03 | -1.55 | Brain Protein I3 |
| *XLOC_003772* | 4.12E-03 | -1.55 | Broad Institute Lincrna |
| *PNRC1* | 2.09E-03 | -1.54 | Proline-Rich Nuclear Receptor Coactivator 1 |
| *XLOC_009868* | 2.54E-04 | -1.54 | Broad Institute Lincrna |
| Uncharacterized | 7.60E-04 | -1.54 | Uncharacterized |
| *ABCC2* | 3.46E-05 | -1.54 | Atp-Binding Cassette, Sub-Family C |
| *TICAM1* | 3.35E-03 | -1.54 | Toll-Like Receptor Adaptor Molecule 1 |
| *UBAP1* | 1.97E-05 | -1.54 | Ubiquitin Associated Protein 1 |
| *LOC101927661* | 4.10E-03 | -1.53 | Uncharacterized Loc101927661 |
| *G6PD* | 2.81E-03 | -1.53 | Glucose-6-Phosphate Dehydrogenase |
| *MAVS* | 3.20E-03 | -1.53 | Mitochondrial Antiviral Signaling Protein |
| *CYP1B1* | 7.88E-04 | -1.53 | Cytochrome P450, Family 1, Subfamily B, Polypeptide 1 |
| *SERTAD1* | 1.88E-03 | -1.52 | Serta Domain Containing 1 |
| *MPRIP* | 3.37E-04 | -1.52 | Myosin Phosphatase Rho Interacting Protein |
| *CACNA1B* | 1.64E-03 | -1.51 | Calcium Channel, Voltage-Dependent, N Type, Alpha 1B Subunit |
| *TBC1D22B* | 3.72E-04 | -1.51 | Tbc1 Domain Family, Member 22B |
| *LOC100129048* | 2.43E-03 | -1.51 | Cdna Flj46195 Fis, Clone Testi4006539. |
| *SPSB1* | 1.64E-03 | -1.51 | Spla/Ryanodine Receptor Domain And Socs Box Containing 1 |
| *TIPARP* | 3.34E-04 | -1.51 | Tcdd-Inducible Poly |
| *DDX52* | 5.39E-04 | -1.51 | Dead |
| *HUS1* | 4.81E-03 | -1.51 | Hus1 Checkpoint Homolog |
| *RPL28* | 4.12E-04 | -1.50 | Ribosomal Protein L28 |
| *JMY* | 2.74E-04 | -1.50 | Junction Mediating And Regulatory Protein, P53 Cofactor |
| *SC5D* | 3.07E-04 | -1.50 | Sterol-C5-Desaturase |
| *FTL* | 7.06E-05 | -1.50 | Ferritin, Light Polypeptide |
| *UCA1* | 1.79E-03 | -1.50 | Urothelial Cancer Associated 1 |
| Uncharacterized | 1.92E-03 | -1.50 | Uncharacterized |
| *KCNQ1OT1* | 2.97E-03 | -1.50 | Kcnq1 Opposite Strand/Antisense Transcript 1 |
| *ZBTB22* | 2.02E-03 | -1.49 | Zinc Finger And Btb Domain Containing 22 |
| *GULP1* | 2.17E-03 | -1.49 | Gulp, Engulfment Adaptor Ptb Domain Containing 1 |
| *UPP1* | 1.63E-03 | -1.49 | Uridine Phosphorylase 1 |
| *LOC102724105* | 4.18E-04 | -1.49 | Predicted: Uncharacterized Loc102724105 |
| *ZNF585A* | 4.36E-03 | -1.49 | Zinc Finger Protein 585A |
| *FOSB* | 2.44E-05 | -1.49 | Fbj Murine Osteosarcoma Viral Oncogene Homolog B |
| *RALGDS* | 3.60E-03 | -1.49 | Ral Guanine Nucleotide Dissociation Stimulator |
| *FTL* | 4.85E-04 | -1.48 | Ferritin, Light Polypeptide |
| *ABHD17A* | 2.04E-03 | -1.48 | Abhydrolase Domain Containing 17A |
| *LINC00630* | 2.29E-03 | -1.48 | Long Intergenic Non-Protein Coding Rna 630 |
| *PALM3* | 4.66E-04 | -1.48 | Paralemmin 3 |
| *PVT1* | 6.74E-05 | -1.48 | Pvt1 Oncogene |
| *FBRSL1* | 1.42E-03 | -1.48 | Fibrosin-Like 1 |
| *TRIM21* | 3.97E-03 | -1.48 | Tripartite Motif Containing 21 |
| *UBOX5* | 7.47E-04 | -1.47 | U-Box Domain Containing 5 |
| *MAVS* | 1.97E-03 | -1.47 | Mitochondrial Antiviral Signaling Protein |
| *FTL* | 1.51E-04 | -1.47 | Ferritin, Light Polypeptide |
| *DNMT3A* | 3.20E-03 | -1.46 | Dna |
| *VASN* | 1.30E-03 | -1.46 | Vasorin |
| *PTPRH* | 1.62E-03 | -1.46 | Protein Tyrosine Phosphatase, Receptor Type, H |
| *GCLC* | 5.11E-06 | -1.46 | Glutamate-Cysteine Ligase, Catalytic Subunit |
| *PGF* | 1.28E-03 | -1.46 | Placental Growth Factor |
| *ZNF296* | 2.35E-04 | -1.45 | Zinc Finger Protein 296 |
| *SNAI3-AS1* | 5.83E-04 | -1.45 | Snai3 Antisense Rna 1 |
| *TTC6* | 5.79E-04 | -1.45 | Tetratricopeptide Repeat Domain 6 |
| *XLOC_l2_005692* | 2.72E-03 | -1.45 | Broad Institute Lincrna |
| *BRF2* | 3.86E-03 | -1.45 | Brf2, Rna Polymerase Iii Transcription Initiation Factor 50 Kda Subunit |
| *UGT1A6* | 5.05E-04 | -1.45 | Udp Glucuronosyltransferase 1 Family, Polypeptide A6 |
| *XIAP* | 1.47E-03 | -1.45 | X-Linked Inhibitor Of Apoptosis |
| *FTHL17* | 2.72E-04 | -1.44 | Ferritin, Heavy Polypeptide-Like 17 |
| *LYSMD1* | 2.34E-03 | -1.44 | Lysm, Putative Peptidoglycan-Binding, Domain Containing 1 |
| *CARD8* | 1.95E-03 | -1.44 | Caspase Recruitment Domain Family, Member 8 |
| *THAP8* | 1.31E-03 | -1.44 | Thap Domain Containing 8 |
| *SNAI1* | 3.50E-05 | -1.44 | Snail Family Zinc Finger 1 |
| *GLA* | 5.81E-05 | -1.44 | Galactosidase, Alpha |
| *PCGF3* | 1.52E-04 | -1.43 | Polycomb Group Ring Finger 3 |
| *FOSL1* | 7.07E-06 | -1.43 | Fos-Like Antigen 1 |
| *DUSP4* | 3.23E-04 | -1.43 | Dual Specificity Phosphatase 4 |
| *XLOC_007696* | 1.00E-03 | -1.43 | Broad Institute Lincrna |
| *FTH1* | 5.64E-04 | -1.43 | Ferritin, Heavy Polypeptide 1 |
| *XLOC_l2_015478* | 6.47E-05 | -1.43 | Broad Institute Lincrna |
|  | 2.82E-03 | -1.43 | Homo Sapiens, Clone Image:3614204, Mrna. |
| *LOC145837* | 1.44E-03 | -1.43 | Uncharacterized Loc145837 |
| *FTH1* | 4.01E-04 | -1.42 | Ferritin, Heavy Polypeptide 1 |
| *TMEM92* | 3.38E-03 | -1.42 | Transmembrane Protein 92 |
| *ZNF584* | 4.07E-04 | -1.42 | Zinc Finger Protein 584 |
| Uncharacterized | 1.65E-03 | -1.42 | Uncharacterized |
| *ABL2* | 1.17E-03 | -1.42 | C-Abl Oncogene 2, Non-Receptor Tyrosine Kinase |
| *PRICKLE2* | 3.38E-05 | -1.42 | Prickle Homolog 2 |
| *ZNF134* | 6.77E-04 | -1.42 | Zinc Finger Protein 134 |
| *FTH1* | 1.85E-03 | -1.42 | Ferritin, Heavy Polypeptide 1 |
| Uncharacterized | 5.71E-04 | -1.42 | Uncharacterized |
| *BAG3* | 3.87E-03 | -1.42 | Bcl2-Associated Athanogene 3 |
| *GEM* | 1.12E-03 | -1.41 | Gtp Binding Protein Overexpressed In Skeletal Muscle |
| *RNMTL1* | 3.39E-05 | -1.41 | Rna Methyltransferase Like 1 |
| *TUBA4A* | 3.80E-04 | -1.41 | Tubulin, Alpha 4A |
| Uncharacterized | 3.51E-04 | -1.41 | Uncharacterized |
| *PRSS3* | 6.31E-05 | -1.41 | Protease, Serine, 3 |
| *RLF* | 1.47E-04 | -1.41 | Rearranged L-Myc Fusion |
| *Flc0165* | 1.85E-03 | -1.41 | Clone Flc0165 Mrna Sequence. |
| *WIPF2* | 2.66E-04 | -1.40 | Was/Wasl Interacting Protein Family, Member 2 |
| *AIFM2* | 7.91E-04 | -1.40 | Apoptosis-Inducing Factor, Mitochondrion-Associated, 2 |
| *ZNF615* | 7.68E-04 | -1.40 | Zinc Finger Protein 615 |
| *RNF44* | 2.87E-03 | -1.40 | Ring Finger Protein 44 |
| *FTH1* | 2.08E-03 | -1.40 | Ferritin, Heavy Polypeptide 1 |
| *KYNU* | 3.37E-03 | -1.40 | Kynureninase |
| *OSER1* | 5.69E-05 | -1.40 | Oxidative Stress Responsive Serine-Rich 1 |
| *ADAM17* | 7.46E-04 | -1.40 | Adam Metallopeptidase Domain 17 |
| *CHST7* | 3.12E-03 | -1.39 | Carbohydrate |
| *RELA* | 1.08E-04 | -1.39 | V-Rel Avian Reticuloendotheliosis Viral Oncogene Homolog A |
| *C2CD3* | 4.52E-05 | -1.39 | C2 Calcium-Dependent Domain Containing 3 |
| *C10orf12* | 2.26E-03 | -1.39 | Chromosome 10 Open Reading Frame 12 |
| *Pvt1* | 1.11E-04 | -1.39 | Pvt1 Oncogene |
| *ZNF329* | 5.49E-04 | -1.39 | Zinc Finger Protein 329 |
| *GK* | 3.73E-02 | -1.39 | Glycerol kinase |
| *Loc101930346* | 1.03E-03 | -1.39 | Predicted: Uncharacterized Loc101930346 |
| *ABCB1* | 4.43E-03 | -1.39 | Atp-Binding Cassette, Sub-Family B |
| *SP9* | 3.36E-03 | -1.38 | Sp9 Transcription Factor |
| *NDRG1* | 3.35E-03 | -1.38 | N-Myc Downstream Regulated 1 |
| *PLIN2* | 4.42E-05 | -1.38 | Perilipin 2 |
| *BRD3* | 1.22E-03 | -1.38 | Bromodomain Containing 3 |
| *Loc102724515* | 2.07E-03 | -1.38 | Predicted: Uncharacterized Loc102724515 |
| *KCTD21* | 5.29E-04 | -1.38 | Potassium Channel Tetramerization Domain Containing 21 |
| *NEDD9* | 4.81E-06 | -1.38 | Neural Precursor Cell Expressed, Developmentally Down-Regulated 9 |
| *RSL1D1* | 1.55E-03 | -1.38 | Ribosomal L1 Domain Containing 1 |
| *CBX1* | 6.28E-04 | -1.37 | Chromobox Homolog 1 |
| *SMEK1* | 2.44E-04 | -1.37 | Smek Homolog 1, Suppressor Of Mek1 |
| *POU3F4* | 4.08E-03 | -1.37 | Pou Class 3 Homeobox 4 |
| *PVRL1* | 2.66E-04 | -1.37 | Poliovirus Receptor-Related 1 |
| *EPAS1* | 4.63E-03 | -1.37 | Endothelial Pas Domain Protein 1 |
| *C12orf49* | 4.51E-03 | -1.37 | Chromosome 12 Open Reading Frame 49 |
| *FOS* | 2.03E-04 | -1.37 | Fbj Murine Osteosarcoma Viral Oncogene Homolog |
| *PSAPL1* | 4.85E-03 | -1.37 | Prosaposin-Like 1 |
| *GDF1* | 2.50E-05 | -1.37 | Growth Differentiation Factor 1 |
| *ZNF707* | 1.52E-04 | -1.37 | Zinc Finger Protein 707 |
| *FMNL2* | 6.68E-04 | -1.36 | Predicted: Formin-Like 2 |
| *RAB3IL1* | 1.36E-03 | -1.36 | Rab3A Interacting Protein |
| *ZHX2* | 2.48E-03 | -1.36 | Zinc Fingers And Homeoboxes 2 |
| *ABTB2* | 3.32E-07 | -1.36 | Ankyrin Repeat And Btb |
| *ZNF426* | 6.29E-04 | -1.36 | Zinc Finger Protein 426 |
| *HECA* | 2.81E-03 | -1.35 | Headcase Homolog |
| *SMOX* | 1.15E-04 | -1.35 | Spermine Oxidase |
| *JAG1* | 2.00E-04 | -1.35 | Jagged 1 |
| *KEAP1* | 1.19E-06 | -1.35 | Kelch-Like Ech-Associated Protein 1 |
| *RNF8* | 3.25E-05 | -1.35 | Ring Finger Protein 8, E3 Ubiquitin Protein Ligase |
| *FAM83B* | 7.29E-04 | -1.35 | Family With Sequence Similarity 83, Member B |
| *NR5A2* | 3.09E-03 | -1.35 | Nuclear Receptor Subfamily 5, Group A, Member 2 |
| *HS1BP3* | 4.13E-03 | -1.35 | Hcls1 Binding Protein 3 |
| *GPRC5A* | 2.56E-04 | -1.35 | G Protein-Coupled Receptor, Class C, Group 5, Member A |
| *PART1* | 3.12E-03 | -1.34 | Prostate Androgen-Regulated Transcript 1 |
| *C9orf106* | 3.75E-03 | -1.34 | Chromosome 9 Open Reading Frame 106 |
| *WAPAL* | 1.88E-04 | -1.34 | Wings Apart-Like Homolog |
| *C20orf196* | 8.23E-05 | -1.34 | Chromosome 20 Open Reading Frame 196 |
| *LOC729603* | 2.81E-03 | -1.34 | Calcineurin-Like Ef-Hand Protein 1 Pseudogene |
| *RNF25* | 9.19E-04 | -1.34 | Ring Finger Protein 25 |
| *VWA5B1* | 3.70E-03 | -1.34 | Von Willebrand Factor A Domain Containing 5B1 |
| *TNFRSF1A* | 3.58E-03 | -1.34 | Tumor Necrosis Factor Receptor Superfamily, Member 1A |
| *GFOD2* | 9.23E-04 | -1.33 | Glucose-Fructose Oxidoreductase Domain Containing 2 |
| *GLS* | 1.35E-03 | -1.33 | Glutaminase |
| *NUMBL* | 3.94E-05 | -1.33 | Numb Homolog |
| *ZFP64* | 9.77E-04 | -1.33 | Zfp64 Zinc Finger Protein |
| *PIM2* | 1.38E-03 | -1.33 | Pim-2 Oncogene |
| *MUSK* | 3.67E-04 | -1.33 | Muscle, Skeletal, Receptor Tyrosine Kinase |
| *FAM53C* | 6.91E-04 | -1.32 | Family With Sequence Similarity 53, Member C |
| *OSBP2* | 3.51E-03 | -1.32 | Oxysterol Binding Protein 2 |
| *IRS2* | 3.27E-04 | -1.32 | Insulin Receptor Substrate 2 |
| *SLC38A2* | 7.35E-04 | -1.32 | Solute Carrier Family 38, Member 2 |
| *JMJD6* | 6.96E-04 | -1.32 | Jumonji Domain Containing 6 |
| *RAB43* | 1.49E-03 | -1.32 | Rab43, Member Ras Oncogene Family |
| *C2orf49* | 1.35E-03 | -1.32 | Chromosome 2 Open Reading Frame 49 |
| *SLC18B1* | 7.87E-04 | -1.31 | Solute Carrier Family 18, Subfamily B, Member 1 |
| *TIMM22* | 1.99E-03 | -1.31 | Translocase Of Inner Mitochondrial Membrane 22 Homolog |
| *ANGEL1* | 1.57E-03 | -1.31 | Angel Homolog 1 |
| *PFKFB3* | 1.01E-03 | -1.31 | 6-Phosphofructo-2-Kinase/Fructose-2,6-Biphosphatase 3 |
| *SLC7A6OS* | 1.03E-03 | -1.31 | Solute Carrier Family 7, Member 6 Opposite Strand |
| *EGFR-AS1* | 3.23E-03 | -1.31 | Egfr Antisense Rna 1 |
| *ZNF425* | 2.23E-03 | -1.31 | Zinc Finger Protein 425 |
| *HIVEP2* | 2.79E-03 | -1.31 | Human Immunodeficiency Virus Type I Enhancer Binding Protein 2 |
| *UBL7-AS1* | 4.36E-04 | -1.30 | Ubl7 Antisense Rna 1 |
| *BRD4* | 2.17E-03 | -1.30 | Bromodomain Containing 4 |
| *INPP5A* | 2.34E-03 | -1.30 | Inositol Polyphosphate-5-Phosphatase, 40Kda |
| *TUBA4A* | 2.55E-04 | -1.30 | Tubulin, Alpha 4A |
| *SLC25A45* | 1.86E-03 | -1.30 | Solute Carrier Family 25, Member 45 |
| *BBC3* | 1.65E-04 | -1.30 | Bcl2 Binding Component 3 |
| *PRR15L* | 1.04E-03 | -1.30 | Proline Rich 15-Like |
| *ZKSCAN5* | 2.53E-03 | -1.30 | Zinc Finger With Krab And Scan Domains 5 |
| *MARVELD3* | 6.97E-04 | -1.30 | Marvel Domain Containing 3 |
| *PTPN1* | 2.86E-03 | -1.30 | Protein Tyrosine Phosphatase, Non-Receptor Type 1 |
| *ARHGEF7* | 5.34E-04 | -1.30 | Rho Guanine Nucleotide Exchange Factor |
| *ZNF317* | 4.58E-04 | -1.30 | Zinc Finger Protein 317 |
| *NOS2* | 3.11E-05 | -1.30 | Nitric Oxide Synthase 2, Inducible |
| *MEF2BNB* | 2.72E-04 | -1.30 | Mef2B Neighbor |
| *HBA2* | 1.28E-03 | -1.30 | Hemoglobin, Alpha 2 |
| *FAM118B* | 1.43E-04 | -1.30 | Family With Sequence Similarity 118, Member B |
| *ZBTB44* | 6.38E-04 | -1.29 | Zinc Finger And Btb Domain Containing 44 |
| *WIPI2* | 2.06E-03 | -1.29 | Wd Repeat Domain, Phosphoinositide Interacting 2 |
| *RTCB* | 5.65E-04 | -1.29 | Rna 2',3'-Cyclic Phosphate And 5'-Oh Ligase |
| *FAM83F* | 2.38E-03 | -1.29 | Family With Sequence Similarity 83, Member F |
| *BCL9* | 8.01E-04 | -1.29 | B-Cell Cll/Lymphoma 9 |
| *NOB1* | 1.96E-04 | -1.29 | Nin1/Rpn12 Binding Protein 1 Homolog |
| *ZKSCAN4* | 4.66E-03 | -1.29 | Zinc Finger With Krab And Scan Domains 4 |
| *ETFB* | 2.38E-03 | -1.29 | Electron-Transfer-Flavoprotein, Beta Polypeptide |
| *ATF3* | 4.07E-04 | -1.29 | Activating Transcription Factor 3 |
| *METTL16* | 7.07E-05 | -1.29 | Methyltransferase Like 16 |
| *CTDNEP1* | 2.69E-04 | -1.29 | Ctd Nuclear Envelope Phosphatase 1 |
| *DLG5* | 1.61E-03 | -1.28 | Discs, Large Homolog 5 |
| *AVPI1* | 1.37E-05 | -1.28 | Arginine Vasopressin-Induced 1 |
| *MAP3K13* | 4.34E-03 | -1.28 | Mitogen-Activated Protein Kinase Kinase Kinase 13 |
| *SAV1* | 3.15E-03 | -1.28 | Salvador Family Ww Domain Containing Protein 1 |
| *XLOC_006915* | 2.87E-04 | -1.28 | Broad Institute Lincrna |
| *SRSF11* | 7.64E-04 | -1.28 | Serine/Arginine-Rich Splicing Factor 11 |
| *LAMB3* | 4.26E-03 | -1.28 | Laminin, Beta 3 |
| *TAB3* | 1.68E-04 | -1.28 | Tgf-Beta Activated Kinase 1/Map3K7 Binding Protein 3 |
| *C1orf52* | 2.84E-03 | -1.28 | Chromosome 1 Open Reading Frame 52 |
| *LHFPL2* | 2.46E-04 | -1.28 | Lipoma Hmgic Fusion Partner-Like 2 |
| *MIEF1* | 1.39E-05 | -1.28 | Mitochondrial Elongation Factor 1 |
| *GNPDA1* | 2.53E-03 | -1.28 | Glucosamine-6-Phosphate Deaminase 1 |
| *UBALD2* | 3.17E-03 | -1.28 | Uba-Like Domain Containing 2 |
| *CREBBP* | 1.54E-03 | -1.28 | Creb Binding Protein |
| *PRDM1* | 6.66E-04 | -1.27 | Pr Domain Containing 1, With Znf Domain |
| *SNORA78* | 2.61E-03 | -1.27 | Qj88D05.X1 Nci_Cgap_Kid3 Cdna Clone Image:1866537 3', Mrna Sequence |
| *KAT7* | 6.38E-05 | -1.27 | K |
| *GSR* | 6.81E-06 | -1.27 | Glutathione Reductase |
| *RFFL* | 2.56E-03 | -1.27 | Ring Finger And Fyve-Like Domain Containing E3 Ubiquitin Protein Ligase |
| *HARBI1* | 1.80E-03 | -1.27 | Harbinger Transposase Derived 1 |
| *GULP1* | 8.32E-05 | -1.27 | Gulp, Engulfment Adaptor Ptb Domain Containing 1 |
| *VAT1* | 1.83E-03 | -1.27 | Vesicle Amine Transport 1 |
| *PUF60* | 1.31E-03 | -1.26 | Poly-U Binding Splicing Factor 60Kda |
| *UBE3C* | 4.78E-03 | -1.26 | Ubiquitin Protein Ligase E3C |
| *SMIM11* | 4.82E-03 | -1.26 | Small Integral Membrane Protein 11 |
| *GAPVD1* | 4.11E-03 | -1.26 | Gtpase Activating Protein And Vps9 Domains 1 |
| *ALU 1* | 1.41E-03 | -1.26 | Alu1_Human |
| *MAP3K2* | 1.03E-03 | -1.26 | Mitogen-Activated Protein Kinase Kinase Kinase 2 |
| *FAM83H* | 1.05E-03 | -1.26 | Family With Sequence Similarity 83, Member H |
| *ERRFI1* | 1.44E-04 | -1.26 | Erbb Receptor Feedback Inhibitor 1 |
| *USP31* | 9.43E-04 | -1.25 | Ubiquitin Specific Peptidase 31 |
| *VSIG10L* | 5.41E-04 | -1.25 | V-Set And Immunoglobulin Domain Containing 10 Like |
| *PRMT7* | 2.76E-06 | -1.25 | Protein Arginine Methyltransferase 7 |
| *ETS2* | 5.12E-05 | -1.25 | V-Ets Avian Erythroblastosis Virus E26 Oncogene Homolog 2 |
| *EIF4A1* | 1.68E-03 | -1.25 | Eukaryotic Translation Initiation Factor 4A1 |
| *PCGF1* | 7.23E-04 | -1.25 | Polycomb Group Ring Finger 1 |
| *C2orf44* | 2.00E-05 | -1.25 | Chromosome 2 Open Reading Frame 44 |
| *TXN* | 1.01E-03 | -1.25 | Thioredoxin |
| *PLAU* | 5.81E-04 | -1.25 | Plasminogen Activator, Urokinase |
| *COA7* | 1.33E-04 | -1.25 | Cytochrome C Oxidase Assembly Factor 7 |
| *Hs.292860* | 5.88E-04 | -1.25 | Cdna Flj41685 Fis, Clone Hcasm2006338 |
| *TOB1* | 4.10E-03 | -1.25 | Transducer Of Erbb2, 1 |
| *UCHL1* | 3.41E-03 | -1.25 | Ubiquitin Carboxyl-Terminal Esterase L1 |
| *ZRSR2* | 4.49E-03 | -1.25 | Zinc Finger |
| *NPC1* | 2.37E-03 | -1.25 | Niemann-Pick Disease, Type C1 |
| *DOCK9* | 1.59E-03 | -1.25 | Dedicator Of Cytokinesis 9 |
| *HCG18* | 4.15E-03 | -1.25 | Hla Complex Group 18 |
| *SLC25A51* | 9.69E-04 | -1.25 | Solute Carrier Family 25, Member 51 |
| *TATDN2* | 1.55E-04 | -1.24 | Tatd Dnase Domain Containing 2 |
| *BFAR* | 4.71E-05 | -1.24 | Bifunctional Apoptosis Regulator |
| *RNF115* | 1.67E-03 | -1.24 | Ring Finger Protein 115 |
| *SETDB1* | 2.66E-05 | -1.24 | Set Domain, Bifurcated 1 |
| *TMEM41A* | 3.02E-04 | -1.24 | Transmembrane Protein 41A |
| *NHP2L1* | 1.23E-03 | -1.24 | Nhp2 Non-Histone Chromosome Protein 2-Like 1 |
| *SPPL3* | 2.82E-03 | -1.24 | Signal Peptide Peptidase Like 3 |
| *ZNF410* | 2.50E-04 | -1.24 | Zinc Finger Protein 410 |
| *TM9SF1* | 6.86E-04 | -1.24 | Transmembrane 9 Superfamily Member 1 |
| *FRAT2* | 4.52E-03 | -1.24 | Frequently Rearranged In Advanced T-Cell Lymphomas 2 |
| *FBXO33* | 4.11E-04 | -1.24 | F-Box Protein 33 |
| *TAX1BP1* | 1.01E-04 | -1.24 | Tax1 |
| *YRDC* | 1.88E-04 | -1.24 | Yrdc N |
| *REXO4* | 1.85E-04 | -1.24 | Rex4, Rna Exonuclease 4 Homolog |
| *GTPBP6* | 7.19E-04 | -1.24 | Cdna Flj56662 Complete Cds, Highly Similar To Gtp Binding Protein 6 |
| *RNF10* | 4.75E-04 | -1.24 | Ring Finger Protein 10 |
| *ALAS1* | 4.93E-05 | -1.24 | Aminolevulinate, Delta-, Synthase 1 |
| *NCAPH2* | 3.09E-03 | -1.23 | Non-Smc Condensin Ii Complex, Subunit H2 |
| *ELF1* | 2.36E-03 | -1.23 | E74-Like Factor 1 |
| *ZFAND5* | 5.00E-05 | -1.23 | Zinc Finger, An1-Type Domain 5 |
| *TRIM34* | 1.02E-04 | -1.23 | Tripartite Motif Containing 34 |
| *GJB7* | 1.98E-05 | -1.23 | Gap Junction Protein, Beta 7, 25Kda |
| *TMEM87A* | 1.59E-03 | -1.23 | Transmembrane Protein 87A |
| *TUBGCP4* | 5.47E-04 | -1.23 | Tubulin, Gamma Complex Associated Protein 4 |
| *MXD1* | 2.64E-03 | -1.23 | Max Dimerization Protein 1 |
| *SIN3A* | 1.48E-03 | -1.23 | Sin3 Transcription Regulator Family Member A |
| *ZNF416* | 1.76E-03 | -1.23 | Zinc Finger Protein 416 |
| *SNAPC2* | 2.37E-03 | -1.23 | Small Nuclear Rna Activating Complex, Polypeptide 2, 45Kda |
| *XLOC_l2_002994* | 9.70E-04 | -1.23 | Broad Institute Lincrna |
| *MFSD9* | 1.25E-03 | -1.23 | Major Facilitator Superfamily Domain Containing 9 |
| *CAMK2D* | 4.93E-03 | -1.23 | Calcium/Calmodulin-Dependent Protein Kinase Ii Delta |
| *SLC48A1* | 2.01E-03 | -1.23 | Solute Carrier Family 48 |
| *UBE2L3* | 2.64E-04 | -1.23 | Ubiquitin-Conjugating Enzyme E2L 3 |
| *TMEM11* | 9.04E-04 | -1.22 | Transmembrane Protein 11 |
| *AK057067.1* | 3.09E-04 | -1.22 | Cdna Flj32505 Fis, Clone Smint1000039 |
| *XLOC_l2_006033* | 3.43E-05 | -1.22 | Broad Institute Lincrna |
| *HINFP* | 3.07E-04 | -1.22 | Histone H4 Transcription Factor |
| *PELO* | 2.02E-04 | -1.22 | Pelota Homolog |
| *HOXA13* | 8.99E-05 | -1.22 | Homeobox A13 |
| *PIAS4* | 4.78E-03 | -1.22 | Protein Inhibitor Of Activated Stat, 4 |
|  | 1.15E-03 | -1.22 | Da880232 Prost2 Cdna Clone Prost2019563 5', Mrna Sequence |
| *EIF4A1* | 3.36E-03 | -1.22 | Eukaryotic Translation Initiation Factor 4A1 |
| *ALKBH3* | 4.03E-03 | -1.22 | Alkb, Alkylation Repair Homolog 3 |
| *NCOR2* | 7.11E-04 | -1.22 | Nuclear Receptor Corepressor 2 |
| *PALM* | 8.92E-06 | -1.22 | Paralemmin |
| *DST* | 6.37E-05 | -1.22 | Dystonin |
| *PSMB5* | 7.88E-04 | -1.22 | Proteasome |
| *FZD4* | 6.65E-05 | -1.22 | Frizzled Class Receptor 4 |
| *OTUD7B* | 5.26E-04 | -1.22 | Otu Deubiquitinase 7B |
| *MED6* | 8.85E-05 | -1.22 | Mediator Complex Subunit 6 |
| *LOC642533* | 2.55E-03 | -1.22 | Predicted: Uncharacterized Loc642533 |
| *MYC* | 3.82E-05 | -1.21 | V-Myc Avian Myelocytomatosis Viral Oncogene Homolog |
| *UPF1* | 8.41E-04 | -1.21 | Upf1 Regulator Of Nonsense Transcripts Homolog |
| *SEL1L3* | 1.86E-03 | -1.21 | Sel-1 Suppressor Of Lin-12-Like 3 |
| *PLEKHA4* | 3.24E-03 | -1.21 | Pleckstrin Homology Domain Containing, Family A |
| *KCNG1* | 6.04E-04 | -1.21 | Potassium Voltage-Gated Channel, Subfamily G, Member 1 |
| *PAAF1* | 1.35E-03 | -1.21 | Proteasomal Atpase-Associated Factor 1 |
| *NET1* | 1.77E-03 | -1.21 | Neuroepithelial Cell Transforming 1 |
| *MAFF* | 1.69E-04 | -1.21 | V-Maf Avian Musculoaponeurotic Fibrosarcoma Oncogene Homolog F |
| *SCNM1* | 1.28E-04 | -1.21 | Sodium Channel Modifier 1 |
| *ZSWIM3* | 2.37E-03 | -1.21 | Zinc Finger, Swim-Type Containing 3 |
| *DCUN1D3* | 1.26E-03 | -1.21 | Dcn1, Defective In Cullin Neddylation 1, Domain Containing 3 |
| *HSPA1B* | 3.43E-03 | -1.21 | Heat Shock 70Kda Protein 1B |
| *ATF5* | 4.48E-03 | -1.21 | Activating Transcription Factor 5 |
| *NFKBIE* | 1.04E-03 | -1.21 | Nuclear Factor Of Kappa Light Polypeptide Gene Enhancer In B-Cells Inhibitor, Epsilon |
| *TMEM135* | 2.81E-03 | -1.20 | Transmembrane Protein 135 |
| *VPS37C* | 3.63E-03 | -1.20 | Vacuolar Protein Sorting 37 Homolog C |
| *TM4SF1* | 1.29E-04 | -1.20 | Transmembrane 4 L Six Family Member 1 |
| *ATP6V1C1* | 8.24E-04 | -1.20 | Atpase, H+ Transporting, Lysosomal 42Kda, V1 Subunit C1 |
| *ATG16L1* | 6.14E-05 | -1.20 | Autophagy Related 16-Like 1 |
| *CDV3* | 3.89E-05 | -1.20 | Cdv3 Homolog |
| *ZNF284* | 5.42E-05 | -1.20 | Zinc Finger Protein 284 |
| *C19orf24* | 2.31E-04 | -1.20 | Chromosome 19 Open Reading Frame 24 |
| *SPHK2* | 9.69E-05 | -1.20 | Sphingosine Kinase 2 |
| *UBFD1* | 2.29E-05 | -1.20 | Ubiquitin Family Domain Containing 1 |
| *AVEN* | 5.35E-05 | -1.20 | Apoptosis, Caspase Activation Inhibitor |
| *ZNF346* | 3.55E-03 | -1.20 | Zinc Finger Protein 346 |
| *HYAL3* | 3.42E-05 | -1.20 | Hyaluronoglucosaminidase 3 |
| *S1PR2* | 1.03E-03 | -1.20 | Sphingosine-1-Phosphate Receptor 2 |
| *RPL17* | 2.22E-04 | -1.20 | Ribosomal Protein L17 |
| *METTL21B* | 2.77E-03 | -1.20 | Methyltransferase Like 21B |
| *ZPR1* | 3.71E-04 | -1.20 | Zpr1 Zinc Finger |
| *GDAP2* | 1.10E-03 | -1.20 | Ganglioside Induced Differentiation Associated Protein 2 |
| *TRIM27* | 1.49E-03 | -1.20 | Tripartite Motif Containing 27 |
| *ZNF654* | 2.74E-03 | -1.19 | Zinc Finger Protein 654 |
| *MLX* | 6.13E-04 | -1.19 | Mlx, Max Dimerization Protein |
| *DCBLD1* | 6.87E-05 | -1.19 | Discoidin, Cub And Lccl Domain Containing 1 |
| *NEU1* | 1.67E-03 | -1.19 | Sialidase 1 |
| *CDKL3* | 3.35E-05 | -1.19 | Cyclin-Dependent Kinase-Like 3 |
| *FBXO34* | 3.40E-03 | -1.19 | F-Box Protein 34 |
| *BYSL* | 1.18E-04 | -1.19 | Bystin-Like |
| *ESF1* | 1.64E-04 | -1.19 | Esf1, Nucleolar Pre-Rrna Processing Protein, Homolog |
| *CREBZF* | 5.21E-04 | -1.19 | Creb/Atf Bzip Transcription Factor |
| *C1orf74* | 1.29E-03 | -1.19 | Chromosome 1 Open Reading Frame 74 |
| *Q53Y51* | 4.67E-04 | -1.19 | Q53Y51_Human |
| *KIAA1522* | 5.44E-04 | -1.19 | Kiaa1522 |
| *LINGO4* | 1.13E-04 | -1.19 | Leucine Rich Repeat And Ig Domain Containing 4 |
| *ZNF35* | 4.59E-03 | -1.19 | Zinc Finger Protein 35 |
| *EEFSEC* | 1.25E-04 | -1.19 | Eukaryotic Elongation Factor, Selenocysteine-Trna-Specific |
| *FLVCR1* | 8.11E-04 | -1.18 | Feline Leukemia Virus Subgroup C Cellular Receptor 1 |
| *CRTC3* | 5.31E-04 | -1.18 | Creb Regulated Transcription Coactivator 3 |
| *LHFPL3-AS2* | 3.18E-03 | -1.18 | Lhfpl3 Antisense Rna 2 |
| *RGS16* | 1.39E-05 | -1.18 | Regulator Of G-Protein Signaling 16 |
| *INTS6* | 1.27E-03 | -1.18 | Integrator Complex Subunit 6 |
| *UBIAD1* | 1.05E-03 | -1.18 | Ubia Prenyltransferase Domain Containing 1 |
| *YOD1* | 1.16E-04 | -1.18 | Yod1 Deubiquitinase |
| *ZNF622* | 1.04E-03 | -1.18 | Zinc Finger Protein 622 |
| *ZNF816* | 2.87E-04 | -1.18 | Zinc Finger Protein 816 |
| *ALDH3A2* | 8.96E-06 | -1.18 | Aldehyde Dehydrogenase 3 Family, Member A2 |
| *DRG1* | 6.34E-04 | -1.18 | Developmentally Regulated Gtp Binding Protein 1 |
| *WDR26* | 1.41E-03 | -1.18 | Wd Repeat Domain 26 |
| *NAGPA* | 2.53E-03 | -1.17 | N-Acetylglucosamine-1-Phosphodiester Alpha-N-Acetylglucosaminidase |
| *AFTPH* | 1.39E-03 | -1.17 | Aftiphilin |
| *ASB3* | 3.61E-03 | -1.17 | Ankyrin Repeat And Socs Box Containing 3 |
| *RYBP* | 9.85E-04 | -1.17 | Ring1 And Yy1 Binding Protein |
| *ANKHD1* | 7.68E-06 | -1.17 | Ankyrin Repeat And Kh Domain Containing 1 |
| *KIF17* | 3.21E-04 | -1.17 | Kinesin Family Member 17 |
| *SRPRB* | 8.56E-04 | -1.17 | Signal Recognition Particle Receptor, B Subunit |
| *CDIP1* | 4.64E-03 | -1.17 | Cell Death-Inducing P53 Target 1 |
| *KRTAP10-12* | 1.26E-03 | -1.17 | Keratin Associated Protein 10-12 |
| *ZNF259P1* | 1.66E-04 | -1.17 | Zinc Finger Protein 259 Pseudogene 1 |
| *ZNF562* | 1.88E-05 | -1.17 | Zinc Finger Protein 562 |
| *AAR2* | 4.66E-04 | -1.17 | Aar2 Splicing Factor Homolog |
| *MPV17L2* | 4.40E-03 | -1.17 | Mpv17 Mitochondrial Membrane Protein-Like 2 |
| *PPP2R2A* | 2.18E-04 | -1.17 | Protein Phosphatase 2, Regulatory Subunit B, Alpha |
| *EPN1* | 1.35E-03 | -1.17 | Epsin 1 |
| *TPST2* | 1.03E-03 | -1.17 | Tyrosylprotein Sulfotransferase 2 |
| *FAM21C* | 2.72E-04 | -1.16 | Family With Sequence Similarity 21, Member C |
| *ZZZ3* | 2.41E-04 | -1.16 | Zinc Finger, Zz-Type Containing 3 |
| *UBTD1* | 4.56E-03 | -1.16 | Ubiquitin Domain Containing 1 |
| *ATP6V0B* | 1.48E-03 | -1.16 | Atpase, H+ Transporting, Lysosomal 21Kda, V0 Subunit B |
| *MRGBP* | 1.24E-04 | -1.16 | Mrg/Morf4L Binding Protein |
| *GDA* | 1.98E-04 | -1.16 | Guanine Deaminase |
| *TTC17* | 2.53E-03 | -1.16 | Tetratricopeptide Repeat Domain 17 |
| *FAM117A* | 1.64E-03 | -1.16 | Family With Sequence Similarity 117, Member A |
| *TRIM35* | 1.07E-03 | -1.16 | Tripartite Motif Containing 35 |
| *ATP6V0D1* | 4.93E-03 | -1.16 | Atpase, H+ Transporting, Lysosomal 38Kda, V0 Subunit D1 |
| *SNTA1* | 1.40E-03 | -1.16 | Syntrophin, Alpha 1 |
| *C17orf67* | 1.85E-03 | -1.16 | Chromosome 17 Open Reading Frame 67 |
| *SPRY4* | 5.87E-05 | -1.16 | Sprouty Homolog 4 |
| *THOC3* | 2.08E-03 | -1.16 | Tho Complex 3 |
| *DNAJB6* | 2.51E-04 | -1.16 | Dnaj |
| *NUPL1* | 9.49E-05 | -1.16 | Nucleoporin Like 1 |
| *CDR2* | 4.21E-04 | -1.16 | Cerebellar Degeneration-Related Protein 2, 62Kda |
| *RPAP1* | 2.39E-03 | -1.15 | Rna Polymerase Ii Associated Protein 1 |
| *BPNT1* | 5.96E-04 | -1.15 | 3' |
| *PAFAH1B1* | 4.58E-03 | -1.15 | Platelet-Activating Factor Acetylhydrolase 1B, Regulatory Subunit 1 |
| *TNFRSF10A* | 5.98E-05 | -1.15 | Tumor Necrosis Factor Receptor Superfamily, Member 10A |
| *NDFIP1* | 6.62E-04 | -1.15 | Nedd4 Family Interacting Protein 1 |
| *TTC1* | 9.06E-04 | -1.15 | Tetratricopeptide Repeat Domain 1 |
| *COIL* | 4.65E-03 | -1.15 | Coilin |
| *PRPF4* | 1.66E-03 | -1.15 | Pre-Mrna Processing Factor 4 |
| *VDAC2* | 3.95E-05 | -1.15 | Voltage-Dependent Anion Channel 2 |
| *VDAC2* | 6.32E-04 | -1.15 | Voltage-Dependent Anion Channel 2 |
| *PSMD11* | 7.55E-05 | -1.15 | Proteasome |
| *PRPS1L1* | 7.38E-05 | -1.15 | Phosphoribosyl Pyrophosphate Synthetase 1-Like 1 |
| *SLC20A1* | 3.81E-04 | -1.15 | Solute Carrier Family 20 |
| *LEMD2* | 5.23E-04 | -1.14 | Lem Domain Containing 2 |
| *ELAC2* | 4.83E-04 | -1.14 | Elac Ribonuclease Z 2 |
| *FAM204A* | 2.31E-04 | -1.14 | Family With Sequence Similarity 204, Member A |
| *EIF3D* | 2.78E-04 | -1.14 | Eukaryotic Translation Initiation Factor 3, Subunit D |
| *XLOC_l2_005465* | 2.34E-03 | -1.14 | Broad Institute Lincrna |
| *PHF5A* | 8.37E-04 | -1.14 | Phd Finger Protein 5A |
| *RER1* | 2.47E-05 | -1.14 | Retention In Endoplasmic Reticulum Sorting Receptor 1 |
| Uncharacterized | 3.81E-04 | -1.14 | Uncharacterized |
| *C5orf30* | 3.49E-05 | -1.14 | Chromosome 5 Open Reading Frame 30 |
| *CCDC174* | 8.42E-04 | -1.14 | Coiled-Coil Domain Containing 174 |
| *RSL1D1* | 4.29E-04 | -1.13 | Ribosomal L1 Domain Containing 1 |
| *ZNF555* | 1.64E-04 | -1.13 | Zinc Finger Protein 555 |
| *PAFAH1B1* | 2.69E-04 | -1.13 | Platelet-Activating Factor Acetylhydrolase 1B, Regulatory Subunit 1 |
| *SNRNP27* | 2.13E-04 | -1.13 | Small Nuclear Ribonucleoprotein 27Kda |
| *SLC25A25* | 8.56E-05 | -1.13 | Solute Carrier Family 25 |
| *CLPX* | 2.71E-04 | -1.13 | Caseinolytic Mitochondrial Matrix Peptidase Chaperone Subunit |
| *WWP1* | 1.04E-03 | -1.13 | Ww Domain Containing E3 Ubiquitin Protein Ligase 1 |
| *ECD* | 4.06E-04 | -1.13 | Ecdysoneless Homolog |
| *AVPI1* | 1.17E-04 | -1.13 | Arginine Vasopressin-Induced 1 |
| *KCTD5* | 7.46E-04 | -1.13 | Potassium Channel Tetramerization Domain Containing 5 |
| *ZFYVE20* | 3.18E-03 | -1.13 | Zinc Finger, Fyve Domain Containing 20 |
| *RNF111* | 5.37E-04 | -1.13 | Ring Finger Protein 111 |
| *ATP5SL* | 2.05E-03 | -1.13 | Atp5S-Like |
| *C1orf50* | 2.77E-03 | -1.13 | Chromosome 1 Open Reading Frame 50 |
| *DNAJB6* | 1.61E-04 | -1.13 | Dnaj |
| *XLOC_l2_009278* | 3.67E-04 | -1.13 | Broad Institute Lincrna |
| *TM2D3* | 3.98E-04 | -1.13 | Tm2 Domain Containing 3 |
| *SENP1* | 4.23E-03 | -1.13 | Sumo1/Sentrin Specific Peptidase 1 |
| *SFXN4* | 2.95E-04 | -1.12 | Sideroflexin 4 |
| *NRBF2* | 1.04E-03 | -1.12 | Nuclear Receptor Binding Factor 2 |
| *SNRNP48* | 1.04E-03 | -1.12 | Small Nuclear Ribonucleoprotein 48Kda |
| *ANKHD1* | 1.16E-05 | -1.12 | Ankyrin Repeat And Kh Domain Containing 1 |
| *SMG8* | 1.75E-04 | -1.12 | Smg8 Nonsense Mediated Mrna Decay Factor |
| *ST13* | 1.23E-04 | -1.12 | Suppression Of Tumorigenicity 13 |
| *MYO19* | 2.04E-04 | -1.11 | Myosin Xix |
| *TSR1* | 8.20E-06 | -1.11 | Tsr1, 20S Rrna Accumulation, Homolog |
| *OXR1* | 4.59E-04 | -1.11 | Oxidation Resistance 1 |
| *THADA* | 9.47E-07 | -1.11 | Thyroid Adenoma Associated |
| *CEBPZ* | 2.89E-06 | -1.11 | Ccaat/Enhancer Binding Protein |
| *SNX11* | 2.60E-05 | -1.11 | Sorting Nexin 11 |
| *USP39* | 4.58E-03 | -1.10 | Ubiquitin Specific Peptidase 39 |
| *TEX10* | 2.93E-06 | -1.10 | Testis Expressed 10 |
| *PSMD1* | 2.05E-03 | -1.10 | Proteasome |
| Uncharacterized | 4.55E-04 | -1.10 | Uncharacterized |
| *ZFP1* | 2.37E-04 | -1.10 | Zfp1 Zinc Finger Protein |
| *SZRD1* | 9.15E-05 | -1.10 | Suz Rna Binding Domain Containing 1 |
| *ANKRD39* | 5.11E-04 | -1.10 | Ankyrin Repeat Domain 39 |
| *MTRF1* | 1.67E-04 | -1.10 | Mitochondrial Translational Release Factor 1 |
| *MYNN* | 7.08E-06 | -1.09 | Myoneurin |
| *PARL* | 1.27E-03 | -1.09 | Presenilin Associated, Rhomboid-Like |
| *GTF2E1* | 3.09E-05 | -1.09 | General Transcription Factor Iie, Polypeptide 1, Alpha 56Kda |
| *DDX20* | 3.84E-06 | -1.09 | Dead |
| *E2F6* | 4.41E-06 | -1.09 | E2F Transcription Factor 6 |
| *UMPS* | 7.44E-06 | -1.08 | Uridine Monophosphate Synthetase |
| *NUP43* | 3.19E-04 | -1.08 | Nucleoporin 43Kda |
| *CCDC127* | 4.36E-03 | -1.08 | Coiled-Coil Domain Containing 127 |
| *TRMT5* | 9.70E-06 | -1.08 | Trna Methyltransferase 5 |
| *PPARG* | 3.14E-05 | -1.08 | Peroxisome Proliferator-Activated Receptor Gamma |
| *RIOK3* | 5.41E-05 | -1.07 | Rio Kinase 3 |
| *CYP27A1* | 1.99E-03 | -1.07 | Cytochrome P450, Family 27, Subfamily A, Polypeptide 1 |
| *SAR1A* | 5.56E-06 | -1.07 | Sar1 Homolog A |
| *UCK2* | 4.15E-06 | -1.07 | Uridine-Cytidine Kinase 2 |
| *MRPL45* | 2.21E-05 | -1.05 | Mitochondrial Ribosomal Protein L45 |
| *GART* | 4.32E-06 | -1.05 | Phosphoribosylglycinamide Formyltransferase, Phosphoribosylglycinamide Synthetase, Phosphoribosylaminoimidazole Synthetase |
| *RFC5* | 5.54E-04 | 1.04 | Replication Factor C |
| *Rs10* | 1.94E-03 | 1.06 | Rs10_Human |
| *GEMIN6* | 7.29E-06 | 1.06 | Gem |
| *ANKRD42* | 2.90E-04 | 1.06 | Ankyrin Repeat Domain 42 |
| *SMC6* | 1.25E-06 | 1.07 | Structural Maintenance Of Chromosomes 6 |
| *RPS10* | 3.78E-04 | 1.07 | Ribosomal Protein S10 |
| *DROSHA* | 1.93E-04 | 1.08 | Drosha, Ribonuclease Type Iii |
| *XLOC_l2_012067* | 1.32E-03 | 1.08 | Broad Institute Lincrna |
| *LEPREL1* | 4.89E-05 | 1.08 | Leprecan-Like 1 |
| *EBNA1BP2* | 3.80E-05 | 1.09 | Ebna1 Binding Protein 2 |
| *LPCAT3* | 7.29E-05 | 1.09 | Lysophosphatidylcholine Acyltransferase 3 |
| *TTL* | 2.74E-04 | 1.09 | Tubulin Tyrosine Ligase |
| *FAM174B* | 4.56E-04 | 1.09 | Family With Sequence Similarity 174, Member B |
| *NDUFV3* | 3.34E-04 | 1.10 | Nadh Dehydrogenase |
| *CUTA* | 3.98E-05 | 1.10 | Cuta Divalent Cation Tolerance Homolog |
| *MRPL11* | 2.08E-03 | 1.10 | Mitochondrial Ribosomal Protein L11 |
| *AKR7A2* | 3.70E-04 | 1.10 | Aldo-Keto Reductase Family 7, Member A2 |
| *RPL32* | 1.61E-04 | 1.10 | Ribosomal Protein L32 |
| *FASTKD2* | 2.42E-05 | 1.10 | Fast Kinase Domains 2 |
| *MTHFD1* | 1.16E-03 | 1.10 | Methylenetetrahydrofolate Dehydrogenase |
| *FAM86B2* | 8.38E-04 | 1.10 | Family With Sequence Similarity 86, Member B2 |
| *HAS3* | 1.53E-03 | 1.10 | Hyaluronan Synthase 3 |
| *CCDC25* | 2.81E-05 | 1.11 | Coiled-Coil Domain Containing 25 |
| *TWISTNB* | 1.06E-03 | 1.11 | Twist Neighbor |
| *PTS* | 1.69E-03 | 1.11 | 6-Pyruvoyltetrahydropterin Synthase |
| *RBX1* | 2.54E-03 | 1.11 | Ring-Box 1, E3 Ubiquitin Protein Ligase |
| *PUSL1* | 1.03E-03 | 1.11 | Pseudouridylate Synthase-Like 1 |
| *CDC25C* | 7.03E-05 | 1.11 | Cell Division Cycle 25C |
| *ORC3* | 1.62E-03 | 1.11 | Origin Recognition Complex, Subunit 3 |
| *DHRSX* | 1.12E-04 | 1.11 | Dehydrogenase/Reductase |
| *TRIM45* | 7.23E-04 | 1.12 | Tripartite Motif Containing 45 |
| *AURKAPS1* | 1.79E-03 | 1.12 | Aurora Kinase A Pseudogene 1 |
| *FAM86B2* | 1.33E-03 | 1.13 | Family With Sequence Similarity 86, Member B2 |
| *TPX2* | 2.15E-04 | 1.13 | Tpx2, Microtubule-Associated |
| *FAM86A* | 1.88E-03 | 1.13 | Family With Sequence Similarity 86, Member A |
| *SCARA3* | 1.97E-05 | 1.13 | Scavenger Receptor Class A, Member 3 |
| *PDS5A* | 1.43E-04 | 1.13 | Pds5, Regulator Of Cohesion Maintenance, Homolog A |
| *PTAR1* | 1.54E-03 | 1.13 | Protein Prenyltransferase Alpha Subunit Repeat Containing 1 |
| *COMMD7* | 4.07E-04 | 1.13 | Comm Domain Containing 7 |
| *RPL22L1* | 3.61E-04 | 1.13 | Ribosomal Protein L22-Like 1 |
| *PSEN2* | 2.46E-04 | 1.13 | Presenilin 2 |
| *HCFC2* | 2.62E-04 | 1.13 | Host Cell Factor C2 |
| *DLAT* | 1.46E-03 | 1.13 | Dihydrolipoamide S-Acetyltransferase |
| *LINC00341* | 3.01E-04 | 1.13 | Long Intergenic Non-Protein Coding Rna 341 |
| *JPH1* | 3.23E-04 | 1.14 | Junctophilin 1 |
| *CWC15* | 6.49E-04 | 1.14 | Cwc15 Spliceosome-Associated Protein Homolog |
| *KIAA0020* | 1.28E-05 | 1.14 | Kiaa0020 |
| *LOC101927085* | 4.86E-04 | 1.14 | Predicted: Uncharacterized Loc101927085 |
| *CNIH4* | 5.72E-04 | 1.14 | Cornichon Family Ampa Receptor Auxiliary Protein 4 |
| *CACYBP* | 4.22E-03 | 1.14 | Calcyclin Binding Protein |
| *CAST* | 1.52E-03 | 1.14 | Calpastatin |
| *GCFC2* | 9.67E-04 | 1.14 | Gc-Rich Sequence Dna-Binding Factor 2 |
| *ZNF641* | 1.05E-04 | 1.14 | Zinc Finger Protein 641 |
| *PSMB10* | 5.09E-05 | 1.14 | Proteasome |
| *NSL1* | 5.12E-05 | 1.15 | Nsl1, Mis12 Kinetochore Complex Component |
| *C2orf72* | 4.81E-05 | 1.15 | Chromosome 2 Open Reading Frame 72 |
| *LOC729080* | 6.87E-04 | 1.15 | Glycine Cleavage System Protein H |
| *C12orf4* | 9.33E-04 | 1.15 | Chromosome 12 Open Reading Frame 4 |
| *SPCS3* | 3.77E-04 | 1.15 | Signal Peptidase Complex Subunit 3 Homolog |
| *TLCD1* | 3.15E-04 | 1.15 | Tlc Domain Containing 1 |
| *TUBB6* | 1.19E-03 | 1.15 | Tubulin, Beta 6 Class V |
| *TMBIM4* | 1.33E-04 | 1.15 | Transmembrane Bax Inhibitor Motif Containing 4 |
| *ERLEC1* | 1.67E-03 | 1.15 | Endoplasmic Reticulum Lectin 1 |
| *TBC1D7* | 1.98E-05 | 1.15 | Tbc1 Domain Family, Member 7 |
| *CSTF2* | 1.48E-03 | 1.15 | Cleavage Stimulation Factor, 3' Pre-Rna, Subunit 2, 64Kda |
| *UHRF1BP1* | 3.41E-06 | 1.15 | Uhrf1 Binding Protein 1 |
| *CDPF1* | 3.12E-03 | 1.15 | Cysteine-Rich, Dpf Motif Domain Containing 1 |
| *TTC7A* | 7.35E-06 | 1.15 | Tetratricopeptide Repeat Domain 7A |
| *BC024195* | 8.34E-05 | 1.16 | Cdna Clone Image:3840166, **** Warning: Chimeric Clone ****. |
| *ADCY3* | 2.25E-03 | 1.16 | Adenylate Cyclase 3 |
| *PXMP4* | 2.59E-03 | 1.16 | Peroxisomal Membrane Protein 4, 24Kda |
| *RFTN1* | 7.00E-05 | 1.16 | Raftlin, Lipid Raft Linker 1 |
| *PDSS1* | 3.74E-03 | 1.16 | Prenyl |
| *FTSJ1* | 4.82E-03 | 1.16 | Ftsj Rna Methyltransferase Homolog 1 |
| *TIMM17B* | 5.90E-05 | 1.16 | Translocase Of Inner Mitochondrial Membrane 17 Homolog B |
| *KRCC1* | 2.43E-03 | 1.16 | Lysine-Rich Coiled-Coil 1 |
| *TMEM25* | 1.80E-04 | 1.16 | Transmembrane Protein 25 |
| *SLC22A5* | 1.26E-03 | 1.16 | Solute Carrier Family 22 |
| *LSM8* | 2.93E-04 | 1.16 | Lsm8 Homolog, U6 Small Nuclear Rna Associated |
| *SPPL2A* | 3.94E-03 | 1.16 | Signal Peptide Peptidase Like 2A |
| *SLC7A1* | 3.21E-04 | 1.16 | Solute Carrier Family 7 |
| *MSH6* | 1.32E-03 | 1.16 | Muts Homolog 6 |
| *CARM1* | 6.63E-04 | 1.16 | Coactivator-Associated Arginine Methyltransferase 1 |
| *NDUFV2* | 4.32E-03 | 1.16 | Nadh Dehydrogenase |
| *CPSF3* | 3.13E-03 | 1.16 | Cleavage And Polyadenylation Specific Factor 3, 73Kda |
| *PRR11* | 3.14E-05 | 1.16 | Proline Rich 11 |
| *COL4A2* | 2.46E-05 | 1.17 | Collagen, Type Iv, Alpha 2 |
| *GPAA1* | 1.18E-04 | 1.17 | Glycosylphosphatidylinositol Anchor Attachment 1 |
| *MYH10* | 3.46E-03 | 1.17 | Myosin, Heavy Chain 10, Non-Muscle |
| *TNS3* | 1.15E-04 | 1.17 | Tensin 3 |
| *TMEM185B* | 1.55E-03 | 1.17 | Transmembrane Protein 185B |
| *MYO1D* | 8.15E-05 | 1.17 | Myosin Id |
| *LARP1B* | 3.97E-03 | 1.17 | La Ribonucleoprotein Domain Family, Member 1B |
| *CTDSPL* | 1.93E-03 | 1.17 | Ctd |
| *LOC100132352* | 2.55E-03 | 1.17 | Fshd Region Gene 1 Pseudogene |
| *FAM192A* | 3.37E-04 | 1.17 | Family With Sequence Similarity 192, Member A |
| *LOC641746* | 4.27E-04 | 1.17 | Glycine Cleavage System Protein H |
| *WDYHV1* | 2.98E-03 | 1.17 | Wdyhv Motif Containing 1 |
| *C8orf59* | 3.23E-05 | 1.17 | Chromosome 8 Open Reading Frame 59 |
| *FAM169A* | 2.89E-03 | 1.17 | Family With Sequence Similarity 169, Member A |
| *ELL2* | 1.69E-04 | 1.17 | Elongation Factor, Rna Polymerase Ii, 2 |
| *PTPN18* | 1.44E-03 | 1.17 | Protein Tyrosine Phosphatase, Non-Receptor Type 18 |
| *ZBED6CL* | 4.67E-03 | 1.17 | Zbed6 C-Terminal Like |
| *SMC4* | 3.56E-03 | 1.17 | Structural Maintenance Of Chromosomes 4 |
| *SHMT2* | 5.49E-06 | 1.17 | Serine Hydroxymethyltransferase 2 |
| *NAA38* | 7.20E-07 | 1.17 | N |
| *HSD17B7* | 8.07E-04 | 1.17 | Hydroxysteroid |
| *NFE2L3* | 4.09E-03 | 1.17 | Nuclear Factor, Erythroid 2-Like 3 |
| *RPL26L1* | 4.85E-05 | 1.17 | Ribosomal Protein L26-Like 1 |
| *CCNE2* | 1.99E-04 | 1.18 | Cyclin E2 |
| *TMBIM4* | 4.53E-03 | 1.18 | Transmembrane Bax Inhibitor Motif Containing 4 |
| *BUB1* | 4.07E-03 | 1.18 | Bub1 Mitotic Checkpoint Serine/Threonine Kinase |
| *TIMM8B* | 1.07E-03 | 1.18 | Translocase Of Inner Mitochondrial Membrane 8 Homolog B |
| *FH* | 2.58E-03 | 1.18 | Fumarate Hydratase |
| *PKI55* | 3.41E-04 | 1.18 | Dkfzp434H1419 |
| *TATDN3* | 3.77E-03 | 1.18 | Tatd Dnase Domain Containing 3 |
| *FA2H* | 2.04E-03 | 1.18 | Fatty Acid 2-Hydroxylase |
| *PTPRA* | 2.95E-03 | 1.18 | Protein Tyrosine Phosphatase, Receptor Type, A |
| *DTYMK* | 1.30E-04 | 1.18 | Deoxythymidylate Kinase |
| *ASNS* | 7.12E-06 | 1.18 | Asparagine Synthetase |
| *RANBP2* | 1.75E-03 | 1.18 | Ran Binding Protein 2 |
| *NFU1* | 1.51E-04 | 1.18 | Nfu1 Iron-Sulfur Cluster Scaffold Homolog |
| *FRMD5* | 1.44E-04 | 1.18 | Ferm Domain Containing 5 |
| *F8A1* | 8.33E-05 | 1.18 | Coagulation Factor Viii-Associated 1 |
| *OXSM* | 2.79E-03 | 1.18 | 3-Oxoacyl-Acp Synthase, Mitochondrial |
| *DENND2D* | 3.27E-04 | 1.18 | Denn/Madd Domain Containing 2D |
| *ABCD3* | 8.68E-04 | 1.18 | Atp-Binding Cassette, Sub-Family D |
| *PAQR8* | 2.11E-03 | 1.18 | Progestin And Adipoq Receptor Family Member Viii |
| *CFTR* | 1.33E-05 | 1.19 | Cystic Fibrosis Transmembrane Conductance Regulator |
| *CDKN3* | 3.64E-04 | 1.19 | Cyclin-Dependent Kinase Inhibitor 3 |
| *UHRF1* | 4.06E-03 | 1.19 | Ubiquitin-Like With Phd And Ring Finger Domains 1 |
| *ABHD14A* | 3.80E-03 | 1.19 | Abhydrolase Domain Containing 14A |
| *TACSTD2* | 3.58E-03 | 1.19 | Tumor-Associated Calcium Signal Transducer 2 |
| *EEF2K* | 1.04E-05 | 1.19 | Eukaryotic Elongation Factor-2 Kinase |
| *AQP11* | 7.06E-04 | 1.19 | Aquaporin 11 |
| *IPW* | 1.53E-04 | 1.19 | Imprinted In Prader-Willi Syndrome |
| *POLA1* | 4.29E-04 | 1.19 | Polymerase |
| *XLOC_l2_015203* | 4.66E-04 | 1.19 | Broad Institute Lincrna |
| *SIRPA* | 6.85E-06 | 1.19 | Signal-Regulatory Protein Alpha |
| *ING3* | 4.24E-03 | 1.19 | Inhibitor Of Growth Family, Member 3 |
| *ARL4A* | 6.63E-04 | 1.19 | Adp-Ribosylation Factor-Like 4A |
| *ADAP2* | 2.46E-04 | 1.19 | Arfgap With Dual Ph Domains 2 |
| *SORT1* | 1.49E-04 | 1.19 | Sortilin 1 |
| *NUP37* | 1.11E-03 | 1.19 | Nucleoporin 37Kda |
| *AURKA* | 2.29E-05 | 1.19 | Aurora Kinase A |
| *OXLD1* | 3.03E-03 | 1.19 | Oxidoreductase-Like Domain Containing 1 |
| *NT5M* | 2.27E-03 | 1.19 | 5',3'-Nucleotidase, Mitochondrial |
| *PLCB1* | 7.97E-04 | 1.20 | Phospholipase C, Beta 1 |
| *PPP2R5A* | 4.79E-03 | 1.20 | Protein Phosphatase 2, Regulatory Subunit B', Alpha |
| *LBR* | 5.60E-05 | 1.20 | Lamin B Receptor |
| *ST3GAL6* | 1.30E-03 | 1.20 | St3 Beta-Galactoside Alpha-2,3-Sialyltransferase 6 |
| *WNT3* | 2.95E-03 | 1.20 | Wingless-Type Mmtv Integration Site Family, Member 3 |
| *HAUS6* | 3.73E-03 | 1.20 | Haus Augmin-Like Complex, Subunit 6 |
| *MPI* | 2.98E-03 | 1.20 | Mannose Phosphate Isomerase |
| *CRAT* | 1.99E-05 | 1.20 | Carnitine O-Acetyltransferase |
| *SLX1A* | 2.38E-04 | 1.20 | Slx1 Structure-Specific Endonuclease Subunit Homolog A |
| *SIPA1L1* | 1.24E-03 | 1.20 | Signal-Induced Proliferation-Associated 1 Like 1 |
| *ANXA3* | 2.91E-03 | 1.20 | Annexin A3 |
| *HPDL* | 4.57E-04 | 1.20 | 4-Hydroxyphenylpyruvate Dioxygenase-Like |
| *CSF1R* | 1.49E-04 | 1.20 | Colony Stimulating Factor 1 Receptor |
| *STK31* | 9.14E-04 | 1.20 | Serine/Threonine Kinase 31 |
| *ECI1* | 6.14E-05 | 1.20 | Enoyl-Coa Delta Isomerase 1 |
| *AKAP11* | 1.57E-03 | 1.20 | A Kinase |
| *DDHD2* | 2.56E-03 | 1.20 | Ddhd Domain Containing 2 |
| *XLOC_l2_014504* | 1.39E-04 | 1.20 | Broad Institute Lincrna |
| *SLC37A2* | 3.23E-04 | 1.20 | Solute Carrier Family 37 |
| *TMEM136* | 1.36E-03 | 1.20 | Transmembrane Protein 136 |
| *CTPS1* | 6.11E-05 | 1.21 | Ctp Synthase 1 |
| *SNX24* | 2.92E-03 | 1.21 | Sorting Nexin 24 |
| *NFU1* | 1.79E-05 | 1.21 | Nfu1 Iron-Sulfur Cluster Scaffold Homolog |
| *SFXN2* | 3.55E-03 | 1.21 | Sideroflexin 2 |
| *P2RX2* | 6.04E-04 | 1.21 | Purinergic Receptor P2X, Ligand-Gated Ion Channel, 2 |
| *SCAF11* | 2.30E-04 | 1.21 | Sr-Related Ctd-Associated Factor 11 |
| *OLA1* | 4.16E-03 | 1.21 | Obg-Like Atpase 1 |
| *CENPI* | 7.70E-04 | 1.21 | Centromere Protein I |
| *PRC1* | 4.01E-05 | 1.21 | Protein Regulator Of Cytokinesis 1 |
| *PSAT1* | 1.74E-04 | 1.21 | Phosphoserine Aminotransferase 1 |
| *CLIC4* | 4.88E-03 | 1.21 | Chloride Intracellular Channel 4 |
| *TNNT1* | 3.53E-04 | 1.21 | Troponin T Type 1 |
| *PRIM1* | 6.75E-05 | 1.21 | Primase, Dna, Polypeptide 1 |
| *KIF1B* | 5.91E-04 | 1.21 | Kinesin Family Member 1B |
| *EPHB2* | 4.48E-05 | 1.21 | Eph Receptor B2 |
| *NR2F2* | 2.30E-04 | 1.21 | Nuclear Receptor Subfamily 2, Group F, Member 2 |
| *UBE2T* | 9.87E-05 | 1.21 | Ubiquitin-Conjugating Enzyme E2T |
| *FUT1* | 3.33E-05 | 1.21 | Fucosyltransferase 1 |
| *PXYLP1* | 7.04E-05 | 1.22 | 2-Phosphoxylose Phosphatase 1 |
| *EPS15* | 2.17E-03 | 1.22 | Epidermal Growth Factor Receptor Pathway Substrate 15 |
| *MED25* | 3.17E-03 | 1.22 | Mediator Complex Subunit 25 |
| *CDK1* | 2.21E-03 | 1.22 | Cyclin-Dependent Kinase 1 |
| *FAM84B* | 6.58E-04 | 1.22 | Family With Sequence Similarity 84, Member B |
| *UBE2Q2* | 6.42E-04 | 1.22 | Ubiquitin-Conjugating Enzyme E2Q Family Member 2 |
| *TMEM191A* | 3.76E-03 | 1.22 | Transmembrane Protein 191A |
| *CRADD* | 3.83E-03 | 1.22 | Casp2 And Ripk1 Domain Containing Adaptor With Death Domain |
| *PDK1* | 1.17E-03 | 1.22 | Pyruvate Dehydrogenase Kinase, Isozyme 1 |
| *KIAA0101* | 4.74E-03 | 1.22 | Kiaa0101 |
| *LOC100294145* | 9.88E-04 | 1.22 | Uncharacterized Loc100294145 |
| *GIT2* | 1.32E-04 | 1.22 | G Protein-Coupled Receptor Kinase Interacting Arfgap 2 |
| *KLHDC2* | 1.35E-04 | 1.22 | Kelch Domain Containing 2 |
| *HAAO* | 6.44E-04 | 1.22 | 3-Hydroxyanthranilate 3,4-Dioxygenase |
| *AP2A1* | 4.72E-03 | 1.22 | Adaptor-Related Protein Complex 2, Alpha 1 Subunit |
| *ALDH6A1* | 1.50E-03 | 1.22 | Aldehyde Dehydrogenase 6 Family, Member A1 |
| *MLH1* | 2.16E-03 | 1.22 | Mutl Homolog 1 |
| *KCNJ8* | 3.69E-03 | 1.22 | Potassium Inwardly-Rectifying Channel, Subfamily J, Member 8 |
| *HOOK1* | 1.32E-03 | 1.22 | Hook Microtubule-Tethering Protein 1 |
| *PIN1* | 3.82E-04 | 1.22 | Peptidylprolyl Cis/Trans Isomerase, Nima-Interacting 1 |
| *DUSP23* | 9.02E-04 | 1.22 | Dual Specificity Phosphatase 23 |
| *NUDT12* | 1.92E-03 | 1.22 | Nudix |
| *RAB3IP* | 6.37E-04 | 1.22 | Rab3A Interacting Protein |
| *MLH1* | 2.15E-03 | 1.23 | Mutl Homolog 1 |
| *SHARPIN* | 4.92E-03 | 1.23 | Shank-Associated Rh Domain Interactor |
| *UGT2B10* | 1.77E-04 | 1.23 | Udp Glucuronosyltransferase 2 Family, Polypeptide B10 |
| *LOC100506691* | 4.21E-03 | 1.23 | Predicted: Uncharacterized Loc100506691 |
| *FEZ2* | 7.87E-04 | 1.23 | Fasciculation And Elongation Protein Zeta 2 |
| *SPIN4* | 8.35E-04 | 1.23 | Spindlin Family, Member 4 |
| *UTRN* | 1.09E-03 | 1.23 | Utrophin |
| *RCCD1* | 2.26E-04 | 1.23 | Rcc1 Domain Containing 1 |
| *SIGLEC6* | 7.52E-04 | 1.23 | Sialic Acid Binding Ig-Like Lectin 6 |
| *CCNB1* | 4.90E-04 | 1.23 | Cyclin B1 |
| *SMAD6* | 2.99E-04 | 1.23 | Smad Family Member 6 |
| *LMF2* | 1.61E-04 | 1.23 | Lipase Maturation Factor 2 |
| *ZNF655* | 3.57E-04 | 1.23 | Zinc Finger Protein 655 |
| *MT1X* | 4.89E-06 | 1.23 | Metallothionein 1X |
| *NT5DC1* | 6.47E-04 | 1.23 | 5'-Nucleotidase Domain Containing 1 |
| *ATP2B4* | 3.20E-03 | 1.23 | Atpase, Ca++ Transporting, Plasma Membrane 4 |
| *GINS1* | 2.58E-04 | 1.23 | Gins Complex Subunit 1 |
| *LMO4* | 1.81E-04 | 1.23 | Lim Domain Only 4 |
| *SEPT9* | 2.10E-03 | 1.23 | Septin 9 |
| *CLASP2* | 5.84E-04 | 1.23 | Cytoplasmic Linker Associated Protein 2 |
| *IFNGR1* | 1.48E-04 | 1.23 | Interferon Gamma Receptor 1 |
| *RER1* | 1.08E-04 | 1.23 | Retention In Endoplasmic Reticulum Sorting Receptor 1 |
| *PHB2* | 3.50E-03 | 1.23 | Prohibitin 2 |
| *CAST* | 8.78E-04 | 1.24 | Calpastatin |
| *TIGD2* | 2.99E-03 | 1.24 | Tigger Transposable Element Derived 2 |
| *RER1* | 1.64E-05 | 1.24 | Retention In Endoplasmic Reticulum Sorting Receptor 1 |
| *APPBP2* | 4,32E-02 | 1.24 | Amyloid beta precursor protein |
| *ATP9A* | 5.96E-05 | 1.24 | Atpase, Class Ii, Type 9A |
| *ESRP2* | 3.29E-03 | 1.24 | Epithelial Splicing Regulatory Protein 2 |
| *PARP10* | 6.22E-04 | 1.24 | Poly |
| Uncharacterized | 5.41E-06 | 1.24 | Uncharacterized |
| *UFM1* | 5.14E-04 | 1.24 | Ubiquitin-Fold Modifier 1 |
| *STOML1* | 1.62E-03 | 1.24 | Stomatin |
| *ASPSCR1* | 9.34E-04 | 1.24 | Alveolar Soft Part Sarcoma Chromosome Region, Candidate 1 |
| *PRSS16* | 1.18E-03 | 1.24 | Protease, Serine, 16 |
| *GAL3ST1* | 9.47E-05 | 1.24 | Galactose-3-O-Sulfotransferase 1 |
| *CCNB2* | 1.48E-04 | 1.24 | Cyclin B2 |
| *MRPL23-AS1* | 3.90E-05 | 1.24 | Mrpl23 Antisense Rna 1 |
| *XLOC_l2_009136* | 2.58E-03 | 1.24 | Broad Institute Lincrna |
| *NUDT18* | 1.16E-04 | 1.24 | Nudix |
| *LINC00963* | 4.12E-05 | 1.24 | Long Intergenic Non-Protein Coding Rna 963 |
| *BIRC6* | 2.12E-04 | 1.24 | Baculoviral Iap Repeat Containing 6 |
| *CFLAR* | 2.21E-03 | 1.24 | Casp8 And Fadd-Like Apoptosis Regulator |
| *EPB41L1* | 2.31E-03 | 1.25 | Erythrocyte Membrane Protein Band 4.1-Like 1 |
| *NRXN3* | 3.73E-04 | 1.25 | Neurexin 3 |
| *ARFGEF2* | 7.37E-04 | 1.25 | Adp-Ribosylation Factor Guanine Nucleotide-Exchange Factor 2 |
| *LMO4* | 2.54E-03 | 1.25 | Lim Domain Only 4 |
| *LOC100288911* | 1.08E-03 | 1.25 | Uncharacterized Loc100288911 |
| *DKK1* | 8.01E-05 | 1.25 | Dickkopf Wnt Signaling Pathway Inhibitor 1 |
| *DICER1* | 1.43E-05 | 1.25 | Dicer 1, Ribonuclease Type Iii |
| *NCAPD2* | 1.24E-04 | 1.25 | Non-Smc Condensin I Complex, Subunit D2 |
| *TRPT1* | 3.50E-03 | 1.25 | Trna Phosphotransferase 1 |
| *ASPM* | 4.83E-04 | 1.25 | Asp |
| *CXXC4* | 1.20E-05 | 1.25 | Cxxc Finger Protein 4 |
| *CHST9* | 4.67E-03 | 1.25 | Carbohydrate |
| *CENPF* | 7.99E-05 | 1.25 | Centromere Protein F, 350/400Kda |
| *GLDC* | 1.14E-06 | 1.25 | Glycine Dehydrogenase |
| *SLC39A5* | 5.73E-04 | 1.25 | Solute Carrier Family 39 |
| *CAPN2* | 4.58E-04 | 1.25 | Calpain 2, |
| *ARHGAP28* | 8.89E-04 | 1.25 | Rho Gtpase Activating Protein 28 |
| *LEAP2* | 1.20E-03 | 1.25 | Liver Expressed Antimicrobial Peptide 2 |
| *TMEM106C* | 4.10E-04 | 1.25 | Transmembrane Protein 106C |
| *ASB9P1* | 7.05E-04 | 1.25 | Ankyrin Repeat And Socs Box Containing 9 Pseudogene 1 |
| *ASF1B* | 2.49E-05 | 1.25 | Anti-Silencing Function 1B Histone Chaperone |
| *LAMC1* | 2.45E-03 | 1.26 | Laminin, Gamma 1 |
| *KREMEN2* | 8.62E-04 | 1.26 | Kringle Containing Transmembrane Protein 2 |
| *XLOC_005081* | 4.71E-03 | 1.26 | Broad Institute Lincrna |
| *PCSK6* | 4.46E-05 | 1.26 | Proprotein Convertase Subtilisin/Kexin Type 6 |
| *NKD2* | 1.69E-03 | 1.26 | Naked Cuticle Homolog 2 |
| *ETAA1* | 4.86E-04 | 1.26 | Ewing Tumor-Associated Antigen 1 |
| *TCF4* | 4.60E-04 | 1.26 | Transcription Factor 4 |
| *PGLS* | 2.19E-07 | 1.26 | 6-Phosphogluconolactonase |
| *TRIP13* | 7.45E-04 | 1.26 | Thyroid Hormone Receptor Interactor 13 |
| *DCXR* | 1.86E-03 | 1.26 | Dicarbonyl/L-Xylulose Reductase |
| *MACROD1* | 1.87E-04 | 1.26 | Macro Domain Containing 1 |
| *FAM83D* | 4.48E-04 | 1.26 | Family With Sequence Similarity 83, Member D |
| *LIPT2* | 3.47E-05 | 1.26 | Lipoyl |
| *VPS13C* | 1.77E-03 | 1.26 | Vacuolar Protein Sorting 13 Homolog C |
| *EMILIN2* | 3.82E-05 | 1.26 | Elastin Microfibril Interfacer 2 |
| *PCNA* | 9.53E-05 | 1.26 | Proliferating Cell Nuclear Antigen |
| *DPYSL3* | 1.29E-03 | 1.26 | Dihydropyrimidinase-Like 3 |
| *PBX3* | 2.37E-03 | 1.26 | Pre-B-Cell Leukemia Homeobox 3 |
| *PCNA* | 1.05E-04 | 1.26 | Proliferating Cell Nuclear Antigen |
| *PCYOX1L* | 2.30E-03 | 1.26 | Prenylcysteine Oxidase 1 Like |
| *CD44* | 1.34E-03 | 1.26 | Cd44 Molecule |
| Uncharacterized | 1.52E-03 | 1.26 | Uncharacterized |
| *CD24* | 8.69E-04 | 1.26 | Cd24 Molecule |
| *ALDH4A1* | 4.78E-05 | 1.27 | Aldehyde Dehydrogenase 4 Family, Member A1 |
| *PCNA* | 3.13E-04 | 1.27 | Proliferating Cell Nuclear Antigen |
| *IGFLR1* | 9.06E-04 | 1.27 | Igf-Like Family Receptor 1 |
| *CENPU* | 3.50E-03 | 1.27 | Centromere Protein U |
| *QPCTL* | 5.33E-04 | 1.27 | Glutaminyl-Peptide Cyclotransferase-Like |
| *IGF1R* | 3.25E-03 | 1.27 | Insulin-Like Growth Factor 1 Receptor |
| *SNX14* | 2.06E-04 | 1.27 | Sorting Nexin 14 |
| *PDE6A* | 1.14E-05 | 1.27 | Phosphodiesterase 6A, Cgmp-Specific, Rod, Alpha |
| *NDUFA11* | 5.74E-04 | 1.27 | Nadh Dehydrogenase |
| *AGL* | 1.42E-03 | 1.27 | Amylo-Alpha-1, 6-Glucosidase, 4-Alpha-Glucanotransferase |
| *SLC22A23* | 3.88E-05 | 1.27 | Solute Carrier Family 22, Member 23 |
| *TTC7B* | 8.53E-04 | 1.27 | Tetratricopeptide Repeat Domain 7B |
| *PMP22* | 8.45E-05 | 1.27 | Peripheral Myelin Protein 22 |
| *PAFAH1B1* | 2.43E-06 | 1.27 | Platelet-Activating Factor Acetylhydrolase 1B, Regulatory Subunit 1 |
| *TBCD* | 2.17E-03 | 1.27 | Tubulin Folding Cofactor D |
| *MCM7* | 3.93E-04 | 1.27 | Minichromosome Maintenance Complex Component 7 |
| *TFEC* | 7.54E-05 | 1.27 | Transcription Factor Ec |
| *ADRBK2* | 8.96E-04 | 1.27 | Adrenergic, Beta, Receptor Kinase 2 |
| *ARHGEF10* | 5.72E-06 | 1.28 | Rho Guanine Nucleotide Exchange Factor |
| *LINC00963* | 2.95E-04 | 1.28 | Long Intergenic Non-Protein Coding Rna 963 |
| *TPM1* | 1.45E-03 | 1.28 | Tropomyosin 1 |
| *HMGB1* | 9.10E-04 | 1.28 | High Mobility Group Box 1 |
| *NFATC2IP* | 1.84E-03 | 1.28 | Nuclear Factor Of Activated T-Cells, Cytoplasmic, Calcineurin-Dependent 2 Interacting Protein |
| *ZNF22* | 7.43E-06 | 1.28 | Zinc Finger Protein 22 |
| *PRSS23* | 4.65E-03 | 1.28 | Protease, Serine, 23 |
| *PDGFRA* | 1.61E-03 | 1.28 | Platelet-Derived Growth Factor Receptor, Alpha Polypeptide |
| *GLUD2* | 2.45E-03 | 1.28 | Glutamate Dehydrogenase 2 |
| *CTDSP1* | 2.57E-05 | 1.28 | Ctd |
| *ABCD3* | 1.35E-03 | 1.28 | Atp-Binding Cassette, Sub-Family D |
| *MORC4* | 1.25E-04 | 1.28 | Morc Family Cw-Type Zinc Finger 4 |
| *MYH9* | 8.96E-05 | 1.28 | Myosin, Heavy Chain 9, Non-Muscle |
| *MCAM* | 1.07E-03 | 1.28 | Melanoma Cell Adhesion Molecule |
| *TRIM5* | 9.43E-04 | 1.28 | Tripartite Motif Containing 5 |
| *COG6* | 5.12E-04 | 1.28 | Component Of Oligomeric Golgi Complex 6 |
| *ACTN4* | 1.17E-03 | 1.28 | Actinin, Alpha 4 |
| *NAV2* | 2.88E-04 | 1.28 | Neuron Navigator 2 |
| *STK31* | 1.12E-04 | 1.28 | Serine/Threonine Kinase 31 |
| *PTPRS* | 3.76E-06 | 1.28 | Protein Tyrosine Phosphatase, Receptor Type, S |
| *XLOC_010032* | 1.79E-04 | 1.28 | Broad Institute Lincrna |
| *DAZAP1* | 1.47E-04 | 1.29 | Daz Associated Protein 1 |
| *TEX30* | 2.56E-04 | 1.29 | Testis Expressed 30 |
| *NOTCH3* | 8.69E-08 | 1.29 | Notch 3 |
| *CDKN3* | 1.98E-04 | 1.29 | Cyclin-Dependent Kinase Inhibitor 3 |
| *KRT23* | 4.98E-04 | 1.29 | Keratin 23 |
| *NDC80* | 1.53E-07 | 1.29 | Ndc80 Kinetochore Complex Component |
| *CEP152* | 6.42E-04 | 1.29 | Cdna Clone Image:5296881. |
| *NHLRC3* | 2.13E-03 | 1.29 | Nhl Repeat Containing 3 |
| *KAT6A* | 2.84E-03 | 1.29 | K |
| *TMTC4* | 4.88E-04 | 1.29 | Transmembrane And Tetratricopeptide Repeat Containing 4 |
| *PLD2* | 4.30E-03 | 1.29 | Phospholipase D2 |
| *TSTD3* | 3.87E-04 | 1.29 | Thiosulfate Sulfurtransferase |
| *ATP9A* | 5.13E-04 | 1.29 | Atpase, Class Ii, Type 9A |
| *IVD* | 9.55E-05 | 1.29 | Isovaleryl-Coa Dehydrogenase |
| *GTSE1* | 1.43E-04 | 1.29 | G-2 And S-Phase Expressed 1 |
| *CORO1B* | 1.96E-03 | 1.30 | Coronin, Actin Binding Protein, 1B |
| *NCAPD2* | 4.20E-05 | 1.30 | Non-Smc Condensin I Complex, Subunit D2 |
| *NUDT8* | 8.36E-04 | 1.30 | Nudix |
| *KDM2B* | 1.58E-03 | 1.30 | Lysine |
| *SPDL1* | 1.73E-03 | 1.30 | Spindle Apparatus Coiled-Coil Protein 1 |
| *SMIM19* | 4.28E-03 | 1.30 | Small Integral Membrane Protein 19 |
| *MSRB2* | 5.13E-04 | 1.30 | Methionine Sulfoxide Reductase B2 |
| *C8orf37* | 3.34E-03 | 1.30 | Chromosome 8 Open Reading Frame 37 |
| *TSPAN18* | 1.54E-04 | 1.30 | Tetraspanin 18 |
| *APOBEC2* | 1.18E-05 | 1.30 | Apolipoprotein B Mrna Editing Enzyme, Catalytic Polypeptide-Like 2 |
| *DMTN* | 1.35E-05 | 1.30 | Dematin Actin Binding Protein |
| *GRIN2C* | 3.13E-03 | 1.30 | Glutamate Receptor, Ionotropic, N-Methyl D-Aspartate 2C |
| *XLOC_l2_003626* | 1.22E-03 | 1.30 | Broad Institute Lincrna |
| *ARL6IP4* | 3.25E-06 | 1.30 | Adp-Ribosylation Factor-Like 6 Interacting Protein 4 |
| *DNAJB5* | 4.08E-03 | 1.30 | Dnaj |
| *BORA* | 1.62E-03 | 1.30 | Bora, Aurora Kinase A Activator |
| *FBXO5* | 9.19E-04 | 1.30 | F-Box Protein 5 |
| *RPA3* | 4.42E-04 | 1.30 | Replication Protein A3, 14Kda |
| *PRKCA* | 5.27E-05 | 1.30 | Protein Kinase C, Alpha |
| *EHD2* | 1.99E-08 | 1.30 | Eh-Domain Containing 2 |
| *PRKCDBP* | 1.56E-04 | 1.31 | Protein Kinase C, Delta Binding Protein |
| *COMMD3* | 5.56E-05 | 1.31 | Comm Domain Containing 3 |
| *LOXL3* | 2.87E-04 | 1.31 | Lysyl Oxidase-Like 3 |
| *KIF20A* | 7.06E-04 | 1.31 | Kinesin Family Member 20A |
| *PRTFDC1* | 2.85E-03 | 1.31 | Phosphoribosyl Transferase Domain Containing 1 |
| *H2AFJ* | 4.66E-06 | 1.31 | H2A Histone Family, Member J |
| *DAK* | 6.34E-04 | 1.31 | Dihydroxyacetone Kinase 2 Homolog |
| *SPATA5L1* | 2.36E-03 | 1.31 | Spermatogenesis Associated 5-Like 1 |
| *CDCA7* | 1.61E-03 | 1.31 | Cell Division Cycle Associated 7 |
| *CCDC121* | 8.31E-04 | 1.31 | Coiled-Coil Domain Containing 121 |
| *SIDT2* | 2.82E-04 | 1.31 | Sid1 Transmembrane Family, Member 2 |
| *LOC729970* | 1.20E-03 | 1.31 | Hcg2028352-Like |
| *EEA1* | 9.27E-04 | 1.31 | Early Endosome Antigen 1 |
| *CIT* | 4.78E-05 | 1.31 | Citron Rho-Interacting Serine/Threonine Kinase |
| *AMACR* | 7.29E-05 | 1.31 | Alpha-Methylacyl-Coa Racemase |
| *TUBB2B* | 8.50E-05 | 1.32 | Tubulin, Beta 2B Class Iib |
| *C16orf58* | 7.60E-04 | 1.32 | Chromosome 16 Open Reading Frame 58 |
| *VIL1* | 3.68E-03 | 1.32 | Villin 1 |
| *WNT10A* | 4.22E-03 | 1.32 | Wingless-Type Mmtv Integration Site Family, Member 10A |
| *CEP55* | 3.79E-03 | 1.32 | Centrosomal Protein 55Kda |
| *BMP7* | 2.02E-04 | 1.32 | Bone Morphogenetic Protein 7 |
| *CSRP2* | 4.08E-04 | 1.32 | Cysteine And Glycine-Rich Protein 2 |
| *RMI2* | 2.25E-04 | 1.32 | Recq Mediated Genome Instability 2 |
| *SREBF1* | 2.99E-04 | 1.32 | Sterol Regulatory Element Binding Transcription Factor 1 |
| *SLC16A3* | 3.50E-03 | 1.32 | Solute Carrier Family 16 |
| *GPCPD1* | 1.67E-03 | 1.32 | Glycerophosphocholine Phosphodiesterase Gde1 Homolog |
| *TEX40* | 3.56E-03 | 1.32 | Testis Expressed 40 |
| *ADD3* | 5.41E-04 | 1.32 | Adducin 3 |
| *HES6* | 3.82E-03 | 1.32 | Hes Family Bhlh Transcription Factor 6 |
| *ASPM* | 4.64E-05 | 1.32 | Asp |
| *ADAM19* | 7.89E-04 | 1.32 | Adam Metallopeptidase Domain 19 |
| *NIPAL4* | 1.77E-03 | 1.32 | Nipa-Like Domain Containing 4 |
| *PPP3CB* | 1.24E-03 | 1.32 | Protein Phosphatase 3, Catalytic Subunit, Beta Isozyme |
| *FAM111A* | 9.28E-04 | 1.32 | Family With Sequence Similarity 111, Member A |
| *TNNC1* | 4.08E-04 | 1.32 | Troponin C Type 1 |
| *ZNF441* | 3.91E-03 | 1.32 | Zinc Finger Protein 441 |
| *NIPSNAP3A* | 9.04E-04 | 1.32 | Nipsnap Homolog 3A |
| *JADE1* | 6.09E-05 | 1.33 | Jade Family Phd Finger 1 |
| *HYI* | 2.36E-03 | 1.33 | Hydroxypyruvate Isomerase |
| *ANGPT2* | 3.91E-03 | 1.33 | Angiopoietin 2 |
| *SIDT1* | 2.81E-04 | 1.33 | Sid1 Transmembrane Family, Member 1 |
| *NETO2* | 2.37E-03 | 1.33 | Neuropilin |
| *C1RL* | 1.01E-04 | 1.33 | Complement Component 1, R Subcomponent-Like |
| *MDP1* | 1.54E-03 | 1.33 | Magnesium-Dependent Phosphatase 1 |
| *SDSL* | 2.18E-04 | 1.33 | Serine Dehydratase-Like |
| *SBF2* | 4.48E-04 | 1.33 | Set Binding Factor 2 |
| *C11orf80* | 2.58E-04 | 1.33 | Chromosome 11 Open Reading Frame 80 |
| *ATP11A* | 8.60E-04 | 1.33 | Atpase, Class Vi, Type 11A |
| *NICN1* | 1.16E-03 | 1.33 | Nicolin 1 |
| *CDC42BPB* | 6.64E-04 | 1.33 | Cdc42 Binding Protein Kinase Beta |
| *NRXN3* | 1.45E-04 | 1.34 | Neurexin 3 |
| *PDGFRL* | 2.51E-03 | 1.34 | Platelet-Derived Growth Factor Receptor-Like |
| *CHST15* | 1.81E-04 | 1.34 | Carbohydrate |
| *PPP1R9A* | 2.17E-05 | 1.34 | Protein Phosphatase 1, Regulatory Subunit 9A |
| *KAZALD1* | 6.75E-04 | 1.34 | Kazal-Type Serine Peptidase Inhibitor Domain 1 |
| *TPK1* | 1.34E-04 | 1.34 | Thiamin Pyrophosphokinase 1 |
| *KIAA0196* | 8.25E-04 | 1.34 | Kiaa0196 |
| *CCND2* | 1.35E-04 | 1.34 | Cyclin D2 |
| *SPAG16* | 9.68E-04 | 1.34 | Sperm Associated Antigen 16 |
| *YBX2* | 3.46E-04 | 1.34 | Y Box Binding Protein 2 |
| *FAM72A* | 1.41E-03 | 1.34 | Family With Sequence Similarity 72, Member A |
| *POU4F1* | 2.10E-03 | 1.34 | Pou Class 4 Homeobox 1 |
| *Hsj1175I6* | 3.11E-03 | 1.34 | Hsj1175I6 Ras And Rab Interactor 2 |
| *KIAA0586* | 1.22E-03 | 1.35 | Kiaa0586 |
| *HMHA1* | 2.43E-03 | 1.35 | Histocompatibility |
| *ISOC2* | 3.16E-03 | 1.35 | Isochorismatase Domain Containing 2 |
| *PODXL* | 6.10E-05 | 1.35 | Podocalyxin-Like |
| *ESRP1* | 2.14E-03 | 1.35 | Epithelial Splicing Regulatory Protein 1 |
| *ST6GALNAC3* | 6.85E-05 | 1.35 | St6 |
| *PRR5-ARHGAP8* | 1.56E-03 | 1.35 | Prr5-Arhgap8 Readthrough |
| *ANKRD20A5P* | 2.48E-03 | 1.35 | Ankyrin Repeat Domain 20 Family, Member A5, Pseudogene |
| *TSPAN4* | 3.55E-03 | 1.35 | Tetraspanin 4 |
| *MPHOSPH9* | 2.25E-03 | 1.35 | M-Phase Phosphoprotein 9 |
| *CACNG6* | 2.39E-03 | 1.35 | Calcium Channel, Voltage-Dependent, Gamma Subunit 6 |
| *ZNRF1* | 3.46E-04 | 1.35 | Zinc And Ring Finger 1, E3 Ubiquitin Protein Ligase |
| *RHOBTB1* | 1.74E-07 | 1.35 | Rho-Related Btb Domain Containing 1 |
| *NKD1* | 9.03E-06 | 1.35 | Naked Cuticle Homolog 1 |
| *VLDLR* | 2.11E-04 | 1.36 | Very Low Density Lipoprotein Receptor |
| *PCED1B* | 1.57E-03 | 1.36 | Pc-Esterase Domain Containing 1B |
| *AMACR* | 6.03E-05 | 1.36 | Alpha-Methylacyl-Coa Racemase |
| *PDE7A* | 3.24E-03 | 1.36 | Camp-Specific Cyclic Nucleotide Phosphodiesterase Pde7A3 Mrna, Complete Cds. |
| *MKI67* | 2.38E-03 | 1.36 | Marker Of Proliferation Ki-67 |
| *NDUFA7* | 4.12E-04 | 1.36 | Nadh Dehydrogenase |
| *PPP3CB* | 5.70E-05 | 1.36 | Protein Phosphatase 3, Catalytic Subunit, Beta Isozyme |
| *CDCA4* | 7.02E-04 | 1.36 | Cell Division Cycle Associated 4 |
| *IFT172* | 9.89E-04 | 1.36 | Intraflagellar Transport 172 Homolog |
| *FASTK* | 4.29E-03 | 1.36 | Fas-Activated Serine/Threonine Kinase |
| *TMX4* | 1.40E-03 | 1.36 | Thioredoxin-Related Transmembrane Protein 4 |
| *HMGA2* | 5.31E-05 | 1.36 | High Mobility Group At-Hook 2 |
| *LEPREL4* | 9.08E-04 | 1.36 | Leprecan-Like 4 |
| *FREM2* | 2.59E-04 | 1.36 | Fras1 Related Extracellular Matrix Protein 2 |
| *SYNPO2L* | 2.84E-03 | 1.37 | Synaptopodin 2-Like |
| *FAM169A* | 3.17E-03 | 1.37 | Family With Sequence Similarity 169, Member A |
| *ARHGAP26* | 1.08E-05 | 1.37 | Rho Gtpase Activating Protein 26 |
| *GOLGA3* | 2.90E-03 | 1.37 | Golgin A3 |
| *MYCN* | 1.25E-05 | 1.37 | V-Myc Avian Myelocytomatosis Viral Oncogene Neuroblastoma Derived Homolog |
| *HPGD* | 8.98E-06 | 1.37 | Hydroxyprostaglandin Dehydrogenase 15- |
| *JUND* | 4.94E-03 | 1.37 | Jun D Proto-Oncogene |
| *C11orf31* | 1.63E-03 | 1.37 | Chromosome 11 Open Reading Frame 31 |
| *LOC202181* | 2.76E-03 | 1.37 | Sumo-Interacting Motifs Containing 1 Pseudogene |
| *LAMA1* | 3.59E-06 | 1.37 | Laminin, Alpha 1 |
| *TPM1* | 7.29E-04 | 1.37 | Tropomyosin 1 |
| *PPOX* | 2.38E-03 | 1.37 | Protoporphyrinogen Oxidase |
| *TSPAN5* | 2.98E-04 | 1.38 | Tetraspanin 5 |
| *TPM1* | 4.68E-03 | 1.38 | Tropomyosin 1 |
| *RTKN2* | 5.69E-05 | 1.38 | Rhotekin 2 |
| *KIF12* | 4.80E-04 | 1.38 | Kinesin Family Member 12 |
| *VLDLR-AS1* | 2.15E-03 | 1.38 | Vldlr Antisense Rna 1 |
| *SERPINB1* | 5.30E-06 | 1.38 | Serpin Peptidase Inhibitor, Clade B |
| *PACSIN1* | 2.12E-04 | 1.38 | Protein Kinase C And Casein Kinase Substrate In Neurons 1 |
| *IGDCC3* | 2.59E-05 | 1.38 | Immunoglobulin Superfamily, Dcc Subclass, Member 3 |
| *DHFR* | 4.62E-03 | 1.38 | Dihydrofolate Reductase |
| *TRIOBP* | 2.35E-03 | 1.38 | Trio And F-Actin Binding Protein |
| *HAGH* | 1.41E-03 | 1.38 | Hydroxyacylglutathione Hydrolase |
| *FGFR2* | 1.59E-05 | 1.38 | Fibroblast Growth Factor Receptor 2 |
| *DEPDC1* | 1.24E-03 | 1.38 | Dep Domain Containing 1 |
| *SYNE2* | 6.23E-05 | 1.39 | Spectrin Repeat Containing, Nuclear Envelope 2 |
| *ARL15* | 3.07E-03 | 1.39 | Adp-Ribosylation Factor-Like 15 |
| *GPX1* | 4.13E-04 | 1.39 | Glutathione Peroxidase 1 |
| *ENTPD4* | 7.16E-04 | 1.39 | Ectonucleoside Triphosphate Diphosphohydrolase 4 |
| *SPG11* | 4.55E-03 | 1.39 | Spastic Paraplegia 11 |
| *SULF2* | 4.09E-03 | 1.39 | Sulfatase 2 |
| *CDC7* | 3.66E-04 | 1.39 | Cell Division Cycle 7 |
| *KANK4* | 8.45E-04 | 1.39 | Kn Motif And Ankyrin Repeat Domains 4 |
| *ODF3B* | 4.28E-03 | 1.39 | Outer Dense Fiber Of Sperm Tails 3B |
| *CLEC16A* | 1.23E-04 | 1.39 | C-Type Lectin Domain Family 16, Member A |
| *DNAJB5* | 4.37E-03 | 1.39 | Dnaj |
| *PSMG4* | 2.28E-05 | 1.39 | Proteasome |
| *CBS* | 1.69E-05 | 1.39 | Cystathionine-Beta-Synthase |
| *ACTG2* | 2.17E-03 | 1.39 | Actin, Gamma 2, Smooth Muscle, Enteric |
| *ARHGEF17* | 1.67E-04 | 1.39 | Rho Guanine Nucleotide Exchange Factor |
| *CENPK* | 3.88E-03 | 1.40 | Centromere Protein K |
| *CELSR1* | 1.14E-03 | 1.40 | Cadherin, Egf Lag Seven-Pass G-Type Receptor 1 |
| *C11orf31* | 7.79E-04 | 1.40 | Chromosome 11 Open Reading Frame 31 |
| *LOC101927016* | 1.88E-03 | 1.40 | Uncharacterized Protein |
| *SMYD2* | 2.92E-07 | 1.40 | Set And Mynd Domain Containing 2 |
| *HULC* | 1.58E-04 | 1.40 | Hepatocellular Carcinoma Up-Regulated Long Non-Coding Rna |
| *ARHGEF40* | 3.33E-05 | 1.40 | Rho Guanine Nucleotide Exchange Factor |
| *ALKBH6* | 1.39E-03 | 1.40 | Alkb, Alkylation Repair Homolog 6 |
| *CRNDE* | 1.92E-03 | 1.40 | Colorectal Neoplasia Differentially Expressed |
| *NSMCE4A* | 3.56E-05 | 1.40 | Non-Smc Element 4 Homolog A |
| *ING5* | 3.57E-03 | 1.40 | Inhibitor Of Growth Family, Member 5 |
| *ATP2A2* | 1.03E-05 | 1.41 | Atpase, Ca++ Transporting, Cardiac Muscle, Slow Twitch 2 |
| *KITLG* | 5.84E-04 | 1.41 | Kit Ligand |
| *VLDLR-AS1* | 1.12E-03 | 1.41 | Vldlr Antisense Rna 1 |
| *ME3* | 2.33E-03 | 1.41 | Malic Enzyme 3, Nadp |
| *DISC1* | 1.72E-03 | 1.41 | Disrupted In Schizophrenia 1 |
| *HGSNAT* | 9.30E-06 | 1.41 | Heparan-Alpha-Glucosaminide N-Acetyltransferase |
| *KRT23* | 3.15E-04 | 1.41 | Keratin 23 |
| *GABRA2* | 4.37E-04 | 1.41 | Gamma-Aminobutyric Acid |
| *ENTPD4* | 2.85E-03 | 1.41 | Ectonucleoside Triphosphate Diphosphohydrolase 4 |
| *MKI67* | 2.88E-03 | 1.41 | Marker Of Proliferation Ki-67 |
| *DOCK8* | 8.76E-04 | 1.41 | Dedicator Of Cytokinesis 8 |
| *MT1E* | 1.49E-05 | 1.41 | Metallothionein 1E |
| *RIBC2* | 1.85E-03 | 1.41 | Rib43A Domain With Coiled-Coils 2 |
| *TRIM65* | 3.83E-03 | 1.41 | Tripartite Motif Containing 65 |
| *ATP2C2* | 2.02E-04 | 1.41 | Atpase, Ca++ Transporting, Type 2C, Member 2 |
| *KANK4* | 3.85E-04 | 1.41 | Kn Motif And Ankyrin Repeat Domains 4 |
| *C15orf38* | 3.97E-04 | 1.41 | Chromosome 15 Open Reading Frame 38 |
| *NSMCE4A* | 3.57E-05 | 1.41 | Non-Smc Element 4 Homolog A |
| *Zswm1* | 3.16E-03 | 1.41 | Zswm1_Human |
| *SPRR3* | 2.66E-03 | 1.41 | Small Proline-Rich Protein 3 |
| *SHANK2* | 1.83E-04 | 1.41 | Sh3 And Multiple Ankyrin Repeat Domains 2 |
| *PYCR1* | 4.64E-03 | 1.42 | Pyrroline-5-Carboxylate Reductase 1 |
| *CLN6* | 4.60E-04 | 1.42 | Ceroid-Lipofuscinosis, Neuronal 6, Late Infantile, Variant |
| *SLC4A8* | 2.80E-04 | 1.42 | Solute Carrier Family 4, Sodium Bicarbonate Cotransporter, Member 8 |
| *PXMP2* | 1.93E-03 | 1.42 | Peroxisomal Membrane Protein 2, 22Kda |
| *HMGB2* | 3.85E-03 | 1.42 | High Mobility Group Box 2 |
| *CRNDE* | 2.07E-03 | 1.42 | Colorectal Neoplasia Differentially Expressed |
| *E2F7* | 4.19E-03 | 1.42 | E2F Transcription Factor 7 |
| *H1FX* | 2.08E-05 | 1.42 | H1 Histone Family, Member X |
| *CSGALNACT1* | 3.71E-03 | 1.42 | Chondroitin Sulfate N-Acetylgalactosaminyltransferase 1 |
| *SYNE1* | 7.51E-06 | 1.42 | Spectrin Repeat Containing, Nuclear Envelope 1 |
| *CD14* | 1.58E-03 | 1.43 | Cd14 Molecule |
| *C16orf95* | 3.33E-03 | 1.43 | Chromosome 16 Open Reading Frame 95 |
| *AKTIP* | 1.87E-03 | 1.43 | Akt Interacting Protein |
| *FAM115A* | 4.53E-04 | 1.43 | Family With Sequence Similarity 115, Member A |
| *ERBB2* | 1.88E-03 | 1.43 | V-Erb-B2 Avian Erythroblastic Leukemia Viral Oncogene Homolog 2 |
| *TIA1* | 2.76E-03 | 1.43 | Tia1 Cytotoxic Granule-Associated Rna Binding Protein |
| *CADM1* | 2.56E-05 | 1.43 | Cell Adhesion Molecule 1 |
| *DOCK11* | 1.15E-03 | 1.43 | Dedicator Of Cytokinesis 11 |
| *NUF2* | 3.51E-03 | 1.43 | Nuf2, Ndc80 Kinetochore Complex Component |
| *C11orf70* | 4.78E-03 | 1.43 | Chromosome 11 Open Reading Frame 70 |
| *PRTFDC1* | 1.45E-03 | 1.43 | Phosphoribosyl Transferase Domain Containing 1 |
| *SMIM2-AS1* | 1.57E-04 | 1.44 | Smim2 Antisense Rna 1 |
| *WDR54* | 3.26E-03 | 1.44 | Wd Repeat Domain 54 |
| *MYLK* | 2.55E-05 | 1.44 | Myosin Light Chain Kinase |
| *REEP1* | 1.60E-04 | 1.44 | Receptor Accessory Protein 1 |
| *CADM1* | 2.52E-04 | 1.44 | Cell Adhesion Molecule 1 |
| *CREG2* | 1.31E-03 | 1.44 | Cellular Repressor Of E1A-Stimulated Genes 2 |
| *MTSS1* | 1.54E-05 | 1.44 | Metastasis Suppressor 1 |
| *SLCO2B1* | 5.75E-07 | 1.44 | Solute Carrier Organic Anion Transporter Family, Member 2B1 |
| *MAP6* | 2.32E-04 | 1.44 | Microtubule-Associated Protein 6 |
| *DOCK9* | 4.30E-04 | 1.44 | Dedicator Of Cytokinesis 9 |
| *MT1E* | 4.10E-08 | 1.44 | Metallothionein 1E |
| *MANEA* | 1.30E-03 | 1.44 | Mannosidase, Endo-Alpha |
| *TG* | 1.15E-03 | 1.45 | Thyroglobulin |
| *DCDC2* | 9.22E-04 | 1.45 | Doublecortin Domain Containing 2 |
| *NPPB* | 1.53E-04 | 1.45 | Natriuretic Peptide B |
| *CDC45* | 1.16E-04 | 1.45 | Cell Division Cycle 45 |
| *CRNDE* | 1.23E-03 | 1.45 | Colorectal Neoplasia Differentially Expressed |
| *FAM209A* | 2.22E-03 | 1.45 | Family With Sequence Similarity 209, Member A |
| *SERPINF1* | 3.85E-03 | 1.45 | Serpin Peptidase Inhibitor, Clade F |
| *EPSTI1* | 6.01E-04 | 1.46 | Epithelial Stromal Interaction 1 |
| *FOXO6* | 3.08E-03 | 1.46 | Forkhead Box O6 |
| *PRICKLE2* | 2.65E-03 | 1.46 | Prickle Homolog 2 |
| *HGSNAT* | 1.97E-04 | 1.46 | Heparan-Alpha-Glucosaminide N-Acetyltransferase |
| *DAK* | 3.07E-03 | 1.46 | Dihydroxyacetone Kinase 2 Homolog |
| *ACTA1* | 2.20E-05 | 1.47 | Actin, Alpha 1, Skeletal Muscle |
| *FBXL2* | 2.69E-03 | 1.47 | F-Box And Leucine-Rich Repeat Protein 2 |
| *DNMT3B* | 5.89E-04 | 1.47 | Dna |
| *VWA8* | 2.54E-04 | 1.47 | Von Willebrand Factor A Domain Containing 8 |
| *C8orf31* | 2.22E-04 | 1.47 | Chromosome 8 Open Reading Frame 31 |
| *C1orf105* | 2.74E-03 | 1.47 | Chromosome 1 Open Reading Frame 105 |
| *FAM173A* | 2.19E-04 | 1.47 | Family With Sequence Similarity 173, Member A |
| *RNF183* | 2.38E-04 | 1.47 | Ring Finger Protein 183 |
| *LINC00313* | 5.98E-04 | 1.47 | Long Intergenic Non-Protein Coding Rna 313 |
| *CASC10* | 5.01E-04 | 1.48 | Cancer Susceptibility Candidate 10 |
| *XLOC_l2_008043* | 9.75E-04 | 1.48 | Broad Institute Lincrna |
| *NRP1* | 1.44E-03 | 1.48 | Neuropilin 1 |
| *LRLE1* | 1.63E-03 | 1.48 | Liver-Related Low Express Protein 1 |
| *CENPE* | 3.62E-06 | 1.48 | Centromere Protein E, 312Kda |
| *FRAS1* | 1.20E-04 | 1.48 | Fraser Syndrome 1 |
| *TSHZ1* | 4.18E-03 | 1.49 | Teashirt Zinc Finger Homeobox 1 |
| *CFD* | 7.49E-04 | 1.49 | Complement Factor D |
| *RBFOX3* | 1.10E-04 | 1.49 | Rna Binding Protein, Fox-1 Homolog |
| *AKTIP* | 4.36E-03 | 1.49 | Akt Interacting Protein |
| *CRNDE* | 3.57E-03 | 1.49 | Colorectal Neoplasia Differentially Expressed |
| *PMPCA* | 2.59E-03 | 1.50 | Peptidase |
| *XLOC_007855* | 8.31E-04 | 1.50 | Broad Institute Lincrna |
| *GPR180* | 1.22E-04 | 1.50 | G Protein-Coupled Receptor 180 |
| *SOX2* | 2.59E-05 | 1.50 | Sry |
| *VPS13B* | 2.49E-03 | 1.50 | Vacuolar Protein Sorting 13 Homolog B |
| *ST6GALNAC3* | 6.97E-05 | 1.50 | St6 |
| *HSD17B8* | 2.01E-04 | 1.50 | Hydroxysteroid |
| *LIN7A* | 8.96E-04 | 1.50 | Lin-7 Homolog A |
| *LIMS2* | 1.28E-04 | 1.50 | Lim And Senescent Cell Antigen-Like Domains 2 |
| *GSTK1* | 1.34E-03 | 1.50 | Glutathione S-Transferase Kappa 1 |
| *FRY* | 3.33E-05 | 1.51 | Furry Homolog |
| *LOC100506178* | 2.47E-03 | 1.51 | Uncharacterized Loc100506178 |
| *UTRN* | 2.87E-03 | 1.51 | Utrophin |
| *C21orf2* | 2.68E-03 | 1.51 | Chromosome 21 Open Reading Frame 2 |
| *ME3* | 7.43E-05 | 1.51 | Malic Enzyme 3, Nadp |
| *MT1X* | 6.94E-05 | 1.51 | Metallothionein 1X |
| *XLOC_009837* | 2.37E-03 | 1.51 | Broad Institute Lincrna |
| *IKZF2* | 2.98E-04 | 1.51 | Ikaros Family Zinc Finger 2 |
| *C3orf70* | 2.79E-04 | 1.52 | Chromosome 3 Open Reading Frame 70 |
| *SOSTDC1* | 7.88E-04 | 1.52 | Sclerostin Domain Containing 1 |
| *KCNJ16* | 2.17E-05 | 1.52 | Potassium Inwardly-Rectifying Channel, Subfamily J, Member 16 |
| *AKTIP* | 1.14E-04 | 1.52 | Akt Interacting Protein |
| *LGALS1* | 1.57E-03 | 1.53 | Lectin, Galactoside-Binding, Soluble, 1 |
| *ISPD* | 1.11E-03 | 1.53 | Isoprenoid Synthase Domain Containing |
| *PI4K2B* | 5.84E-04 | 1.53 | Phosphatidylinositol 4-Kinase Type 2 Beta |
| *MMP24* | 5.98E-04 | 1.53 | Matrix Metallopeptidase 24 |
| *DEFB1* | 1.17E-03 | 1.53 | Defensin, Beta 1 |
| *SLC16A9* | 4.41E-04 | 1.53 | Solute Carrier Family 16, Member 9 |
| *SKIDA1* | 3.73E-04 | 1.53 | Ski/Dach Domain Containing 1 |
| *MT1M* | 4.80E-07 | 1.53 | Metallothionein 1M |
| *SLC6A4* | 1.22E-06 | 1.53 | Solute Carrier Family 6 |
| *KIF18A* | 1.83E-04 | 1.53 | Kinesin Family Member 18A |
| *LTBP1* | 4.19E-03 | 1.54 | Latent Transforming Growth Factor Beta Binding Protein 1 |
| *OBSCN* | 2.24E-05 | 1.54 | Obscurin, Cytoskeletal Calmodulin And Titin-Interacting Rhogef |
| *XLOC_002497* | 2.62E-04 | 1.54 | Broad Institute Lincrna |
| *LINC00659* | 1.89E-03 | 1.54 | Long Intergenic Non-Protein Coding Rna 659 |
| *HMMR* | 1.74E-05 | 1.54 | Hyaluronan-Mediated Motility Receptor |
| *COL18A1* | 1.53E-04 | 1.55 | Collagen, Type Xviii, Alpha 1 |
| *XLOC_006009* | 3.07E-03 | 1.55 | Broad Institute Lincrna |
| *IGFL2* | 1.83E-04 | 1.55 | Igf-Like Family Member 2 |
| *LINC00237* | 2.56E-03 | 1.56 | Long Intergenic Non-Protein Coding Rna 237 |
| *TECPR1* | 5.52E-05 | 1.56 | Tectonin Beta-Propeller Repeat Containing 1 |
| *SCN9A* | 3.63E-04 | 1.56 | Sodium Channel, Voltage-Gated, Type Ix, Alpha Subunit |
| *SRGAP2* | 7.44E-05 | 1.56 | Slit-Robo Rho Gtpase Activating Protein 2 |
| *MYLK* | 4.99E-04 | 1.56 | Myosin Light Chain Kinase |
| *COL12A1* | 1.59E-03 | 1.56 | Collagen, Type Xii, Alpha 1 |
| *ABCC9* | 4.49E-03 | 1.57 | Atp-Binding Cassette, Sub-Family C |
| *BMP7* | 1.95E-03 | 1.57 | Bone Morphogenetic Protein 7 |
| *GABRB1* | 2.77E-05 | 1.57 | Gamma-Aminobutyric Acid |
| *NEURL1B* | 1.25E-05 | 1.57 | Neuralized E3 Ubiquitin Protein Ligase 1B |
| *ITGA1* | 1.20E-03 | 1.57 | Integrin, Alpha 1 |
| *ATF7IP2* | 2.94E-03 | 1.57 | Activating Transcription Factor 7 Interacting Protein 2 |
| *C12orf75* | 2.14E-03 | 1.57 | Chromosome 12 Open Reading Frame 75 |
| *PIWIL2* | 2.05E-03 | 1.58 | Predicted: Piwi-Like Rna-Mediated Gene Silencing 2 |
| *EPHX2* | 2.82E-03 | 1.58 | Epoxide Hydrolase 2, Cytoplasmic |
| *GALNT10* | 1.14E-03 | 1.58 | Polypeptide N-Acetylgalactosaminyltransferase 10 |
| *PRDM13* | 3.45E-03 | 1.58 | Pr Domain Containing 13 |
| *RABL6* | 4.57E-04 | 1.58 | Rab, Member Ras Oncogene Family-Like 6 |
| *IGF1* | 2.41E-04 | 1.58 | Insulin-Like Growth Factor 1 |
| *DUT* | 8.28E-05 | 1.59 | Deoxyuridine Triphosphatase |
| *C5orf24* | 1.82E-03 | 1.60 | Chromosome 5 Open Reading Frame 24 |
| *FAM229A* | 3.44E-03 | 1.60 | Family With Sequence Similarity 229, Member A |
| *LINC00668* | 1.41E-03 | 1.60 | Long Intergenic Non-Protein Coding Rna 668 |
| *TUG1* | 4.93E-03 | 1.60 | Taurine Up-Regulated 1 |
| *KISS1R* | 1.45E-04 | 1.61 | Kiss1 Receptor |
| *C5orf66-AS1* | 1.34E-03 | 1.61 | C5Orf66 Antisense Rna 1 |
| *ENOSF1* | 2.70E-03 | 1.61 | Enolase Superfamily Member 1 |
| *PLXNA1* | 4.88E-06 | 1.61 | Plexin A1 |
| *NR2F1* | 4.49E-04 | 1.61 | Nuclear Receptor Subfamily 2, Group F, Member 1 |
| *ARHGAP22* | 2.81E-05 | 1.61 | Rho Gtpase Activating Protein 22 |
| *ACTN3* | 2.77E-03 | 1.61 | Actinin, Alpha 3 |
| *PLK1* | 1.55E-03 | 1.61 | Polo-Like Kinase 1 |
| *MAP6* | 7.10E-05 | 1.62 | Microtubule-Associated Protein 6 |
| *DUT* | 1.25E-03 | 1.62 | Deoxyuridine Triphosphatase |
| *RNF157-AS1* | 3.82E-04 | 1.62 | Rnf157 Antisense Rna 1 |
| *EFR3B* | 2.70E-07 | 1.62 | Efr3 Homolog B |
| *ANKRD29* | 3.56E-03 | 1.62 | Ankyrin Repeat Domain 29 |
| *MYO5C* | 3.21E-04 | 1.62 | Myosin Vc |
| *LOC101929287* | 3.03E-03 | 1.63 | Predicted: Uncharacterized Loc101929287 |
| *NPR2* | 6.19E-05 | 1.63 | Natriuretic Peptide Receptor 2 |
| *DLG2* | 4.55E-04 | 1.63 | Discs, Large Homolog 2 |
| *SBF1* | 4.20E-03 | 1.63 | Set Binding Factor 1 |
| *APH1A* | 8.07E-04 | 1.63 | Aph1A Gamma Secretase Subunit |
| *Loc102723649* | 9.75E-06 | 1.64 | Predicted: Uncharacterized Loc102723649 |
| *CHMP1A* | 6.49E-04 | 1.64 | Charged Multivesicular Body Protein 1A |
| *SLC25A37* | 3.61E-04 | 1.64 | Solute Carrier Family 25 |
| *KRTAP2-3* | 3.28E-03 | 1.64 | Keratin Associated Protein 2-3 |
| *LYPD1* | 4.92E-06 | 1.64 | Ly6/Plaur Domain Containing 1 |
| *CCR1* | 2.02E-03 | 1.65 | Chemokine |
| *CDKN2C* | 2.36E-03 | 1.65 | Cyclin-Dependent Kinase Inhibitor 2C |
| *COL5A2* | 4.55E-06 | 1.66 | Collagen, Type V, Alpha 2 |
| *LRRC7* | 1.46E-04 | 1.66 | Leucine Rich Repeat Containing 7 |
| *CXCL12* | 1.63E-05 | 1.66 | Chemokine |
| *LINC00434* | 3.26E-03 | 1.67 | Long Intergenic Non-Protein Coding Rna 434 |
| *KCTD12* | 5.97E-06 | 1.67 | Potassium Channel Tetramerization Domain Containing 12 |
| *FLJ45513* | 2.50E-04 | 1.68 | Uncharacterized Loc729220 |
| *KCTD12* | 1.38E-04 | 1.68 | Potassium Channel Tetramerization Domain Containing 12 |
| *C21orf33* | 1.21E-04 | 1.68 | Chromosome 21 Open Reading Frame 33 |
| *LOC283070* | 1.25E-04 | 1.69 | Uncharacterized Loc283070 |
| *HSPG2* | 2.38E-03 | 1.69 | Heparan Sulfate Proteoglycan 2 |
| *USP27X* | 5.85E-06 | 1.69 | Ubiquitin Specific Peptidase 27, X-Linked |
| *LTBP1* | 1.93E-03 | 1.69 | Latent Transforming Growth Factor Beta Binding Protein 1 |
| *LIFR* | 1.82E-03 | 1.69 | Leukemia Inhibitory Factor Receptor Alpha |
| *EMX2OS* | 1.02E-04 | 1.70 | Emx2 Opposite Strand/Antisense Rna |
| *AKT2* | 4.74E-03 | 1.71 | V-Akt Murine Thymoma Viral Oncogene Homolog 2 |
| *TTC12* | 2.28E-03 | 1.72 | Tetratricopeptide Repeat Domain 12 |
| *TUSC8* | 1.11E-03 | 1.72 | Tumor Suppressor Candidate 8 |
| *ZBED2* | 1.60E-03 | 1.72 | Zinc Finger, Bed-Type Containing 2 |
| *LYPD1* | 4.91E-05 | 1.72 | Ly6/Plaur Domain Containing 1 |
| *COMTD1* | 8.83E-04 | 1.73 | Catechol-O-Methyltransferase Domain Containing 1 |
| *MRAP2* | 3.05E-06 | 1.73 | Melanocortin 2 Receptor Accessory Protein 2 |
| *HLF* | 2.37E-04 | 1.73 | Hepatic Leukemia Factor |
| *LIMK1* | 1.63E-03 | 1.74 | Lim Domain Kinase 1 |
| *LINC00313* | 5.14E-07 | 1.74 | Long Intergenic Non-Protein Coding Rna 313 |
| *FSHR* | 7.69E-05 | 1.74 | Follicle Stimulating Hormone Receptor |
| *ALDH1L2* | 2.46E-03 | 1.74 | Aldehyde Dehydrogenase 1 Family, Member L2 |
| *SLC35D3* | 4.67E-06 | 1.75 | Solute Carrier Family 35, Member D3 |
| *PTPRD* | 1.99E-06 | 1.76 | Protein Tyrosine Phosphatase, Receptor Type, D |
| *TPCN1* | 1.48E-04 | 1.76 | Two Pore Segment Channel 1 |
| *FLNA* | 4.86E-04 | 1.77 | Filamin A, Alpha |
| *MX1* | 1.70E-04 | 1.78 | Myxovirus |
| *AGPAT4* | 3.02E-03 | 1.78 | 1-Acylglycerol-3-Phosphate O-Acyltransferase 4 |
| *ERGIC1* | 6.68E-04 | 1.78 | Endoplasmic Reticulum-Golgi Intermediate Compartment |
| *MCM7* | 1.55E-03 | 1.79 | Minichromosome Maintenance Complex Component 7 |
| *FOXJ1* | 2.87E-04 | 1.79 | Forkhead Box J1 |
| *ABAT* | 2.08E-04 | 1.79 | 4-Aminobutyrate Aminotransferase |
| *LOC101927820* | 1.17E-03 | 1.80 | Predicted: Uncharacterized Loc101927820 |
| *MIR4697HG* | 1.15E-03 | 1.80 | Mir4697 Host Gene |
| *ARHGEF39* | 2.63E-04 | 1.81 | Rho Guanine Nucleotide Exchange Factor |
| *FRY* | 3.99E-06 | 1.81 | Furry Homolog |
| *WNT6* | 2.40E-06 | 1.81 | Wingless-Type Mmtv Integration Site Family, Member 6 |
| *ISPD* | 1.09E-04 | 1.81 | Isoprenoid Synthase Domain Containing |
| *MYEF2* | 3.19E-03 | 1.82 | Myelin Expression Factor 2 |
| *KCNMA1* | 1.71E-03 | 1.82 | Potassium Large Conductance Calcium-Activated Channel, Subfamily M, Alpha Member 1 |
| *LOC340340* | 1.56E-03 | 1.82 |  |
| *PPP3R1* | 2.67E-03 | 1.82 | Protein Phosphatase 3, Regulatory Subunit B, Alpha |
| *BANK1* | 3.68E-03 | 1.83 | B-Cell Scaffold Protein With Ankyrin Repeats 1 |
| *APH1A* | 1.49E-04 | 1.84 | Aph1A Gamma Secretase Subunit |
| *VIM* | 5.54E-04 | 1.84 | Vimentin |
| *SEPT9* | 2.64E-03 | 1.86 | Septin 9 |
| *H1F0* | 1.20E-03 | 1.89 | H1 Histone Family, Member 0 |
| *APELA* | 6.62E-05 | 1.90 | Apelin Receptor Early Endogenous Ligand |
| *APELA* | 4.42E-05 | 1.91 | Apelin Receptor Early Endogenous Ligand |
| *APELA* | 6.35E-05 | 1.91 |  |
| *UBE2E1* | 4.19E-03 | 1.91 | Ubiquitin-Conjugating Enzyme E2E 1 |
| *CAMK1D* | 5.06E-05 | 1.92 | Calcium/Calmodulin-Dependent Protein Kinase Id |
| *HCRTR2* | 1.63E-03 | 1.94 | Hypocretin |
| *ANXA8L1* | 2.44E-06 | 1.95 | Annexin A8-Like 1 |
| *PLP1* | 7.74E-04 | 1.95 | Proteolipid Protein 1 |
| *DHRS2* | 5.37E-05 | 1.96 | Dehydrogenase/Reductase |
| *CDH6* | 2.57E-04 | 1.97 | Cadherin 6, Type 2, K-Cadherin |
| *TGFB2* | 3.46E-04 | 1.97 | Transforming Growth Factor, Beta 2 |
| *GATM* | 3.08E-03 | 1.98 | Glycine Amidinotransferase |
| *NEBL* | 4.27E-05 | 1.99 | Nebulette |
| *No name* | 4.76E-04 | 2.01 | Agencourt_14301491 Nih_Mgc_173 Cdna 5', Mrna Sequence |
| *MMP14* | 1.94E-03 | 2.01 | Matrix Metallopeptidase 14 |
| *SLC10A1* | 5.44E-04 | 2.05 | Solute Carrier Family 10 |
| *COL4A6* | 4.07E-03 | 2.07 | Collagen, Type Iv, Alpha 6 |
| *RFX6* | 2.06E-03 | 2.07 | Regulatory Factor X, 6 |
| *GGACT* | 1.79E-03 | 2.09 | Gamma-Glutamylamine Cyclotransferase |
| *MYBPH* | 5.72E-06 | 2.10 | Myosin Binding Protein H |
| *DNAH2* | 3.33E-05 | 2.10 | Dynein, Axonemal, Heavy Chain 2 |
| *IL17RE* | 1.41E-03 | 2.18 | Interleukin 17 Receptor E |
| *GADL1* | 1.70E-05 | 2.19 | Glutamate Decarboxylase-Like 1 |
| *HCN4* | 2.91E-03 | 2.22 | Hyperpolarization Activated Cyclic Nucleotide-Gated Potassium Channel 4 |
| *NUTM2F* | 2.35E-03 | 2.23 | Nut Family Member 2F |
| *CNN1* | 7.33E-06 | 2.27 | Calponin 1, Basic, Smooth Muscle |
| *KIT* | 2.29E-05 | 2.27 | V-Kit Hardy-Zuckerman 4 Feline Sarcoma Viral Oncogene Homolog |
| *INCENP* | 4.11E-03 | 2.34 | Inner Centromere Protein Antigens 135/155Kda |
| *SRC* | 1.04E-03 | 2.36 | V-Src Avian Sarcoma |
| *SPEF2* | 3.78E-03 | 2.36 | Sperm Flagellar 2 |
| *LINC00313* | 1.36E-04 | 2.42 | Long Intergenic Non-Protein Coding Rna 313 |
| *PHKA2-AS1* | 1.13E-03 | 2.45 | Phka2 Antisense Rna 1 |
| *IFIT1* | 3.74E-05 | 2.70 | Interferon-Induced Protein With Tetratricopeptide Repeats 1 |
| *PBX2* | 5.78E-04 | 2.74 | Pre-B-Cell Leukemia Homeobox 2 |
| *LOC729159* | 5.26E-04 | 2.96 | Upf0607 Protein Ensp00000381418-Like |
| *ZC3H7B* | 3.04E-04 | 3.07 | Zinc Finger Ccch-Type Containing 7B |
| *DZIP1* | 1.84E-03 | 3.82 | Daz Interacting Zinc Finger Protein 1 |
| Statistical significance of treatment: p < 0.05 | | | |
| *CCND1* | 1.79E-01 | -1.14 | Cyclin D1 |
| *FABP4* | 9.39E-02 | -1.20 | Fatty acid binding protein 4 |
| *UCP1* | 2.69E-01 | -1.51 | Uncoupling protein 1 |

p-value >0.05
