# Supplementary material for: Transcriptome Profiling of Caco-2 Cancer Cell Line following Treatment with Extracts from Iodine-Biofortified Lettuce (Lactuca sativa L.)
Source: PLoS One. 2016 Jan 22;11(1):e0147336. doi: 10.1371/journal.pone.0147336 (PMC4723252; doi:10.1371/journal.pone.0147336)
Supplement: S3 Table — Statistical significance of treatment: p < 0.05. (DOCX) [file pone.0147336.s003.docx]

**S3 Table. GO biological processes based on BFL vs. NFL specific genes differently regulated in Caco-2 cell line.**

| **Biological Process** | **The number of involved genes** | **The number of regulated genes** | ***p*-value** |
| --- | --- | --- | --- |
| Primary Metabolic Process | 7177 | 438 | 1.12E-07 |
| Unclassified | 9422 | 389 | 6.83E-07 |
| Cellular Process | 5952 | 339 | 1.82E-03 |
| Nucleobase-Containing Compound Metabolic Process | 3532 | 218 | 3.48E-04 |
| Biological Regulation | 3252 | 193 | 4.65E-03 |
| Cell Communication | 3221 | 174 | 1.22E-01 |
| Protein Metabolic Process | 2807 | 166 | 9.88E-03 |
| Developmental Process | 2846 | 153 | 1.55E-01 |
| Localization | 2636 | 147 | 7.34E-02 |
| Transport | 2564 | 145 | 5.36E-02 |
| Rna Metabolic Process | 2473 | 133 | 1.74E-01 |
| Regulation Of Biological Process | 2175 | 129 | 2.04E-02 |
| Cell Cycle | 1399 | 113 | 3.75E-07 |
| Transcription, Dna-Dependent | 1987 | 111 | 1.06E-01 |
| Transcription From Rna Polymerase Ii Promoter | 1976 | 110 | 1.14E-01 |
| Regulation Of Nucleobase-Containing Compound Metabolic Process | 1648 | 94 | 9.08E-02 |
| Regulation Of Transcription From Rna Polymerase Ii Promoter | 1546 | 88 | 1.02E-01 |
| Multicellular Organismal Process | 1798 | 88 | 4.74E-01 |
| Single-Multicellular Organism Process | 1798 | 88 | 4.74E-01 |
| Cellular Protein Modification Process | 1204 | 83 | 1.93E-03 |
| Cellular Component Organization Or Biogenesis | 1137 | 81 | 8.96E-04 |
| Cellular Component Organization | 1065 | 80 | 2.09E-04 |
| Response To Stimulus | 1671 | 76 | 2.34E-01 |
| System Process | 1481 | 73 | 5.07E-01 |
| System Development | 1645 | 72 | 1.46E-01 |
| Immune System Process | 1733 | 71 | 4.86E-02 |
| Intracellular Protein Transport | 1322 | 68 | 3.98E-01 |
| Protein Transport | 1339 | 68 | 4.40E-01 |
| Regulation Of Molecular Function | 1140 | 62 | 2.47E-01 |
| Regulation Of Catalytic Activity | 1119 | 61 | 2.43E-01 |
| Neurological System Process | 1212 | 58 | 4.21E-01 |
| Lipid Metabolic Process | 902 | 57 | 4.03E-02 |
| Anatomical Structure Morphogenesis | 691 | 53 | 1.53E-03 |
| Vesicle-Mediated Transport | 928 | 53 | 1.65E-01 |
| Mitosis | 528 | 51 | 8.60E-06 |
| Cell Adhesion | 890 | 50 | 2.04E-01 |
| Biological Adhesion | 890 | 50 | 2.04E-01 |
| Cellular Component Morphogenesis | 646 | 49 | 2.79E-03 |
| Ion Transport | 688 | 47 | 1.94E-02 |
| Cell Death | 697 | 47 | 2.36E-02 |
| Apoptotic Process | 697 | 47 | 2.36E-02 |
| Death | 699 | 47 | 2.47E-02 |
| Proteolysis | 899 | 47 | 3.78E-01 |
| Cation Transport | 595 | 42 | 1.64E-02 |
| Nervous System Development | 1008 | 41 | 1.06E-01 |
| Ectoderm Development | 909 | 40 | 2.45E-01 |
| Mesoderm Development | 959 | 38 | 8.55E-02 |
| Carbohydrate Metabolic Process | 650 | 38 | 1.73E-01 |
| Cell-Cell Signaling | 835 | 37 | 2.71E-01 |
| Cellular Component Movement | 493 | 35 | 2.47E-02 |
| Reproduction | 616 | 34 | 2.88E-01 |
| Cellular Amino Acid Metabolic Process | 368 | 33 | 1.09E-03 |
| Phosphate-Containing Compound Metabolic Process | 520 | 33 | 9.45E-02 |
| Protein Phosphorylation | 550 | 33 | 1.56E-01 |
| Gamete Generation | 534 | 31 | 2.12E-01 |
| Cell-Cell Adhesion | 506 | 29 | 2.41E-01 |
| Endocytosis | 397 | 26 | 9.73E-02 |
| Response To Stress | 439 | 25 | 2.71E-01 |
| Dna Metabolic Process | 434 | 24 | 3.24E-01 |
| Cytokinesis | 187 | 22 | 2.46E-04 |
| Nitrogen Compound Metabolic Process | 308 | 22 | 6.07E-02 |
| Organelle Organization | 318 | 21 | 1.18E-01 |
| Synaptic Transmission | 415 | 21 | 4.94E-01 |
| Generation Of Precursor Metabolites And Energy | 280 | 20 | 7.10E-02 |
| Chromosome Segregation | 181 | 18 | 5.03E-03 |
| Respiratory Electron Transport Chain | 232 | 18 | 4.52E-02 |
| Mrna Processing | 438 | 18 | 2.47E-01 |
| Muscle Organ Development | 390 | 18 | 4.37E-01 |
| Induction Of Apoptosis | 228 | 17 | 6.73E-02 |
| Sensory Perception | 442 | 17 | 1.70E-01 |
| Translation | 410 | 16 | 1.97E-01 |
| Muscle Contraction | 280 | 16 | 3.20E-01 |
| Negative Regulation Of Apoptotic Process | 186 | 15 | 4.87E-02 |
| Steroid Metabolic Process | 219 | 15 | 1.35E-01 |
| Mrna Splicing, Via Spliceosome | 368 | 15 | 2.64E-01 |
| Chromatin Organization | 257 | 15 | 2.99E-01 |
| Response To External Stimulus | 276 | 15 | 3.97E-01 |
| Lipid Transport | 301 | 15 | 5.29E-01 |
| Immune Response | 632 | 14 | 3.67E-04 |
| Cellular Defense Response | 387 | 13 | 8.90E-02 |
| Dna Replication | 221 | 13 | 3.07E-01 |
| Spermatogenesis | 223 | 13 | 3.18E-01 |
| Visual Perception | 303 | 13 | 3.58E-01 |
| Embryo Development | 187 | 12 | 2.24E-01 |
| Blood Coagulation | 220 | 12 | 4.11E-01 |
| Polysaccharide Metabolic Process | 228 | 12 | 4.58E-01 |
| Response To Toxic Substance | 61 | 11 | 3.07E-04 |
| Skeletal System Development | 334 | 11 | 9.92E-02 |
| Macrophage Activation | 289 | 11 | 2.31E-01 |
| Fatty Acid Metabolic Process | 188 | 11 | 3.33E-01 |
| Protein Folding | 194 | 11 | 3.70E-01 |
| Cellular Amino Acid Biosynthetic Process | 125 | 10 | 9.80E-02 |
| Female Gamete Generation | 144 | 10 | 1.84E-01 |
| Angiogenesis | 246 | 10 | 3.25E-01 |
| Heart Development | 240 | 10 | 3.56E-01 |
| B Cell Mediated Immunity | 234 | 10 | 3.88E-01 |
| Extracellular Transport | 125 | 9 | 1.74E-01 |
| Homeostatic Process | 136 | 9 | 2.38E-01 |
| Catabolic Process | 157 | 9 | 3.78E-01 |
| Receptor-Mediated Endocytosis | 193 | 9 | 5.11E-01 |
| Protein Glycosylation | 177 | 9 | 5.16E-01 |
| Rna Splicing | 272 | 8 | 7.78E-02 |
| Rna Splicing, Via Transesterification Reactions | 272 | 8 | 7.78E-02 |
| Phosphate Ion Transport | 98 | 8 | 1.19E-01 |
| Pattern Specification Process | 244 | 8 | 1.47E-01 |
| Rrna Metabolic Process | 127 | 8 | 2.98E-01 |
| Regulation Of Translation | 135 | 8 | 3.56E-01 |
| Dna Repair | 191 | 8 | 3.94E-01 |
| Phospholipid Metabolic Process | 190 | 8 | 4.00E-01 |
| Neurotransmitter Secretion | 190 | 8 | 4.00E-01 |
| Hemopoiesis | 155 | 8 | 5.04E-01 |
| Pyrimidine Nucleobase Metabolic Process | 61 | 7 | 3.47E-02 |
| Cellular Amino Acid Catabolic Process | 69 | 7 | 5.93E-02 |
| Cell-Matrix Adhesion | 115 | 7 | 3.47E-01 |
| Monosaccharide Metabolic Process | 135 | 7 | 5.05E-01 |
| Anion Transport | 141 | 7 | 5.50E-01 |
| Biosynthetic Process | 143 | 7 | 5.84E-01 |
| Exocytosis | 275 | 6 | 1.71E-02 |
| Sensory Perception Of Sound | 78 | 6 | 1.95E-01 |
| Cell Differentiation | 93 | 6 | 3.17E-01 |
| Carbohydrate Transport | 94 | 6 | 3.25E-01 |
| Cyclic Nucleotide Metabolic Process | 99 | 6 | 3.69E-01 |
| Meiosis | 100 | 6 | 3.77E-01 |
| Blood Circulation | 149 | 6 | 3.92E-01 |
| Purine Nucleobase Metabolic Process | 103 | 6 | 4.04E-01 |
| Phagocytosis | 30 | 5 | 1.80E-02 |
| Cellular Calcium Ion Homeostasis | 49 | 5 | 9.96E-02 |
| Segment Specification | 172 | 5 | 1.46E-01 |
| Protein Targeting | 134 | 5 | 3.47E-01 |
| Sulfur Compound Metabolic Process | 84 | 5 | 4.04E-01 |
| Nucleobase-Containing Compound Transport | 110 | 5 | 5.36E-01 |
| Porphyrin-Containing Compound Metabolic Process | 31 | 4 | 7.04E-02 |
| Regulation Of Liquid Surface Tension | 44 | 4 | 1.77E-01 |
| Cell Proliferation | 45 | 4 | 1.87E-01 |
| Cholesterol Metabolic Process | 108 | 4 | 3.79E-01 |
| Trna Metabolic Process | 65 | 4 | 4.03E-01 |
| Mrna 3'-End Processing | 94 | 4 | 5.01E-01 |
| Mrna Polyadenylation | 90 | 4 | 5.38E-01 |
| Neuron-Neuron Synaptic Transmission | 90 | 4 | 5.38E-01 |
| Amino Acid Transport | 83 | 4 | 6.06E-01 |
| Vitamin Transport | 81 | 4 | 6.25E-01 |
| Endoderm Development | 23 | 3 | 1.08E-01 |
| Cellular Component Biogenesis | 114 | 3 | 1.84E-01 |
| Coenzyme Metabolic Process | 101 | 3 | 2.63E-01 |
| Vitamin Biosynthetic Process | 39 | 3 | 3.06E-01 |
| Glycogen Metabolic Process | 90 | 3 | 3.48E-01 |
| Vitamin Metabolic Process | 52 | 3 | 4.77E-01 |
| Dna Catabolic Process | 8 | 2 | 6.07E-02 |
| Ferredoxin Metabolic Process | 11 | 2 | 1.04E-01 |
| Protein Methylation | 12 | 2 | 1.20E-01 |
| Dna Recombination | 87 | 2 | 1.95E-01 |
| Neuronal Action Potential Propagation | 21 | 2 | 2.80E-01 |
| Mitochondrion Organization | 21 | 2 | 2.80E-01 |
| Mitochondrial Transport | 21 | 2 | 2.80E-01 |
| Protein Lipidation | 23 | 2 | 3.16E-01 |
| Regulation Of Phosphate Metabolic Process | 67 | 2 | 3.54E-01 |
| Rna Catabolic Process | 61 | 2 | 4.17E-01 |
| Oxidative Phosphorylation | 57 | 2 | 4.62E-01 |
| Digestive Tract Mesoderm Development | 54 | 2 | 4.98E-01 |
| Fatty Acid Beta-Oxidation | 35 | 2 | 5.18E-01 |
| Protein Acetylation | 36 | 2 | 5.33E-01 |
| Dorsal/Ventral Axis Specification | 39 | 2 | 5.76E-01 |
| Anterior/Posterior Axis Specification | 44 | 2 | 6.27E-01 |
| Nuclear Transport | 121 | 1 | 1.71E-02 |
| Natural Killer Cell Activation | 103 | 1 | 3.65E-02 |
| Synaptic Vesicle Exocytosis | 100 | 1 | 4.14E-02 |
| Response To Biotic Stimulus | 1 | 1 | 4.84E-02 |
| Rna Localization | 82 | 1 | 8.62E-02 |
| Fertilization | 82 | 1 | 8.62E-02 |
| Cytokine Production | 2 | 1 | 9.45E-02 |
| Protein Complex Biogenesis | 79 | 1 | 9.72E-02 |
| Protein Complex Assembly | 79 | 1 | 9.72E-02 |
| Complement Activation | 77 | 1 | 1.05E-01 |
| Regulation Of Sequence-Specific Dna Binding Transcription Factor Activity | 3 | 1 | 1.38E-01 |
| Regulation Of Vasoconstriction | 51 | 1 | 2.81E-01 |
| Nitric Oxide Biosynthetic Process | 8 | 1 | 3.28E-01 |
| Pentose-Phosphate Shunt | 8 | 1 | 3.28E-01 |
| Regulation Of Cellular Amino Acid Metabolic Process | 10 | 1 | 3.91E-01 |
| Fatty Acid Biosynthetic Process | 41 | 1 | 3.96E-01 |
| Regulation Of Cell Cycle | 13 | 1 | 4.75E-01 |
| Sex Determination | 13 | 1 | 4.75E-01 |
| Peroxisomal Transport | 33 | 1 | 5.13E-01 |
| Neuromuscular Synaptic Transmission | 15 | 1 | 5.25E-01 |
| Gluconeogenesis | 32 | 1 | 5.29E-01 |
| Response To Endogenous Stimulus | 16 | 1 | 5.48E-01 |
| Cytoskeleton Organization | 16 | 1 | 5.48E-01 |
| Acyl-Coa Metabolic Process | 30 | 1 | 5.61E-01 |
| Mrna Transcription | 29 | 1 | 5.78E-01 |
| Locomotion | 18 | 1 | 5.91E-01 |
| Glycolysis | 25 | 1 | 6.48E-01 |
| Tricarboxylic Acid Cycle | 23 | 1 | 6.84E-01 |
| Response To Abiotic Stimulus | 21 | 1 | 7.20E-01 |
| Regulation Of Carbohydrate Metabolic Process | 21 | 1 | 7.20E-01 |

Statistical significance of treatment: p < 0.05
