# Supplementary material for: Transcriptome Profiling of Caco-2 Cancer Cell Line following Treatment with Extracts from Iodine-Biofortified Lettuce (Lactuca sativa L.)
Source: PLoS One. 2016 Jan 22;11(1):e0147336. doi: 10.1371/journal.pone.0147336 (PMC4723252; doi:10.1371/journal.pone.0147336)
Supplement: S4 Table — Statistical significance of treatment: p < 0.05. (DOCX) [file pone.0147336.s004.docx]

**S4 Table. GO molecular functions based on BFL vs. NFL specific genes differently regulated in Caco-2 cell line.**

| **Molecular Function** | **The number of involved genes** | **The numbers of regulated genes** | ***p*-value** |
| --- | --- | --- | --- |
| Catalytic Activity | 5529 | 369 | 9.26E-11 |
| Binding | 5933 | 323 | 2.84E-02 |
| Nucleic Acid Binding | 3315 | 189 | 2.26E-02 |
| Hydrolase Activity | 2332 | 155 | 1.25E-04 |
| Protein Binding | 2855 | 151 | 2.12E-01 |
| Dna Binding | 2032 | 121 | 2.20E-02 |
| Sequence-Specific Dna Binding Transcription Factor Activity | 1754 | 106 | 2.18E-02 |
| Nucleic Acid Binding Transcription Factor Activity | 1762 | 106 | 2.43E-02 |
| Transferase Activity | 1614 | 99 | 1.86E-02 |
| Structural Molecule Activity | 1261 | 88 | 1.00E-03 |
| Transporter Activity | 1145 | 71 | 3.44E-02 |
| Receptor Activity | 1576 | 70 | 1.84E-01 |
| Structural Constituent Of Cytoskeleton | 851 | 64 | 8.53E-04 |
| Transmembrane Transporter Activity | 1076 | 64 | 8.09E-02 |
| Enzyme Regulator Activity | 1091 | 60 | 2.25E-01 |
| Oxidoreductase Activity | 634 | 58 | 1.03E-05 |
| Receptor Binding | 1017 | 46 | 2.89E-01 |
| Hydrolase Activity, Acting On Ester Bonds | 678 | 44 | 4.65E-02 |
| Peptidase Activity | 747 | 42 | 2.26E-01 |
| Kinase Activity | 814 | 40 | 5.17E-01 |
| Ligase Activity | 500 | 33 | 6.40E-02 |
| Small Gtpase Regulator Activity | 400 | 29 | 3.04E-02 |
| Cation Transmembrane Transporter Activity | 368 | 26 | 4.96E-02 |
| Rna Binding | 639 | 25 | 1.30E-01 |
| Protein Kinase Activity | 414 | 25 | 1.87E-01 |
| Pyrophosphatase Activity | 271 | 22 | 1.90E-02 |
| Cytoskeletal Protein Binding | 271 | 21 | 3.30E-02 |
| Serine-Type Peptidase Activity | 314 | 21 | 1.08E-01 |
| Phosphatase Activity | 300 | 20 | 1.17E-01 |
| Calcium Ion Binding | 445 | 20 | 3.79E-01 |
| Ubiquitin-Protein Ligase Activity | 279 | 18 | 1.61E-01 |
| Motor Activity | 145 | 17 | 1.23E-03 |
| Phosphoprotein Phosphatase Activity | 179 | 17 | 9.59E-03 |
| Chromatin Binding | 194 | 17 | 1.93E-02 |
| Ion Channel Activity | 354 | 17 | 5.09E-01 |
| Transferase Activity, Transferring Acyl Groups | 179 | 16 | 1.94E-02 |
| Actin Binding | 194 | 16 | 3.63E-02 |
| Transferase Activity, Transferring Glycosyl Groups | 229 | 16 | 1.12E-01 |
| Guanyl-Nucleotide Exchange Factor Activity | 166 | 15 | 2.12E-02 |
| Nuclease Activity | 196 | 15 | 6.91E-02 |
| Calmodulin Binding | 262 | 13 | 5.73E-01 |
| Methyltransferase Activity | 122 | 12 | 2.10E-02 |
| Transcription Factor Binding Transcription Factor Activity | 192 | 12 | 2.50E-01 |
| Protein Binding Transcription Factor Activity | 192 | 12 | 2.50E-01 |
| Transcription Cofactor Activity | 192 | 12 | 2.50E-01 |
| Lyase Activity | 209 | 12 | 3.46E-01 |
| Gtpase Activity | 254 | 12 | 5.07E-01 |
| Structural Constituent Of Ribosome | 204 | 11 | 4.32E-01 |
| Acetyltransferase Activity | 102 | 10 | 3.38E-02 |
| Isomerase Activity | 169 | 10 | 3.32E-01 |
| Enzyme Inhibitor Activity | 340 | 9 | 2.70E-02 |
| Mrna Binding | 313 | 9 | 5.31E-02 |
| Kinase Regulator Activity | 261 | 9 | 1.67E-01 |
| Growth Factor Activity | 171 | 9 | 4.75E-01 |
| Microtubule Motor Activity | 86 | 8 | 6.83E-02 |
| Calcium-Dependent Phospholipid Binding | 124 | 8 | 2.77E-01 |
| Translation Regulator Activity | 132 | 8 | 3.34E-01 |
| Translation Factor Activity, Nucleic Acid Binding | 137 | 8 | 3.71E-01 |
| Non-Membrane Spanning Protein Tyrosine Kinase Activity | 96 | 7 | 2.04E-01 |
| Metallopeptidase Activity | 198 | 7 | 2.35E-01 |
| Peptidase Inhibitor Activity | 193 | 7 | 2.60E-01 |
| Amino Acid Transmembrane Transporter Activity | 109 | 7 | 2.99E-01 |
| Voltage-Gated Ion Channel Activity | 133 | 7 | 4.89E-01 |
| Cysteine-Type Peptidase Activity | 149 | 7 | 5.40E-01 |
| Cation Channel Activity | 147 | 7 | 5.55E-01 |
| G-Protein Coupled Receptor Activity | 284 | 6 | 1.30E-02 |
| Ligand-Activated Sequence-Specific Dna Binding Rna Polymerase Ii Transcription Factor Activity | 48 | 6 | 3.44E-02 |
| Racemase And Epimerase Activity | 58 | 6 | 7.19E-02 |
| Sequence-Specific Dna Binding Rna Polymerase Ii Transcription Factor Activity | 67 | 6 | 1.20E-01 |
| Extracellular Matrix Structural Constituent | 91 | 6 | 3.00E-01 |
| Enzyme Activator Activity | 146 | 6 | 4.13E-01 |
| Helicase Activity | 146 | 6 | 4.13E-01 |
| Microtubule Binding | 64 | 5 | 2.15E-01 |
| Rna Helicase Activity | 89 | 5 | 4.52E-01 |
| Translation Initiation Factor Activity | 102 | 5 | 6.05E-01 |
| Atpase Activity, Coupled To Transmembrane Movement Of Substances | 34 | 4 | 9.11E-02 |
| Dna-Directed Rna Polymerase Activity | 43 | 4 | 1.68E-01 |
| Endoribonuclease Activity | 50 | 4 | 2.38E-01 |
| Hydro-Lyase Activity | 56 | 4 | 3.03E-01 |
| Nucleotide Kinase Activity | 58 | 4 | 3.25E-01 |
| Phospholipase Activity | 70 | 4 | 4.58E-01 |
| Ligand-Gated Ion Channel Activity | 97 | 4 | 4.73E-01 |
| Kinase Activator Activity | 94 | 4 | 5.01E-01 |
| Lipase Activity | 87 | 4 | 5.67E-01 |
| Poly(A) Rna Binding | 86 | 4 | 5.77E-01 |
| Phosphorylase Activity | 12 | 3 | 2.26E-02 |
| Structural Constituent Of Myelin Sheath | 21 | 3 | 8.81E-02 |
| Guanylate Cyclase Activity | 26 | 3 | 1.41E-01 |
| Double-Stranded Dna Binding | 29 | 3 | 1.76E-01 |
| Hormone Activity | 108 | 3 | 2.17E-01 |
| Anion Channel Activity | 35 | 3 | 2.52E-01 |
| Voltage-Gated Sodium Channel Activity | 37 | 3 | 2.79E-01 |
| Voltage-Gated Calcium Channel Activity | 38 | 3 | 2.92E-01 |
| Phosphoric Diester Hydrolase Activity | 40 | 3 | 3.19E-01 |
| Lipid Transporter Activity | 92 | 3 | 3.31E-01 |
| Dna Replication Origin Binding | 44 | 3 | 3.73E-01 |
| Hydrogen Ion Transmembrane Transporter Activity | 45 | 3 | 3.86E-01 |
| Deaminase Activity | 46 | 3 | 3.99E-01 |
| Transmembrane Receptor Protein Kinase Activity | 48 | 3 | 4.26E-01 |
| Adenylate Cyclase Activity | 67 | 3 | 5.75E-01 |
| Cytokine Receptor Activity | 66 | 3 | 5.86E-01 |
| Cytokine Activity | 188 | 2 | 4.66E-03 |
| Translation Release Factor Activity | 7 | 2 | 4.80E-02 |
| Transmembrane Receptor Protein Tyrosine Kinase Activity | 17 | 2 | 2.07E-01 |
| Kinase Inhibitor Activity | 84 | 2 | 2.14E-01 |
| Dna-Methyltransferase Activity | 18 | 2 | 2.25E-01 |
| Rna Methyltransferase Activity | 19 | 2 | 2.43E-01 |
| Nucleotidyltransferase Activity | 78 | 2 | 2.57E-01 |
| Single-Stranded Dna Binding | 74 | 2 | 2.90E-01 |
| Dna Helicase Activity | 74 | 2 | 2.90E-01 |
| Aspartic-Type Endopeptidase Activity | 22 | 2 | 2.98E-01 |
| Tumor Necrosis Factor-Activated Receptor Activity | 22 | 2 | 2.98E-01 |
| Transaminase Activity | 26 | 2 | 3.70E-01 |
| Lipid Binding | 26 | 2 | 3.70E-01 |
| Carboxy-Lyase Activity | 26 | 2 | 3.70E-01 |
| Peroxidase Activity | 27 | 2 | 3.87E-01 |
| Carbohydrate Transmembrane Transporter Activity | 60 | 2 | 4.28E-01 |
| Antioxidant Activity | 30 | 2 | 4.39E-01 |
| Exoribonuclease Activity | 32 | 2 | 4.71E-01 |
| Nucleotide Phosphatase Activity | 34 | 2 | 5.03E-01 |
| Nucleotide Binding | 37 | 2 | 5.48E-01 |
| Acetylcholine Receptor Activity | 46 | 2 | 6.01E-01 |
| Gaba Receptor Activity | 46 | 2 | 6.01E-01 |
| Translation Elongation Factor Activity | 44 | 2 | 6.27E-01 |
| Dna Polymerase Processivity Factor Activity | 1 | 1 | 4.84E-02 |
| Dna Primase Activity | 1 | 1 | 4.84E-02 |
| Serine-Type Endopeptidase Inhibitor Activity | 83 | 1 | 8.29E-02 |
| Voltage-Gated Potassium Channel Activity | 60 | 1 | 2.02E-01 |
| Chemokine Activity | 50 | 1 | 2.91E-01 |
| Galactosidase Activity | 8 | 1 | 3.28E-01 |
| Hydrolase Activity, Hydrolyzing N-Glycosyl Compounds | 41 | 1 | 3.96E-01 |
| Carbohydrate Phosphatase Activity | 13 | 1 | 4.75E-01 |
| Carbohydrate Kinase Activity | 34 | 1 | 4.97E-01 |
| Protein Disulfide Isomerase Activity | 16 | 1 | 5.48E-01 |
| Centromeric Dna Binding | 17 | 1 | 5.70E-01 |
| Dna-Directed Dna Polymerase Activity | 29 | 1 | 5.78E-01 |
| Endodeoxyribonuclease Activity | 19 | 1 | 6.11E-01 |
| Deacetylase Activity | 27 | 1 | 6.13E-01 |
| Proton-Transporting Atp Synthase Activity, Rotational Mechanism | 20 | 1 | 6.30E-01 |
| Hydrolase Activity, Hydrolyzing O-Glycosyl Compounds | 26 | 1 | 6.30E-01 |
| Neuropeptide Hormone Activity | 25 | 1 | 6.48E-01 |
| Glutamate Receptor Activity | 23 | 1 | 6.84E-01 |
| Transmembrane Receptor Protein Serine/Threonine Kinase Activity | 22 | 1 | 7.02E-01 |
| Cysteine-Type Endopeptidase Inhibitor Activity | 21 | 1 | 7.20E-01 |
| Gap Junction Channel Activity | 21 | 1 | 7.20E-01 |

Statistical significance of treatment: p < 0.05
