# Supplementary material for: Transcriptome Profiling of Caco-2 Cancer Cell Line following Treatment with Extracts from Iodine-Biofortified Lettuce (Lactuca sativa L.)
Source: PLoS One. 2016 Jan 22;11(1):e0147336. doi: 10.1371/journal.pone.0147336 (PMC4723252; doi:10.1371/journal.pone.0147336)
Supplement: S5 Table — Statistical significance of treatment: p < 0.05. (DOCX) [file pone.0147336.s005.docx]

**S5 Table. Protein classes based on BFL vs. NFL specific genes differently regulated in Caco-2 cell line.**

| **Protein Class** | **The number of genes involved in pathway** | **The numbers of regulated genes** | ***p*-value** |
| --- | --- | --- | --- |
|  |  |  |  |
| Hydrolase | 1654 | 107 | 3.41E-03 |
| Transcription Factor | 1787 | 107 | 2.65E-02 |
| Transferase | 1313 | 88 | 3.08E-03 |
| Enzyme Modulator | 1439 | 73 | 4.40E-01 |
| Receptor | 1597 | 72 | 2.17E-01 |
| Cytoskeletal Protein | 865 | 65 | 7.95E-04 |
| Transporter | 1069 | 64 | 7.36E-02 |
| Oxidoreductase | 639 | 58 | 1.28E-05 |
| Signaling Molecule | 1048 | 48 | 3.15E-01 |
| Dna Binding Protein | 861 | 47 | 2.73E-01 |
| Rna Binding Protein | 983 | 45 | 3.22E-01 |
| Actin Family Cytoskeletal Protein | 424 | 37 | 9.12E-04 |
| G-Protein Modulator | 487 | 37 | 8.37E-03 |
| Zinc Finger Transcription Factor | 682 | 37 | 3.14E-01 |
| Protease | 597 | 36 | 1.38E-01 |
| Ligase | 501 | 33 | 6.53E-02 |
| Kinase | 545 | 31 | 2.45E-01 |
| Phosphatase | 306 | 26 | 6.78E-03 |
| Extracellular Matrix Protein | 466 | 26 | 3.00E-01 |
| Dehydrogenase | 257 | 25 | 1.45E-03 |
| Protein Kinase | 404 | 25 | 1.57E-01 |
| Reductase | 211 | 24 | 2.14E-04 |
| Cell Adhesion Molecule | 593 | 24 | 1.80E-01 |
| Transfer/Carrier Protein | 439 | 22 | 5.11E-01 |
| Ubiquitin-Protein Ligase | 305 | 20 | 1.31E-01 |
| Calcium-Binding Protein | 384 | 20 | 4.45E-01 |
| Krab Box Transcription Factor | 410 | 20 | 5.28E-01 |
| Cation Transporter | 221 | 19 | 1.67E-02 |
| Microtubule Family Cytoskeletal Protein | 250 | 19 | 4.79E-02 |
| Serine Protease | 278 | 19 | 1.05E-01 |
| Non-Receptor Serine/Threonine Protein Kinase | 292 | 19 | 1.45E-01 |
| Protein Phosphatase | 180 | 17 | 1.01E-02 |
| Chromatin/Chromatin-Binding Protein | 190 | 17 | 1.62E-02 |
| Non-Motor Actin Binding Protein | 196 | 16 | 3.92E-02 |
| Ion Channel | 336 | 16 | 4.99E-01 |
| Defense/Immunity Protein | 572 | 15 | 4.09E-03 |
| Guanyl-Nucleotide Exchange Factor | 166 | 15 | 2.12E-02 |
| Nuclease | 200 | 15 | 7.87E-02 |
| Membrane-Bound Signaling Molecule | 224 | 15 | 1.53E-01 |
| Glycosyltransferase | 223 | 14 | 2.24E-01 |
| Intracellular Calcium-Sensing Protein | 262 | 13 | 5.73E-01 |
| Calmodulin | 262 | 13 | 5.73E-01 |
| Methyltransferase | 122 | 12 | 2.10E-02 |
| Cell Junction Protein | 171 | 12 | 1.49E-01 |
| Transcription Cofactor | 195 | 12 | 2.66E-01 |
| Structural Protein | 226 | 12 | 4.47E-01 |
| Basic Helix-Loop-Helix Transcription Factor | 93 | 11 | 7.82E-03 |
| Isomerase | 168 | 11 | 2.18E-01 |
| Kinase Modulator | 292 | 11 | 2.19E-01 |
| Lyase | 183 | 11 | 3.03E-01 |
| Ribosomal Protein | 210 | 11 | 4.70E-01 |
| Chaperone | 221 | 11 | 5.37E-01 |
| Acetyltransferase | 102 | 10 | 3.38E-02 |
| Acyltransferase | 104 | 10 | 3.77E-02 |
| Oxidase | 144 | 10 | 1.84E-01 |
| G-Protein | 219 | 10 | 4.75E-01 |
| G-Protein Coupled Receptor | 433 | 9 | 1.91E-03 |
| Actin Binding Motor Protein | 59 | 9 | 3.20E-03 |
| Membrane Traffic Protein | 386 | 9 | 7.71E-03 |
| Mrna Processing Factor | 316 | 9 | 4.94E-02 |
| Growth Factor | 174 | 9 | 4.96E-01 |
| Cytokine Receptor | 278 | 8 | 6.73E-02 |
| Microtubule Binding Motor Protein | 86 | 8 | 6.83E-02 |
| Mrna Splicing Factor | 271 | 8 | 7.97E-02 |
| Annexin | 124 | 8 | 2.77E-01 |
| Small Gtpase | 135 | 8 | 3.56E-01 |
| Non-Receptor Tyrosine Protein Kinase | 96 | 7 | 2.04E-01 |
| Metalloprotease | 198 | 7 | 2.35E-01 |
| Protease Inhibitor | 193 | 7 | 2.60E-01 |
| Amino Acid Transporter | 109 | 7 | 2.99E-01 |
| Translation Factor | 113 | 7 | 3.31E-01 |
| Voltage-Gated Ion Channel | 136 | 7 | 5.13E-01 |
| Nuclear Hormone Receptor | 48 | 6 | 3.44E-02 |
| Epimerase/Racemase | 58 | 6 | 7.19E-02 |
| Extracellular Matrix Structural Protein | 93 | 6 | 3.17E-01 |
| Helicase | 146 | 6 | 4.13E-01 |
| Cysteine Protease | 125 | 6 | 5.74E-01 |
| Ribonucleoprotein | 123 | 6 | 5.90E-01 |
| Extracellular Matrix Linker Protein | 28 | 5 | 1.38E-02 |
| Homeobox Transcription Factor | 227 | 5 | 3.14E-02 |
| Helix-Turn-Helix Transcription Factor | 227 | 5 | 3.14E-02 |
| Atp-Binding Cassette (Abc) Transporter | 46 | 5 | 8.15E-02 |
| Mitochondrial Carrier Protein | 62 | 5 | 1.98E-01 |
| Non-Motor Microtubule Binding Protein | 64 | 5 | 2.15E-01 |
| Oxygenase | 77 | 5 | 3.36E-01 |
| Extracellular Matrix Glycoprotein | 133 | 5 | 3.54E-01 |
| Rna Helicase | 89 | 5 | 4.52E-01 |
| Immunoglobulin Superfamily Cell Adhesion Molecule | 98 | 5 | 5.36E-01 |
| Ligand-Gated Ion Channel | 109 | 5 | 5.44E-01 |
| Hydroxylase | 17 | 4 | 1.08E-02 |
| Basic Leucine Zipper Transcription Factor | 18 | 4 | 1.31E-02 |
| Creb Transcription Factor | 18 | 4 | 1.31E-02 |
| Immunoglobulin Receptor Superfamily | 180 | 4 | 5.66E-02 |
| Surfactant | 44 | 4 | 1.77E-01 |
| Antibacterial Response Protein | 118 | 4 | 3.04E-01 |
| Nucleotide Kinase | 58 | 4 | 3.25E-01 |
| Kinase Inhibitor | 115 | 4 | 3.26E-01 |
| Phospholipase | 65 | 4 | 4.03E-01 |
| Esterase | 100 | 4 | 4.47E-01 |
| Lipase | 95 | 4 | 4.92E-01 |
| Kinase Activator | 94 | 4 | 5.01E-01 |
| Translation Initiation Factor | 78 | 4 | 5.41E-01 |
| Mrna Polyadenylation Factor | 89 | 4 | 5.48E-01 |
| Transmembrane Receptor Regulatory/Adaptor Protein | 80 | 4 | 5.61E-01 |
| Phosphorylase | 12 | 3 | 2.26E-02 |
| Myelin Protein | 21 | 3 | 8.81E-02 |
| Membrane Trafficking Regulatory Protein | 133 | 3 | 1.04E-01 |
| Tubulin | 23 | 3 | 1.08E-01 |
| Storage Protein | 23 | 3 | 1.08E-01 |
| Aspartic Protease | 24 | 3 | 1.18E-01 |
| Serine Protease Inhibitor | 119 | 3 | 1.59E-01 |
| Chaperonin | 28 | 3 | 1.64E-01 |
| Peptide Hormone | 109 | 3 | 2.12E-01 |
| Anion Channel | 35 | 3 | 2.52E-01 |
| Phosphodiesterase | 36 | 3 | 2.66E-01 |
| Voltage-Gated Sodium Channel | 37 | 3 | 2.79E-01 |
| Sodium Channel | 37 | 3 | 2.79E-01 |
| Calcium Channel | 38 | 3 | 2.92E-01 |
| Voltage-Gated Calcium Channel | 38 | 3 | 2.92E-01 |
| Dehydratase | 40 | 3 | 3.19E-01 |
| Apolipoprotein | 91 | 3 | 3.39E-01 |
| Hmg Box Transcription Factor | 42 | 3 | 3.46E-01 |
| Atp Synthase | 45 | 3 | 3.86E-01 |
| Voltage-Gated Potassium Channel | 85 | 3 | 3.92E-01 |
| Potassium Channel | 85 | 3 | 3.92E-01 |
| Deaminase | 46 | 3 | 3.99E-01 |
| Protein Kinase Receptor | 46 | 3 | 3.99E-01 |
| Dna-Directed Rna Polymerase | 48 | 3 | 4.26E-01 |
| Endoribonuclease | 49 | 3 | 4.39E-01 |
| Adenylate Cyclase | 51 | 3 | 4.64E-01 |
| Cyclase | 55 | 3 | 5.14E-01 |
| Histone | 57 | 3 | 5.38E-01 |
| Cytokine | 178 | 2 | 6.97E-03 |
| Translation Release Factor | 7 | 2 | 4.80E-02 |
| Cadherin | 109 | 2 | 9.36E-02 |
| Tyrosine Protein Kinase Receptor | 17 | 2 | 2.07E-01 |
| Intermediate Filament | 83 | 2 | 2.21E-01 |
| Guanylate Cyclase | 18 | 2 | 2.25E-01 |
| Dna Methyltransferase | 18 | 2 | 2.25E-01 |
| Nucleotidyltransferase | 81 | 2 | 2.35E-01 |
| Rna Methyltransferase | 19 | 2 | 2.43E-01 |
| Endodeoxyribonuclease | 20 | 2 | 2.61E-01 |
| Dna Helicase | 74 | 2 | 2.90E-01 |
| Tumor Necrosis Factor Receptor | 23 | 2 | 3.16E-01 |
| Peroxidase | 24 | 2 | 3.34E-01 |
| Replication Origin Binding Protein | 25 | 2 | 3.52E-01 |
| Decarboxylase | 26 | 2 | 3.70E-01 |
| Transaminase | 26 | 2 | 3.70E-01 |
| Carbohydrate Transporter | 60 | 2 | 4.28E-01 |
| Type I Cytokine Receptor | 59 | 2 | 4.39E-01 |
| Actin And Actin Related Protein | 31 | 2 | 4.55E-01 |
| Exoribonuclease | 32 | 2 | 4.71E-01 |
| Nucleotide Phosphatase | 34 | 2 | 5.03E-01 |
| Acetylcholine Receptor | 46 | 2 | 6.01E-01 |
| Gaba Receptor | 46 | 2 | 6.01E-01 |
| Translation Elongation Factor | 44 | 2 | 6.27E-01 |
| Glycosidase | 42 | 2 | 6.54E-01 |
| Primase | 1 | 1 | 4.84E-02 |
| Dna Polymerase Processivity Factor | 1 | 1 | 4.84E-02 |
| Complement Component | 81 | 1 | 8.98E-02 |
| Chemokine | 50 | 1 | 2.91E-01 |
| Vesicle Coat Protein | 42 | 1 | 3.83E-01 |
| Mutase | 12 | 1 | 4.49E-01 |
| Tight Junction | 36 | 1 | 4.67E-01 |
| Carbohydrate Phosphatase | 13 | 1 | 4.75E-01 |
| Type Ii Cytokine Receptor | 13 | 1 | 4.75E-01 |
| Carbohydrate Kinase | 35 | 1 | 4.82E-01 |
| Centromere Dna-Binding Protein | 17 | 1 | 5.70E-01 |
| Hsp70 Family Chaperone | 17 | 1 | 5.70E-01 |
| Dna-Directed Dna Polymerase | 29 | 1 | 5.78E-01 |
| Cyclic Nucleotide-Gated Ion Channel | 18 | 1 | 5.91E-01 |
| Hydratase | 19 | 1 | 6.11E-01 |
| Deacetylase | 27 | 1 | 6.13E-01 |
| Serine/Threonine Protein Kinase Receptor | 26 | 1 | 6.30E-01 |
| Neuropeptide | 25 | 1 | 6.48E-01 |
| Ionotropic Glutamate Receptor | 23 | 1 | 6.84E-01 |
| Cysteine Protease Inhibitor | 21 | 1 | 7.20E-01 |
| Gap Junction | 21 | 1 | 7.20E-01 |

Statistical significance of treatment: p < 0.05
